# Nosokomiale Infektionen und Antibiotikaanwendung in Langzeitpflegeeinrichtungen. Deutsche Ergebnisse der dritten europäischen Punkt-Prävalenz-Erhebung HALT-3

**DOI:** 10.1007/s00103-022-03566-3

**Published:** 2022-08-11

**Authors:** Nicole Schmidt, Vanda Marujo, Tim Eckmanns, Benedikt Zacher, Mardjan Arvand, Claudia Ruscher

**Affiliations:** 1 Fachgebiet 37 – Nosokomiale Infektionen, Surveillance von Antibiotikaresistenz und -verbrauch, Robert Koch-Institut, Seestr. 10, 13353 Berlin, Deutschland; 2 Fachgebiet 14 – Angewandte Infektions- und Krankenhaushygiene, Robert Koch-Institut, Berlin, Deutschland; 3 Landesamt für Gesundheit und Soziales (LAGeSo), Berlin, Deutschland

**Keywords:** Pflegeheim, Nosokomiale Infektionen, Antibiotikaanwendung, Infektionsprävention, Ältere Menschen, Long-term care, Nosocomial infections, Antimicrobial use, Infection prevention and control, Elderly

## Abstract

**Hintergrund und Ziel:**

Die wichtige Rolle der Maßnahmen zur Infektionsprävention und -kontrolle in Langzeitpflegeeinrichtungen ist im Kontext der aktuellen SARS-CoV-2-Pandemie besonders deutlich geworden. Um eine verlässliche Datenbasis zu nosokomialen Infektionen und Antibiotikaanwendung zu gewinnen, hat das European Centre for Disease Prevention and Control (ECDC) 2016–2017 die dritte Punkt-Prävalenz-Erhebung in europäischen Langzeitpflegeeinrichtungen (HALT-3) initiiert.

**Material und Methoden:**

In Deutschland nahmen 131 Einrichtungen mit 10.565 Bewohnern teil. An einem Stichtag 2016 wurden die Anzahl von nosokomialen Infektionen, die Antibiotikaanwendung sowie Pflegecharakteristika und Risikofaktoren der Bewohner erhoben. Infektionen wurden symptombasiert anhand von Algorithmen entsprechend der McGeer-Surveillance-Definitionen für Pflegeeinrichtungen erfasst.

**Ergebnisse:**

Bei 177 Bewohnern wurde eine nosokomiale Infektion dokumentiert, was einer Prävalenz von 1,7 % (95 %-KI: 1,3–2,1) entspricht und im europäischen Vergleich (Mittelwert 3,9 %) ein eher niedriger Wert ist. Harnwegsinfektionen waren mit knapp 50 % die häufigsten Infektionen, gefolgt von Atemwegs‑, Haut- und Weichgewebeinfektionen. Die häufigsten Indikationen für eine Antibiotikaanwendung entsprechen den erfassten Infektionen. Bei 143 Bewohnern wurde eine Antibiotikaanwendung dokumentiert (Prävalenz 1,4 %, 95 %-KI: 1,1–1,7). Auffällig war der hohe Einsatz von Fluorchinolonen mit über 20 % aller Verordnungen.

**Diskussion:**

Der Aufbau einer einrichtungsbasierten Surveillance von nosokomialen Infektionen und Antibiotikaanwendung könnte zusammen mit der Zurverfügungstellung von spezifisch auf die geriatrische Population zugeschnittenen Leitlinien zur Verbesserung der Infektionsprävention und zum rationaleren Einsatz von Antibiotika beitragen sowie die Qualität und Sicherheit in der Pflege erhöhen.

**Zusatzmaterial online:**

Zusätzliche Informationen sind in der Online-Version dieses Artikels (10.1007/s00103-022-03566-3) enthalten.

## Abstract (en)

**Background and objective:**

The essential role of infection prevention in long-term care facilities has become evident during the current SARS-CoV‑2 pandemic. In order to obtain a reliable database on nosocomial infections and antibiotic use, the European Centre for Disease Prevention and Control (ECDC) initiated the third point prevalence survey in European long-term care facilities from 2016 to 2017 (HALT-3).

**Material and methods:**

In Germany, 131 facilities with 10,565 residents participated voluntarily. On a single day in 2016, the number of nosocomial infections and/or uses of antibiotics as well as care characteristics and risk factors of the residents were recorded. Infections were documented based on symptoms using an algorithm in accordance with the McGeer surveillance criteria for long-term care facilities.

**Results:**

A nosocomial infection was documented in 177 residents, which corresponds to a prevalence of 1.7% (95% CI: 1.3–2.1), still low in comparison with the European prevalence (mean value 3.9%). Urinary tract infections were the most common infections at almost 50%, followed by respiratory, skin, and soft-tissue infections. The type of infection was consistent with the most common indications for antibiotic use. Antibiotic use was documented in 143 residents (prevalence of 1.4%, 95% CI: 1.1–1.7). The frequent use of fluoroquinolones with over 20% of all prescriptions was noticeable.

**Conclusions:**

The establishment of facility-based surveillance of the most common infections and antibiotic consumption, together with the creation of guidelines specifically tailored to the geriatric population, could contribute to improving infection prevention and control as well as a more rational use of antibiotics, thus increasing the quality and safety of care.

## Hintergrund

Menschen über 65 Jahre stellen in Deutschland einen immer größeren Anteil an der Gesamtbevölkerung. Dieser stieg im Jahr 2019 auf 22 % und ist der fünfthöchste in der Europäischen Union, wo er im Durchschnitt bei 20,3 % liegt. Gemäß Angaben des statistischen Bundesamts wurden in Deutschland Ende 2019 ca. 820.000 Pflegebedürftige in 11.317 vollstationären Langzeitpflegeeinrichtungen (engl.: „long-term care facility“, LTCF) versorgt. Davon waren 70 % Frauen, 93 % über 65 Jahre alt und die Hälfte war mindestens 85 Jahre alt. Der Anteil der Pflegebedürftigen mit dem höchsten Pflegegrad 5 lag bei 15 %. Die Alltagskompetenz war oft eingeschränkt [1].

Die Vulnerabilität für Infektionen ist bei Pflegebedürftigen aufgrund von allgemeiner Gebrechlichkeit und altersbedingten Dysfunktionen, wie einer verminderten Immunantwort oder funktionellen Beeinträchtigungen, erhöht. Hinzu kommen chronische Erkrankungen und häufig eine sekundäre Immundysfunktion durch Malnutrition und Polypharmazie (z. B. immunsuppressive Medikamente) [2]. Bei älteren Menschen können typische Infektionszeichen fehlen, was zu Verzögerungen beim Erkennen einer Infektion führen kann. Bewohner teilen dieselben Räumlichkeiten (z. B. Gemeinschaftsräume) und die pflegerische Versorgung, sodass Ausbrüche in diesem Setting häufiger auftreten, dies wurde in der aktuellen COVID-19-Pandemie eindrücklich sichtbar [3].

Der zunehmende ökonomische Druck auf Krankenhäuser führt u. a. zu kürzeren Verweildauern (2017: 7,2 Tage; 1992: 13,3 Tage im Durchschnitt) und verlagert die Weiterbetreuung der oft multimorbiden Patienten in die LTCF [4]. Infektionen werden häufig durch multiresistente Erreger (MRE) verursacht und es gibt zunehmend Evidenz dafür, dass LTCF ein Ort für Transmissionen sein können. Bestätigt wird dies auch durch Daten zu Verlegungen zwischen dem Krankenhaus und der LTCF und umgekehrt [5–7]. Die jährliche Gesamtzahl der nosokomialen Infektionen (NI) in Europa wurde 2018 vom European Centre for Disease Prevention and Control (ECDC) anhand der HALT-3-Ergebnisse auf 8,9 Mio. geschätzt, davon 4,5 Mio. in Krankenhäusern (KI 2,6–7,6 Mio.) und 4,4 Mio. in LTCF (KI 2,0–8,0 Mio.) [8, 9]. Dadurch steigen die Anforderungen für die diagnostische, therapeutische und medizinisch-pflegerische Versorgung, insbesondere bekommen Maßnahmen der Infektionsprävention und -kontrolle (engl.: Infection Prevention and Control, IPC) einen höheren Stellenwert und die Pflegekräfte sehen sich einer steigenden Arbeitsbelastung ausgesetzt. Erschwerend kommt der bereits bestehende Personalmangel in der Pflege hinzu: Im Jahr 2018 wurden 17.000 unbesetzte Stellen in Deutschland registriert, darunter 11.400 für Pflegekräfte in LTCF [10].

Für Krankenhäuser wurde mit Einführung des § 23 des Infektionsschutzgesetzes (IfSG) eine Gesetzesgrundlage für die verpflichtende kontinuierliche Erfassung und Bewertung von NI und Antibiotikaanwendung (ABA) geschaffen, für LTCF existiert diese nicht. Daten zur Situation in LTCF werden somit vor allem im Rahmen von Prävalenzstudien wie dieser in ausgewählten Stichproben gewonnen.

Das ECDC hat seit 2010 3 Punkt-Prävalenz-Erhebungen (PPS) zu NI und ABA in LTCF in Europa initiiert (HALT: „Healthcare-associated infections in long-term care facilities“). Deutschland hat an allen dreien teilgenommen [11–13]. Die letzte fand 2016–2017 als „HALT-3“ statt, 26 Länder mit 3062 LTCF nahmen daran teil. Dieser Artikel präsentiert die Ergebnisse für Deutschland.

## Methoden

### Studiendesign, Studienpopulation und Rekrutierung.

Das ECDC koordiniert die PPS und hat ein europäisch einheitliches Studienprotokoll erarbeitet [14]. Das übersetzte Studienprotokoll für Deutschland ist im Onlinematerial 1 zu diesem Artikel verfügbar. Die Teilnahme war freiwillig und die Studie wurde in Deutschland vom HALT-3-Team am Robert Koch-Institut (RKI) koordiniert.

Es konnten LTCF teilnehmen, in denen Bewohnern dauerhaft (24/24 h) qualifizierte Pflege zur Verfügung steht. Alle Bewohner, die am Tag der Erhebung in der Einrichtung waren, wurden in die Studie eingeschlossen. Die Rekrutierung der teilnehmenden LTCF erfolgte über die Gesundheitsämter und unter Einbindung der regionalen MRE-Netzwerke als „convenience sampling“ (willkürliche Stichprobenerhebung mit eingeschränkter Generalisierbarkeit).

### Datenerhebung und Datenmanagement.

Aufgrund der Komplexität in der Methodik war eine eintägige Schulung Voraussetzung für die Teilnahme. Studien- und Schulungsmaterial zum einheitlichen Erkennen und Erfassen von Infektionen stellte das ECDC zur Verfügung, die Anpassung, Übersetzung und Aufarbeitung wurde durch das HALT-3-Team am RKI vorgenommen. Die Bewohner wurden vor Ort durch geschulte Mitarbeiter der LTCF auf Anzeichen und Symptome für Infektionen untersucht und Datenblätter, Krankenakten sowie die dokumentierten Arzneimittelverordnungen überprüft. Neben Antibiotika wurde auch die Anwendung von Medikamenten gegen Pilze, Viren und Parasiten mituntersucht. Infektionen wurden mithilfe von Algorithmen entsprechend den Surveillance-Definitionen zur Bewertung von NI in LTCF nach McGeer et al. erfasst (s. Onlinematerial 2) [15, 16].

Ein *Einrichtungsfragebogen* (s. Onlinematerial 3) diente der Erfassung struktureller Parameter wie Größe und Art der Einrichtung sowie der Nennerdaten zur Gesamtzahl der Bewohner zur Häufigkeit von Pflegecharakteristika und Risikofaktoren wie Anzahl von Bewohnern: > 85 Jahre, mit weiblichem/männlichem Geschlecht, mit Operationen innerhalb der letzten 30 Tage, mit Harnwegs- oder Gefäßkatheter, mit Inkontinenz, mit Zustand der Desorientiertheit, mit eingeschränkter Mobilität wie Bettlägerigkeit oder Nutzung eines Rollstuhls, mit Dekubitus und Angaben zur medizinischen Versorgung, zu IPC einschließlich Strategien zum Antibiotikaeinsatz.

Der *Bewohnerfragebogen* (s. Onlinematerial 4) wurde für jeden Bewohner, der am Tag der PPS ein systemisches Antibiotikum erhielt und/oder Anzeichen und Symptome einer aktiven NI zeigte, angelegt. Darin erfasst wurden die o. g. Pflegecharakteristika, Aufenthaltsdauer in der LTCF (länger als ein Jahr), Krankenhausaufenthalte innerhalb der letzten 3 Monate sowie Angaben zu nachgewiesenen Mikroorganismen und antimikrobieller Resistenz bei der dokumentierten NI. Es wurden nur mikrobiologische Befunde erfasst, die am Erhebungstag verfügbar waren.

Die LTCF wurden pseudonymisiert, die ausgefüllten Fragebögen an das RKI gesendet. Dort wurden sie in eine elektronische Datenbank übertragen. Statistische Analysen wurden unter Verwendung der open source Software R 3.5.0 durchgeführt. Die Datenbank wurde an das ECDC zur Auswertung übermittelt und für jede teilnehmende LTCF wurde ein detaillierter Bericht mit den Vergleichsdaten aller LTCF erstellt.

### Endpunkte der Studie.

- *Gesamtprävalenz NI:* Auch in anderen LTCF oder im Krankenhaus erworbene NI werden erfasst und auf alle in die Studie eingeschlossenen Bewohner bezogen. Anhand dieser Prävalenz lässt sich die Gesamtbelastung abschätzen.
- *Prävalenz der NI, die in der eigenen Einrichtung erworben wurden:* Anteil der Bewohner (in %) mit NI am Erhebungstag, bei denen die NI in der eigenen Einrichtung erworben wurde, bezogen auf alle in die Studie eingeschlossenen Bewohner. Damit sind Vergleiche zwischen den LTCF möglich.
- *Prävalenz ABA:* Anteil der Bewohner (in %) mit dokumentierter ABA am Erhebungstag bezogen auf alle in die Studie eingeschlossenen Bewohner.

## Ergebnisse

### Beteiligung und Strukturdaten der LTCF.

Durch die MRE-Netzwerke und die Gesundheitsämter konnte bei 250 LTCF Interesse für die PPS geweckt werden. An den Schulungen als verbindlichem Bestandteil nahmen 163 LTCF teil. Es übermittelten 132 LTCF Daten an das RKI, von denen für 131 eine Auswertung erfolgen konnte. 1 LTCF musste aufgrund der fehlerhaften Angabe der Studiennummer ausgeschlossen werden. Die LTCF verteilten sich hauptsächlich auf die südwestlichen Bundesländer (s. Onlinematerial 5).

Die PPS in den LTCF wurde überwiegend von Pflegekräften (*n* = 100; 76,3 %) durchgeführt, in 1 LTCF war es ein Arzt. 30 LTCF machten dazu keine Angaben. Teilnehmende waren, bis auf eine psychiatrische Einrichtung, allgemeine bzw. gemischte Pflege- und Senioreneinrichtungen, in welchen Bewohner mit verschiedenen Pflegegraden betreut werden. Diese LTCF hatten 9 bis 286 Betten, die mediane Bettenanzahl lag bei 80 und der mediane Anteil an Einzelzimmern betrug 79,3 % (s. Onlinematerial 6). Insgesamt konnten 10.565 Bewohner in die Studie eingeschlossen werden.

### Medizinische Versorgung, Koordination und Ausstattung mit Hygienefachpersonal.

Die medizinische Versorgung der Bewohner wurde in den LTCF mehrheitlich durch individuelle Hausärzte gewährleistet (130 von 131 LTCF). 28 LTCF (21,4 %) gaben an, medizinische Maßnahmen zusätzlich durch externe Ärzte koordinieren zu lassen, wobei 2 von diesen gleichzeitig auch in der LTCF angestellt waren. 114 LTCF (87 %) gaben an, dass es bei ihnen eine in IPC geschulte Person zur Unterstützung des Pflegepersonals gibt, bei 17 LTCF (13,0 %) gab es keine. Die geschulten Personen waren überwiegend examinierte Pflegekräfte (*n* = 99; 86,8 %), in einem Fall war es ein Arzt. In 11 LTCF standen sowohl Pflegekräfte als auch Ärzte zur Verfügung, 3 LTCF machten dazu keine Angaben. In 100 LTCF (76,3 %) war eine Hygienekommission etabliert und in 86 (87,8 %) LTCF trat diese zweimal pro Jahr zusammen. Die Möglichkeit, eine externe infektionshygienische Beratung einzuholen, bestand in 103 LTCF (78,6 %).

### Händehygiene.

Fast alle LTCF gaben an, im vergangenen Jahr Fortbildungen zur Händehygiene für das Personal durchgeführt zu haben (128; 97,7 %). 126 LTCF (96,2 %) machten die Angabe, dass alkoholisches Händedesinfektionsmittel eingesetzt wurde und 105 LTCF (80,2 %) gaben auch die Verbrauchsmenge an. Im Median wurden von den LTCF 8,4 ml Desinfektionsmittel pro Bewohnertag verbraucht. Legt man die Menge von 3 ml Desinfektionsmittel pro durchgeführter Händedesinfektion zugrunde, entspricht dies 2,8 Händedesinfektionen pro Bewohnertag. Es fiel eine große Spannbreite von 0,4–126,4 ml pro Bewohnertag zwischen den LTCF auf.

### Infektionspräventionskonzepte, Surveillance und Antibiotic-Stewardship-Strategien.

**Figure 1:**
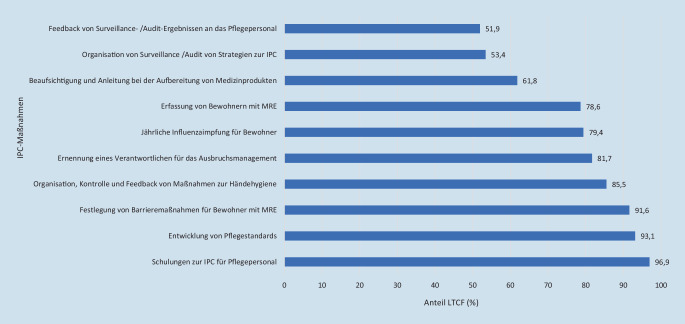

Zu den in den LTCF gut etablierten Konzepten und Strategien zur IPC gehören Schulungen für Pflegekräfte in IPC (96,9 %), die Entwicklung von Pflegestandards (93,1 %) und existierende Arbeitsanweisungen zu Barrieremaßnahmen für Bewohner mit MRE (91,6 %; Abb. 1). Die regelmäßige Organisation von Surveillance/Audit von Strategien zur IPC und das Feedback dieser Ergebnisse an das Pflegepersonal sind bisher nur in knapp mehr als der Hälfte der LTCF etabliert.

6 LTCF (4,6 %) gaben an, dass ein Surveillance-System zur Erfassung und Bewertung des Antibiotikaverbrauchs vorhanden ist, aber nur 1 LTCF berichtete darüber hinaus, dass Daten zum jährlichen Antibiotikaverbrauch nach Wirkstoffgruppen verfügbar sind. In dieser LTCF existierte auch ein System zur Erfassung von MRE wie dem Methicillin-resistenten Staphylococcus aureus (MRSA). Insgesamt gaben 34 LTCF (26,0 %) an, dass sie MRE erfassen.

### Pflegecharakteristika und Risikofaktoren der Bewohner.

Am Tag der PPS erfüllten 10.565 Bewohner in 131 LTCF die Einschlusskriterien, die Hälfte war älter als 85 Jahre und knapp 3 Viertel waren Frauen. Inkontinenz, zeitliche und/oder örtliche Desorientiertheit waren häufige Pflegecharakteristika, weitere Charakteristika und Risikofaktoren werden in Tab. 1 aufgezeigt.

**Table 1:** 

Pflegecharakteristika und Risikofaktoren	Anzahl	Anteil (%)
Alter > 85 Jahre	5318	50,3
Weibliches Geschlecht	7669	72,6
Männliches Geschlecht	2896	27,4
Inkontinenz	7285	69,0
Zeitliche und/oder örtliche Desorientiertheit	5811	55,0
Eingeschränkte Mobilität	4738	44,8
Operation in den letzten 30 Tagen	181	1,7
Harnwegskatheter	952	9,0
Gefäßkatheter	33	0,3
Dekubitus	412	3,9
Andere Wunden	793	7,5

### Pflegecharakteristika und Risikofaktoren der Bewohner mit einer nosokomialen Infektion.

Im Einrichtungsfragebogen wurden für alle in die Studie eingeschlossenen Bewohner die Pflegecharakteristika und Risikofaktoren aggregiert abgefragt. Zusätzlich wurde für Bewohner mit einer NI und/oder einer ABA ein Bewohnerfragebogen angelegt, in welchem die Pflegecharakteristika und Risikofaktoren bewohnerbezogen erfasst wurden. Von den 226 Bewohnern mit NI und/oder ABA war bei 66 (29,2 %) ein Aufenthalt in einem Krankenhaus in den letzten 3 Monaten dokumentiert.

**Figure 2:**
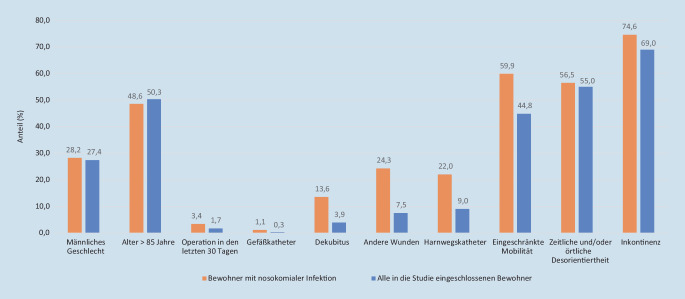

In der Häufigkeit der Risikofaktoren Geschlecht und Alter > 85 Jahre sowie des Pflegecharakteristikums einer örtlichen/zeitlichen Desorientiertheit zeigten die Bewohner mit NI kaum Unterschiede im Vergleich zur Gesamtgruppe. Die Anteile verschiedener Pflegecharakteristika und Risikofaktoren sind in der Abb. 2 dargestellt.

### Nosokomiale Infektionen.

**Figure 3:**
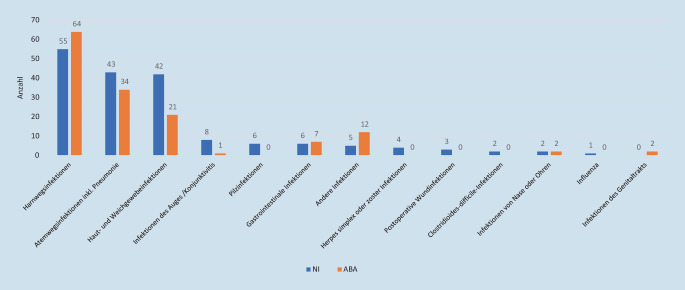

Bei 177 der 10.565 Bewohnern wurde eine NI dokumentiert, dies entspricht einer Gesamtprävalenz von 1,7 % (95-%-KI: 1,3–2,1), davon erhielten 94 (41,6 %) eine ABA und 83 (36,7 %) keine. 138 NI wurden als in der eigenen Einrichtung erworben deklariert, was einer Prävalenz von 1,3 % (95-%-KI: 1,0–1,7) entspricht. Am häufigsten wurden Infektionen der Harnwege (*n* = 55; 31,1 %), der Atemwege (*n* = 43; 24,3 %) sowie der Haut- und Weichgewebe (*n* = 42; 23,7 %) dokumentiert (Abb. 3). 39 NI wurden als „mitgebracht“ klassifiziert, der Ursprung der NI lag also in einer anderen Einrichtung: Krankenhaus (*n* = 24), andere LTCF (*n* = 1), unbekannt (*n* = 9), keine Angabe (*n* = 5). Von den 41 in der eigenen Einrichtung dokumentierten Harnwegsinfektionen lag am Tag der PPS für 10 Bewohner (24,4 %) ein mikrobiologischer Befund vor.

### Nachgewiesene Erreger und Resistenz.

Es gab für 21 Bewohner Angaben zu einer mikrobiologischen Diagnostik. Insgesamt wurden 22 verschiedene Arten von Erregern (davon 2 Viren und 1 Pilz) identifiziert, am häufigsten fanden sich *Escherichia coli* (*n* = 6), *Streptococcus pneumoniae* (*n* = 3), *Pseudomonas aeruginosa* (*n* = 3), *Staphylococcus aureus* (*n* = 2) und *Clostridioides difficile* (*n* = 2). Die 10 dokumentierten Resistenztestungen erfolgten aus dem Urin (*n* = 6) und aus Haut- bzw. Wundgewebe (*n* = 4), für 2 wurde das Ergebnis der Testung als „sensibel“ angegeben und für die übrigen als „unbekannt“ (*n* = 7) dokumentiert. Die Hälfte (*n* = 5) der vorliegenden Befunde zu Resistenztestungen stammte von Bewohnern, deren NI ihren Ursprung in einem Krankenhaus hatte. Das Ergebnis der Resistenztestung war in diesen Fällen als „unbekannt“ eingetragen worden.

### Antibiotikaanwendung.

Bei 143 Bewohnern wurde eine ABA erfasst, dieses entspricht einer Prävalenz von 1,4 % (95-%-KI: 1,1–1,7). Von den 143 ABA wurde bei 94 Bewohnern eine NI dokumentiert, bei 49 (21,7 %) nicht. Die ABA wurde überwiegend in der Einrichtung selbst verordnet (*n* = 119; 83,2 %), 11 LTCF gaben als Ort der Verordnung ein Krankenhaus an, 7 einen anderen Ort und 6 machten keine Angabe. 134 Bewohner (93,7 %) erhielten die ABA als Therapie, 9 (6,3 %) zur Prophylaxe. Die häufigste Indikation stellten Harnwegs- (*n* = 64; 44,8 %), Atemwegs- (*n* = 34; 23,8 %) sowie Haut- und Weichgewebeinfektionen (*n* = 21; 14,7 %) dar (Abb. 3). Eine prophylaktische ABA erfolgte am häufigsten im Zusammenhang mit einer vorangegangenen Harnwegsinfektion. Bei 96 von 134 (75,4 %) Bewohnern mit therapeutischer ABA wurde das Enddatum der Einnahme in der Akte dokumentiert. Für die prophylaktische ABA war es nur in gut der Hälfte der Fälle (*n* = 5) angegeben. Die Applikation erfolgt fast ausschließlich oral, es wurden nur eine parenterale und eine inhalative Gabe dokumentiert.

Für die Behandlung von Harnwegsinfektionen wurden am häufigsten Fluorchinolone (31,3 %) verordnet, für Atemwegsinfektionen Penicilline (38,2 %) und Cephalosporine (26,5 %) und für Haut- und Weichgewebeinfektionen Fluorchinolone (42,9 %) und Penicilline (28,6 %; Tab. 2).

**Table 2:** 

*Bewohner mit einer ABA aufgrund einer Harnwegsinfektion*	*Anzahl (n* *=* *64)*	*Anteil (%)*
Fluorchinolone	20	31,3
Cotrimoxazol und andere Sulfonamide	15	23,4
Nitrofurantoin	11	17,2
Fosfomycin	4	6,3
Cephalosporine	5	7,8
Penicillin	3	4,7
Trimethoprim	2	3,1
Nitroxolin	2	3,1
Pivmecillinam	1	1,6
Doxycyclin	1	1,6
*Bewohner mit einer ABA aufgrund einer Atemwegsinfektion inkl. Pneumonie*	*Anzahl (n* *=* *34)*	*Anteil (%)*
Penicilline	13	38,2
Cephalosporine	9	26,5
Fluorchinolone	4	11,8
Tetracycline	5	14,7
Andere	3	8,8
*Bewohner mit einer ABA aufgrund einer Haut- und Weichgewebeinfektion*	*Anzahl (n* *=* *21)*	*Anteil (%)*
Fluorchinolone	9	42,9
Penicilline	6	28,6
Lincosamide	3	14,3
Andere	3	14,3

*ABA* Antibiotikaanwendung

## Diskussion

Die HALT-3-Erhebung im Jahr 2016 konnte mit 131 LTCF und 10.565 Bewohnern nach HALT (2010) und HALT‑2 (2013) erneut wichtige Daten zum Vorkommen von NI und ABA sowie zu IPC-Strategien in deutschen LTCF generieren. Im Gegensatz zu Krankenhäusern gibt es für LTCF keine gesetzliche Grundlage zur kontinuierlichen Erfassung von NI und ABA, sodass eine valide und repräsentative Datenbasis fehlt, welche aber durch wiederholte Erhebungen dieser Art gewonnen werden kann. Damit soll ein einfaches Tool zur Verfügung gestellt werden, welches ermöglicht NI und ABA in den LTCF zu erfassen und zu bewerten, aber auch den Ländern die Möglichkeit gibt, Entwicklungen in diesen Bereichen zu verfolgen und prioritäre Maßnahmen für nationale und lokale Interventionen zu erkennen.

### Nosokomiale Infektionen.

In Deutschland liegt die Prävalenz von NI in den an HALT‑3 teilnehmenden LTCF mit 1,7 % (95 %-KI: 1,3–2,1) weiterhin relativ niedrig (HALT-2: 1,7 %, HALT: 0,79 %). In den an HALT‑3 teilnehmenden europäischen LTCF ergab sich unter Berücksichtigung der Validierungsergebnisse eine höhere Prävalenz von 3,9 % [9, 17], was am ehesten mit Unterschieden in der Zusammensetzung der Bewohnerpopulation (Case-Mix) sowie in der Art der Einrichtung zu erklären ist. So wurden in Spanien Bewohner eingeschlossen, welche im Anschluss an eine Akutbehandlung im Krankenhaus in der LTCF weiterbetreut werden („post-acute care“). Im Rahmen der Auswertung der europäischen Gesamtergebnisse von HALT‑3 durch das ECDC veröffentlichten Suetens et al. Schätzungen zur Gesamtzahl von NI in LTCF an einem bestimmten Tag. Es wurden nur 82 der 131 LTCF ausgewertet, um eine Überrepräsentation der deutschen Ergebnisse zu vermeiden. Für Deutschland ergab sich für die 10.389 vollstationären LTCF mit ungefähr 852.850 Betten eine geschätzte Gesamtzahl von 13.936 (95 %-KI: 10.209–18.878) NI pro Tag [9].

Bei den dokumentierten NI in HALT‑3 handelt es sich wie bereits in HALT‑2 am häufigsten um Harnwegs‑, Atemwegs- und Haut- und Weichgewebeinfektionen. Von den 41 in der eigenen Einrichtung dokumentierten Harnwegsinfektionen lag am Tag der PPS für 10 Bewohner (24,4 %) ein mikrobiologischer Befund vor. Es gehen nur solche Befunde in die Auswertung, die am Tag der PPS in der LTCF vorliegen, sodass die tatsächliche Anzahl der mikrobiologischen Befunderhebungen höher sein könnte. Insgesamt deuten unsere Daten darauf hin, dass eine solche Diagnostik in diesem Setting eher selten durchgeführt wird. Die Diagnostik ist jedoch auch bei unkomplizierter Zystitis für diese vulnerable Gruppe mit Komorbiditäten wichtig, wenn die Folgen einer verzögerten/inkorrekten Therapie als schwerwiegend eingeschätzt werden (Stichwort Morbidität/Mortalität). Nur so kann die Therapie an die antimikrobielle Empfindlichkeitsprüfung angepasst werden [18].

### Händehygiene und Infektionspräventionsstrategien.

Regelmäßige und korrekte Händehygiene gilt als zentrale evidenzbasierte Maßnahme zur Prävention der Transmission von Infektionserregern und der Entstehung von NI. In den meisten LTCF ist das Thema Händehygiene etabliert und es finden jährliche Schulungen statt. Vom ECDC wurden seit 2010 3 Schlüsselkriterien zur Einschätzung der Strategie zur IPC abgefragt: die Ausstattung einer Einrichtung mit Hygienefachpersonal, das Vorhandensein einer Hygienekommission und der Zugang zu externer Expertise (infektionshygienische Beratung) [19, 20]. Diese waren in den teilnehmenden LTCF nur zum Teil umgesetzt, was Potenzial für Verbesserungen aufzeigt. Auffällig sind die positiven Angaben der Heime. Ursachen dafür könnten eine Überschätzung, ein unzureichendes Verständnis oder auch ein Selektionsbias sein. Ein direkter Vergleich der eingesetzten IPC-Maßnahmen mit anderen Ländern gestaltet sich schwierig, da die Strukturen je nach Bewohnerkollektiv teilweise unterschiedlich sind ebenso wie die Qualifizierung des Pflegepersonals [9].

Eine Stärkung der Expertise zu IPC in LTCF ist wichtig. Sie könnte durch Schulungsmaßnahmen für das Personal, aber auch durch strukturelle Anpassungen wie das Hinzuziehen einer externen infektionshygienischen Beratung oder die Anbindung der Einrichtung an ein Krankenhaus mit einem Fachbereich für Hygiene umgesetzt werden. Der Zusammenhang zwischen IPC-Personalausstattung bzw. -Maßnahmen und Infektionsprävalenz wurde nicht untersucht, könnte aber in einer zukünftigen Studie aufgenommen werden.

### Antibiotikaanwendung.

Die Prävalenz der ABA lag in HALT‑3 mit 1,4 % etwas höher als in HALT‑2 (1,1 %). Bei Betrachtung aller an HALT‑3 teilnehmenden europäischen LTCF lag die Prävalenz der ABA in LTCF bei 4,9 % und in der deutschen nationalen PPS in Krankenhäusern erwartungsgemäß deutlich höher bei 25,9 % [21, 22]. Die meisten ABA waren therapeutisch (*n* = 134; 93,7 %), nur in 9 (6,3 %) Fällen prophylaktisch. Bei HALT‑2 lag der Anteil der prophylaktischen ABA noch doppelt so hoch bei 12,6 % [13]. Dies weist auf einen abnehmenden Trend hin, wobei in Deutschland die prophylaktische ABA ohnehin vergleichsweise selten stattfindet – in den gesamteuropäischen HALT-3-Ergebnissen wurde die prophylaktische ABA in einem Drittel der Fälle eingesetzt [17].

Bei 96 von 134 Bewohnern mit therapeutischer ABA (75,4 %) wurde das Enddatum der Einnahme in der Akte dokumentiert – etwas häufiger als die europäischen HALT‑3 Ergebnisse zeigen (66,7 %). Bemerkenswert ist, dass immer noch bei einem Viertel der ABA kein Enddatum dokumentiert wurde, obwohl dies ein einfach umsetzbarer Qualitätsindikator für eine rationale Antibiotikaverordnung im Rahmen von Antibiotic Stewardship (ABS) ist [21]. ABS-Fortbildungen für Ärzte könnten zu einem rationaleren Einsatz beitragen, jedoch ist das Angebot von Fortbildungen, welche spezifisch auf die Erfordernisse des ambulanten Bereichs und die Versorgung von Menschen in LTCF zugeschnitten sind, rar [23, 24].

Bei den verwendeten Antibiotikaklassen zeigt sich, dass Fluorchinolone, wie schon im Jahr 2013, am häufigsten bei den Indikationen Harnwegs‑, Haut- und Weichgewebeinfektion verordnet wurden. Ciprofloxacin bleibt insgesamt der am häufigsten verordnete Wirkstoff, obwohl Fluorchinolone bei Personen im höheren Alter vor allem wegen zerebraler Nebenwirkungen wie Verwirrtheit, Delir und Halluzinationen mit besonderer Vorsicht angewendet werden sollen und bei vorhandener Alternative dieser der Vorzug gegeben werden soll. Seitens des Bundesinstituts für Arzneimittel und Medizinprodukte (BfArM) und der Europäischen Arzneimittelagentur (EMA) wurde 2018 von einem Einsatz von Chinolonen für leichte bis mittelschwere Harn- und Atemwegsinfektionen abgeraten [25]. In der nächsten Erhebung, die für 2023 geplant ist, wird sich vielleicht schon eine Trendwende zeigen.

Am häufigsten wurde eine ABA aufgrund einer Harnwegsinfektion verordnet, aus den Daten ist jedoch nicht ersichtlich, ob es sich um unkomplizierte oder komplizierte Harnwegsinfektionen handelt. In 6 Fällen erfolgte eine mikrobiologische Urindiagnostik, das Ergebnis der Resistenztestung wurde als „unbekannt“ dokumentiert. Möglicherweise ist die Person, die die PPS durchgeführt hat, nicht mit dem Lesen und Auswerten eines Antibiogramms vertraut gewesen. Gemäß der Leitlinie für unkomplizierte Harnwegsinfektionen ist eine Urindiagnostik bei unkomplizierter unterer Harnwegsinfektion (Zystitis) an sich nicht zu empfehlen, sollte jedoch bei älteren Personen niederschwellig erfolgen, da diese häufig keine typischen Symptome zeigen [18, 26]. In etwa einem Drittel der Fälle erfolgte eine ABA, die als Erstlinientherapie bei unkomplizierter Harnwegsinfektion empfohlen ist (Nitrofurantoin, Fosfomycin, Pivmecillinam, Nitroxolin, Trimethoprim) [18, 26]. Hierbei ist zu bedenken, dass bei Heimbewohnern aufgrund von strukturellen Veränderungen im Bereich der Harnwege oder aufgrund von Komorbiditäten vermutlich nur in wenigen Fällen von einer unkomplizierten Infektion ausgegangen werden kann.

### Strategien zum rationalen Antibiotikaeinsatz.

Strategien zum ABS wie die Verfügbarkeit von Antibiotikaverbrauchsdaten, lokale Resistenzstatistiken, Therapieleitlinien etc., die im ECDC-Protokoll abgefragt werden, sind im Gegensatz zu anderen europäischen Ländern aufgrund der in Deutschland existierenden Versorgungsstrukturen in den LTCF kaum etabliert. Die Angaben der LTCF zu dieser Thematik sind zudem nur eingeschränkt beurteilbar, da aus ihnen nicht hervorgeht, wie die Maßnahmen implementiert und umgesetzt werden.

### Schulungen.

Neben der Datenerfassung bietet das HALT-Projekt den Teilnehmenden eine intensive obligatorische Schulung, welche für IPC einschließlich Surveillance von NI und ABA sensibilisieren kann. Es wird ein einfaches und praxisnahes Instrument zur Surveillance zur Verfügung gestellt, welches auch unabhängig von Studien etabliert werden kann. Allerdings wird es, nach unseren Kenntnissen, in Deutschland bisher nur selten eingesetzt. Wünschenswert wäre ein wie in den Niederlanden und Norwegen implementiertes und unabhängig von HALT entwickeltes nationales Surveillance-Netzwerk in LTCF, welches auch die behandelnden Ärzte schult und involviert [17, 27, 28].

### Stärken und Limitationen

PPS sind weniger aufwendig als Inzidenzerhebungen, bieten aber auch nicht dieselbe Genauigkeit und geben die Prävalenz nur für einen bestimmten Zeitpunkt wieder. Eine Vergleichbarkeit zwischen den verschiedenen Erhebungen ist nur eingeschränkt möglich, da jeweils unterschiedliche Einrichtungen teilgenommen haben. Durch die Wiederholung der Erhebung ist somit lediglich eine Trendbeurteilung möglich. NI und ABA mit einer längeren Zeitdauer werden eher erfasst und schwerwiegende NI möglicherweise untererfasst, da sie oft eine Krankenhausverlegung notwendig machen.

Für die Erfassung der ABA ergibt sich eine höhere Validität der Daten als für die NI, da deren Erkennung einer komplexen Methodik bei der Erhebung der Symptome folgt, die zwar durch geschulte, aber nicht notwendigerweise erfahrene Personen durchgeführt wird. Ergebnisse vorhergehender Validierungen haben ergeben, dass Anzeichen und Symptome einer Infektion übersehen und Infektionen somit untererfasst werden [29]. Eine erneute Validierung konnte 2016 in Deutschland aufgrund organisatorischer Hindernisse aber nicht erfolgen [13].

Eine weitere Limitation stellt die nicht repräsentative Stichprobenerhebung dar. Es wurde ein „convenience sampling“ durchgeführt, da u. a. Verzeichnisse der LTCF in den Bundesländern oft nicht vollständig bzw. aktualisiert vorlagen. Entsprechend ist in der Rekrutierung ein Selektionsbias zu vermuten, was sich z. B. in der Teilnahme aus Bundesländern mit gut etablierten MRE-Netzwerken im Südwesten Deutschlands widerspiegelt. Zudem könnte es sein, dass insbesondere solche LTCF an der Studie teilgenommen haben, die sich bereits intensiver mit der Thematik beschäftigt hatten und möglicherweise in IPC kompetenter sind. Somit könnten die Ergebnisse eher eine zu niedrige Einschätzung der Prävalenz für NI und ABA bezogen auf alle LTCF in Deutschland darstellen.

## Fazit und Ausblick

Wie bereits in den Jahren 2010 und 2013 wurde auch 2016 in HALT‑3 ein zu hoher Einsatz von Chinolonen festgestellt. Dieser wirkt sich auf die Selektion von MRE in der vulnerablen Gruppe der Heimbewohner aus. Um den speziellen Herausforderungen in LTCF gerecht zu werden, wäre die Entwicklung eines auf diesen Bereich zugeschnittenen Programms zur Surveillance von NI und Verbesserung des rationalen Einsatzes von Antibiotika erforderlich. Zu berücksichtigen ist dabei die häufige individuelle medizinische Versorgung der Bewohner durch ambulante Ärzte und eine fehlende ärztliche Koordination, was die Implementierung einheitlicher Strategien zur IPC erschwert. Die Anstellung von Heimärzten könnte zu einer besseren Versorgung der älteren Menschen in LTCF beitragen, dabei wäre deren koordinierende Rolle in Bezug auf IPC erstrebenswert.

Harnwegsinfektionen wurden wie in den vorausgegangenen PPS als häufigste NI und als häufigste Indikation für eine ABA dokumentiert. Fortbildungen für Pflegepersonal zur Anwendung von Harnwegskathetern sowie der Aufbau einer einrichtungsbasierten Surveillance, zumindest für Harnwegsinfektionen (und Atemwegsinfektionen) sowie ABA, erscheinen daher sinnvoll [30]. Zusammen mit der Erstellung von Leitlinien zur Diagnostik und Therapie von Harnwegsinfektionen bei älteren Menschen könnte damit ein Beitrag zu einem rationaleren Einsatz von Antibiotika in LTCF geleistet werden. Insgesamt könnte sich somit eine Investition in den Aufbau entsprechender Infrastrukturen für Surveillance und IPC als geeignete Präventionsmaßnahme von NI sowie zur früheren Erkennung von Ausbrüchen und zu deren effektivem Management in LTCF erweisen, dies nicht nur vor dem Hintergrund der SARS-CoV-2-Pandemie, sondern ebenso angesichts der sich jährlich wiederholenden Influenzainfektionen [31]. In den meisten LTCF ist dafür neben der Stärkung der personellen Ressourcen auch der Ausbau der Digitalisierung dringend notwendig [3, 32].

## Supplementary Information









