# Supplementary material for: Healthcare-associated infections and antimicrobial use in long-term care facilities. German results of the third European point prevalence survey HALT-3
Source: Bundesgesundheitsblatt Gesundheitsforschung Gesundheitsschutz. 2022 Aug 11;65(9):863–71. [Article in German] doi: 10.1007/s00103-022-03566-3 (PMC9366828; doi:10.1007/s00103-022-03566-3)
Supplement: Supplementary file 1 [file 103_2022_3566_MOESM1_ESM.pdf]

ROBERT KOCH INSTITUT

PUNKTPRÄVALENZERHEBUNG ZUM VORKOMMEN VON NOSOKOMIALEN INFEKTIONEN UND DEM EINSATZ VON ANTIBIOTIKA IN EUROPÄISCHEN LANGZEITPFLEGEEINRICHTUNGEN (HALT-3)

IN FORTENTWICKLUNG DER 2010 DURCHGEFÜHRTEN PUNKTPRÄVALENZERHEBUNG HALT (HEALTHCARE ASSOCIATED INFECTIONS IN LONG TERM CARE FACILITIES) SOLL IM HERBST 2016 EINE WEITERE, VOM ECDC ORGANISIERTE PUNKTPRÄVALENZERHEBUNG NACH EINHEITLICHEN, EUROPÄISCH ABGESTIMMTEN UND ÜBERARBEITETEN METHODEN ERFOLGEN. ZIEL DES PROJEKTS IST LETZTLICH DIE IMPLEMENTIERUNG VON ERFASSUNGSINSTRUMENTEN ZUM VORKOMMEN VON INFEKTIONEN UND ZUM ANTIBIOTIKAEINSATZ, DIE ES DEN TEILNEHMENDEN STAATEN, ABER AUCH DEN EINRICHTUNGEN VOR ORT ERMÖGLICHEN, ENTWICKLUNGEN IM BEREICH NOSOKOMIALER INFEKTIONEN SOWIE IM BEREICH DES ANTIBIOTIKAEINSATZES ZU VERFOLGEN UND PRIORITÄRE MAßNAHMEN FÜR NATIONALE UND LOKALE INTERVENTIONEN ZU IDENTIFIZIEREN. ZUDEM KANN DAMIT AUCH DIE HÄUFIGKEIT VON INFEKTIONEN UND ANTIMIKROBIELLEN THERAPIEN IN LANGZEITPFLEGEEINRICHTUNGEN AUF EUROPÄISCHER EBENE ERKANNT UND VERGLICHEN WERDEN.

POINT PREVALENCE SURVEY OF HEALTHCARE-ASSOCIATED INFECTIONS AND ANTIMICROBIAL USE IN EUROPEAN LONG-TERM CARE FACILITIES (HALT-3)

ecdc
EUROPEAN CENTRE FOR DISEASE PREVENTION AND CONTROL

ZUSAMMENFASSUNG

- WAS: Eine Punktprävalenzstudie (PPS; Datenerhebung an einem Tag) zu pflege- und therapieassoziierten Infektionen (Healthcare-associated Infections- HAI) und zum Einsatz von Antibiotika in Langzeitpflegeeinrichtungen. Die Teilnahme an dem Projekt ist freiwillig.

- SETTING: Langzeitpflegeeinrichtungen (einschließlich Pflegeheime, Altenheime etc.)

- ZEITLICHER ABLAUF: Zwischen 1. September und 30. November 2016

- INSTRUMENTE:
  - Ein Einrichtungsfragebogen, der aggregierte Daten zu allen teilnahmeberechtigten Bewohnern und zur Einrichtung enthält.

    Teilnahmeberechtigte Bewohner: Bewohner, die Ihren Lebensmittelpunkt in der jeweiligen Einrichtung haben, d.h. dauerhaft dort leben (24/24h), am Tag der Erhebung um 8 Uhr morgens anwesend sind und nicht während der Erhebung entlassen werden.
  - Ein Bewohnerfragebogen für jeden Bewohner, der am Tag der Erhebung ein oder mehrere systemische Antibiotika erhält

    (Eingeschlossen: Antibiotika und Antimykotika zum systemischen Einsatz, Tuberkulostatika, antibiotische Inhalationstherapie;
    Ausgeschlossen: topisch bzw. lokal eingesetzte Antibiotika, Antiseptika und Virostatika)

    UND/ODER

    Beschwerden oder Symptome einer aktiven nosokomialen Infektion am Tag der Erhebung zeigt.

    (Eine Infektion ist aktiv, wenn Beschwerden und Symptome der Infektion am Erhebungstag vorhanden sind oder vorhanden waren und der Bewohner am Erhebungstag noch aufgrund dieser Infektion behandelt wird)
  - Software in die die Daten auf nationaler Ebene (durch den National Contact Point, NCP) eingegeben werden.

- ERHEBENDE PERSON: Lokal (Person aus der jeweiligen Einrichtung) oder extern (z.B. Mitarbeiter der regionalen Netzwerke, externe Hygienebeauftragte, Diplomanden, Doktoranden)
- FRIST BIS ZUR DATENÜBERMITTLUNG: 31. Dezember 2016
- SCHULUNG: Voraussetzung zur Teilnahme an der Studie ist eine vorherige Schulung, in der den Teilnehmern das Vorgehen und die Instrumente der Erhebung erläutert und anhand von Fallbeispielen geübt werden. Die Schulung wird Deutschlandweit an verschiedenen Orten durch das RKI angeboten und ist kostenfrei. Reisekosten werden anteilig übernommen.
- VALIDIERUNG: Zwei Einrichtungen in jedem teilnehmenden Staat müssen an einer Validierungsstudie (zeitgleich mit der PPS) teilnehmen, bei der die Erhebung parallel durch den NCP durchgeführt wird.

ORGANISATION UND FINANZIERUNG AUF EUROPÄISCHER EBENE:

European Centre for Disease Prevention and Control (ECDC)
Phone number: +46 (0)8 586 010 00
Fax number: +46 (0)8 586 010 01
info@ecdc.europa.eu
Tomtebodavägen 11a; 171 83 Stockholm, Sweden

HALT-3 MANAGEMENT TEAMS AUF EUROPÄISCHER EBENE:

Pete Kinross (HALT-3 Project Manager), Tommi Kärki, Carl Suetens (HAI-Net Coordinator)
Email: pete.kinross@ecdc.europa.eu / HAI-Net@ecdc.europa.eu
Tel: +46 8 5860 1478; Fax: +46 8 5860 1001
Surveillance and Response Support Unit
European Centre for Disease Prevention and Control (ECDC)
Tomtebodavägen 11A, 171 83 Stockholm, Sweden

Katrien Latour, Béatrice Jans
Email: Katrien.Latour@wiv-isp.be ; Tel: +32 2 642 57 62; Fax: +32 2 642 54 10
OD Santé Publique & Surveillance | OD Volksgezondheid & Surveillance
Rue Juliette Wytsmanstraat 14 | 1050 Brussels, Belgium
Wetenschappelijk Instituut Volksgezondheid
Institut Scientifique de Santé Publique
Scientific Institute of Public Health

ORGANISATION UND UMSETZUNG AUF NATIONALER EBENE (FÜR DEUTSCHLAND)

Dr. Claudia Ruscher
Robert Koch-Institut
Fachgebiet 14,
Angewandte Infektions-und
Krankenhaushygiene
Nordufer 20
13353 Berlin
Tel. 030/ 18754-2142
Halt-3@rki.de

Dr. Nicole Schmidt
Robert Koch-Institut
Fachgebiet 37
Nosokomiale Infektionen, Surveillance von
Antibiotikaresistenz und -verbrauch
Seestr. 10
13353 Berlin
Tel. 030/ 18754-3207
Halt-3@rki.de

INHALT

1. EINLEITUNG 4
2. ZIELE 6
3. STUDIENDESIGN 7
3.1. ZEITPLAN 7
3.2. STUDIENPOPULATION 8
3.2.1. TEILNEHMENDE LÄNDER 8
3.2.2. EINSCHLUSSKRITERIEN FÜR LANGZEITPFLEGEEINRICHTUNGEN 8
3.2.3. EINSCHLUSSKRITERIEN FÜR BEWOHNER VON LANGZEITPFLEGEEINRICHTUNGEN 9
3.3. REKRUTIERUNG VON EINRICHTUNGEN 10
3.3.1. ZAHL DER ZU REKRUTIERENDEN EINRICHTUNGEN 10
3.3.2. ERFASSENDE EINRICHTUNGEN UND PERSONEN 11
4. DATENERHEBUNG 11
4.1. EINRICHTUNGSFRAGEBOGEN (SIEHE ANHANG 1) 12
4.1.1. ABSCHNITT A - ALLGEMEINE INFORMATIONEN 12
4.1.2. ABSCHNITT B - NENNERZAHLEN 14
4.1.3. ABSCHNITT C - MEDIZINISCHE VERSORGUNG UND IHRE KOORDINATION 17
4.1.4. ABSCHNITT D – INFEKTIONSKONTROLLMAßNAHMEN 18
4.1.6. ABSCHNITT F – WIE WURDE DIE ERHEBUNG DURCHGEFÜHRT? 23
4.2. STATIONSLISTE (SIEHE ANHANG 2) 24
4.3. BEWOHNERFRAGEBOGEN (SIEHE ANHANG 3) 27
4.3.1. BEWOHNERDATEN 27
4.3.2. DATEN ZUM ANTIBIOTIKAEINSATZ 29
4.3.3. DATEN ZU INFEKTIONEN (HAI) 31
4.3.4. ISOLIERTE MIKROORGANISMEN UND ANTIBIOTIKARESISTENZ (SIEHE ANHANG 5) 44
5. DATENÜBERMITTLUNG 46
5.1. SOFTWARE 46
5.2. DATENANALYSE UND FEEDBACK 46
5.3. EIGENTUM DER DATEN 46
6. SCHULUNG 47
7. VALIDIERUNG UND ON-SITE-ASSESSMENT 47
8. ROLLE DES NATIONAL CONTACT POINT (NCP) 48
1. ETHISCHE ASPEKTE 48

9. FINANZIELLE ASPEKTE 49

KONTAKTINFORMATIONEN 50

Anhang 1. Einrichtungsfragebogen 51

Annex 2. Stationsliste *(nur für den internen Gebrauch)* 51

Anhang 3. Bewohnerfragebogen 51

Anhang 4. Definitionen für Infektionen 51

Anhang 5. Codeliste für Mikroorganismen 51

1. EINLEITUNG

Im Jahr 2008 wurde die Koordination der Surveillance von nosokomialen bzw. pflege- und therapieassoziierten Infektionen (healthcare-associated infections, HAI) auf europäischer Ebene dem European Centre for Disease Prevention and Control (ECDC) übertragen. Dort wurde daraufhin ein HAI-Surveillance Netzwerk gegründet (HAI-Net) und eine Machbarkeitsstudie zur Surveillance von HAI in europäischen Pflegeheimen durchgeführt[1]. Dieses Projekt war ein Bestandteil der Umsetzung des IPSE-Netzwerks (Improving Patient Safety in Europe), das die Verbesserung der Patientensicherheit in Europa zum Gegenstand hatte und von der Europäischen Kommission finanziert wurde. Im Dezember 2008 schließlich initiierte das ECDC das HALT-Projekt (Healthcare- associated Infections in European Long-Term Care Facilities), das in Form eines Protokolls für wiederholte Punktprävalenzstudien in Langzeitpflegeeinrichtungen eine integrierte Methodik zur Erfassung der Prävalenz von Infektionen (HAI), dem Einsatz von Antibiotika sowie zu vorhandenen Ressourcen in der Infektionsprävention und –kontrolle in diesen Einrichtungen zur Verfügung stellt.

Im Mai-September 2010 erfasste das erste HALT-Projekt Daten von 722 Einrichtungen aus insgesamt 25 europäischen Ländern[2] (72 davon aus Deutschland[3]). Hier wurde europaweit eine Prävalenz von Bewohnern mit mind. einer HAI in den teilnehmenden Einrichtungen von 2,4% festgestellt (Deutschland: 1,6%). Die Prävalenz von Bewohnern, die mind. ein systemisches Antibiotikum erhielten betrug 4,3% (Deutschland: 1,15%).

An der zweiten Erhebung im April und Mai 2013 beteiligten sich bereits über 1000 Einrichtungen (1181) aus 17 Europäischen Ländern[4] (221 aus Deutschland)[5]. In diesem Rahmen wurde bei den Bewohnern eine HAI-Prävalenz von 3,4% (Deutschland 1,7%) und eine Antibiotikaprävalenz von 4,4% (Deutschland: 1,1%) ermittelt.

Im Mai 2015 hat das ECDC die nächste Wiederholung des HALT-Projektes angestoßen und im Dezember gemeinsam mit den nationalen Kontaktpunkten die Methodik überarbeitet und diskutiert. Das vorliegende Protokoll enthält die Methodik zur Datenerfassung im Rahmen von HALT-3 im Zeitraum September-November 2016.

Ziel des Projekts ist letztlich auch die Implementierung von Erfassungsinstrumenten zum Vorkommen von Infektionen und zum Antibiotikaeinsatz, die es den teilnehmenden Staaten, aber auch den Einrichtungen vor Ort ermöglichen, Entwicklungen im Bereich nosokomialer Infektionen sowie im Bereich des Antibiotikaeinsatzes zu verfolgen und prioritäre Maßnahmen für nationale und lokale Interventionen zu erfassen. Zudem kann damit auch die Häufigkeit von Infektionen und antimikrobiellen Therapien in Langzeitpflegeeinrichtungen auf europäischer Ebene erkannt und verglichen werden.

Das Fachgebiet für angewandte Infektions- und Krankenhaushygiene am Robert Koch-Institut (FG14, Frau Dr. Claudia Ruscher) repräsentiert in dieser Erhebung den National Contact Point (NCP) für Deutschland.

---

[1] Improving Patient Safety in Europe Technical Implementation Report 2005-2008. ISPE, November 2008. Abrufbar unter: http://ecdc.europa.eu/en/healthtopics/Healthcare-associated_infections/HAI-Net/Documents/healthcare-associated-infections-IPSE-Technical-Report.pdf

[2] European Centre for Disease Prevention and Control. Point prevalence survey of healthcare-associated infections and antimicrobial use in European long-term care facilities. May–September 2010. Stockholm: ECDC; 2014. Abrufbar unter: http://ecdc.europa.eu/en/publications/_layouts/forms/Publication_DispForm.aspx?List=4f55ad51-4aed-4d32-b960-

af70113dbb90&ID=1086

[3] Wischnewski N, Mielke M, Wendt C (2011) [Healthcare-associated infections in long-term care facilities. German results of the European prevalence study HALT]. Bundesgesundheitsblatt Gesundheitsforschung Gesundheitsschutz 54(11):1147-1152

[4] European Centre for Disease Prevention and Control. Point prevalence survey of healthcare-associated infections and antimicrobial use in European long-term care facilities. April–May 2013. Stockholm: ECDC; 2014. Abrufbar unter: http://ecdc.europa.eu/en/publications/_layouts/forms/Publication_DispForm.aspx?List=4f55ad51-4aed-4d32-b960-af70113dbb90&ID=1087

[5] Ruscher C, Kraus-Haas M, Nassauer A, Mielke M (2015) [Healthcare-associated infections and antimicrobial use in long term care facilities (HALT-2): German results of the second European prevalence survey]. Bundesgesundheitsblatt Gesundheitsforschung Gesundheitsschutz 58(4-5):436-451

Tabelle 1: Verwendete Abkürzungen

Abkürzung	Bedeutung
**ECDC**	European Centre for Disease Prevention and Control
**HAI**	Healthcare associated infections, Infektionen, die mit dem Gesundheitswesen (Pflege und Therapie) assoziiert sind
**HALT**	Healthcare- associated Infections in European Long-Term Care Facilities; Europäisches Projekt unter der Organisation des ECDC, das die Häufigkeit von Infektionen (HAI) und den Einsatz von Antibiotika in Europäischen Langzeitpflegeeinrichtungen durch wiederholte Punktprävalenzstudien messen will. Erstmalig in 2010 durchgeführt.
**HALT-2**	Erste Wiederholung des Projekts im Jahr 2013
**LTCF**	Long-termcare facility; Langzeitpflegeeinrichtung
**NCP**	National Contact Point; Nationaler Kontakt beim ECDC, der das Projekt im jeweiligen Land koordiniert und mit dem ECDC kommuniziert. In Deutschland ist dies das RKI (FG14, Dr. Claudia Ruscher)
**PPS**	Point Prevalence Survey, Punktprävalenzstudie<br>Eine Erhebung, die Daten an einem definierten Tag sammelt

2. ZIELE

Die Ziele des ECDC bei der Surveillance von HAI und dem Einsatz von Antibiotika in Europäischen Langzeitpflegeeinrichtungen sind:

- Den EU-Mitgliedsländern und den Langzeitpflegeeinrichtungen ein standardisiertes Instrument zur Verfügung zu stellen, mit dessen Hilfe Entwicklungen der Prävalenz von HAI und des Antibiotikaeinsatzes verfolgt werden können;
- Prioritäten für nationale und lokale Interventionsmaßnahmen zu identifizieren und ihre Implementierung in den EU-Mitgliedsländern und den Langzeitpflegeeinrichtungen zu evaluieren;
- Die Bedeutung von HAI und Antibiotikaeinsatz in Langzeitpflegeeinrichtungen auf nationaler und europäischer Ebene einzuschätzen und zu beobachten.

Das Ziel dieses Protokolls ist die Implementierung einer Punktprävalenzerhebung zu HAI und Antibiotikaeinsatz in Langzeitpflegeeinrichtungen zu unterstützen und anzuleiten. Hauptziele des Protokolls sind:

- Die Prävalenz von HAI und die Prävalenz des Antibiotikaeinsatzes in Europäischen Langzeitpflegeinrichtungen zu identifizieren;
- Struktur- und Prozessindikatoren von Infektionsprävention und –kontrolle in diesen Einrichtungen zu erheben.

Die erhobenen Daten sind notwendig um:

- Die Prävalenz von Infektionen und Antibiotikagebrauch in Einrichtungen, Ländern und Regionen zu quantifizieren;
- Notwendigkeiten für Interventionen, Schulungen und/oder zusätzliche Ressourcen zur Infektionskontrolle zu identifizieren;
- Prioritäten für nationale und lokale Interventionen zu identifizieren und das Bewusstsein für die Thematik zu schärfen;
- Die Patientensicherheit der Bewohner von Langzeitpflegeeinrichtungen bzw. der alternden Bevölkerung im Allgemeinen zu fördern.

3. STUDIENDESIGN

3.1. ZEITPLAN

Ideal ist eine Datenerfassung in jeder Einrichtung an einem einzigen Tag. In sehr großen Einrichtungen mit einer großen Bettenzahl kann die Erfassung auch über zwei oder mehrere aufeinanderfolgende Tage aufgeteilt werden. Alle Betten einer Station sollten jedoch am selben Tag bearbeitet werden.

APRIL 2016

- Kontaktaufnahme des National Contact Point (RKI, FG14, Dr. Ruscher) mit den Moderatoren der regionalen MRE-Netzwerke und/oder zuständigen Gesundheitsämtern; Information und Motivation zur Teilnahme am Projekt.
- Rekrutierung und Information freiwillig teilnehmender Einrichtungen durch die Moderatoren der Netzwerke/zuständigen Gesundheitsämter.

APRIL-JULI 2016

- Netzwerkmoderatoren/Gesundheitsämter koppeln dem NCP zurück, welche Einrichtungen teilnehmen werden.
- Der NCP teilt jeder Einrichtung eine Einrichtungs-Studiennummer zu und erfasst die Eigenschaften der Einrichtung in einer Tabelle
- Information der Bewohner und Einholen von schriftlichen Einverständniserklärungen der Bewohner vor Ort durch die Einrichtungen (Einverständniserklärungen werden vom NCP zur Verfügung gestellt).

AUGUST-SEPTEMBER 2016

- Schulung der an der Erhebung beteiligten Pflegekräfte der teilnehmenden Einrichtungen in Ablauf und Durchführung der Erhebung durch den NCP.

SEPTEMBER-NOVEMBER 2016

- Erhebung in den Einrichtungen jeweils an einem beliebigen Tag im Zeitraum September-November Mai 2016.
- Ausfüllen der Einrichtungsfragebögen durch die erhebende Pflegekraft des jeweiligen Heims.
- Ausfüllen der Bewohnerfragebögen (nur bei Vorliegen einer Infektion und/oder systemischen Antibiotikatherapie am Stichtag).
  Hierzu wird dem jeweiligen Bewohner eine Studiennummer zugeteilt und auf dem Bewohnerfragebogen vermerkt; um dies zu vereinfachen und eine Vergabe von doppelten Nummern zu vermeiden kann eine Stationsliste verwendet werden. Die Zuordnung der Studiennummer erfolgt vor Ort durch die erhebende Pflegekraft. Die Stationsliste, die eine Zuordnung der Studiennummer zu den Klarnamen der Bewohner erlaubt, verbleibt an einem sicheren Ort in der jeweiligen Einrichtung und wird nicht an Dritte (weder an den NCP noch das ECDC oder Netzwerkmoderatoren und

Gesundheitsämter) weitergegeben. Nach Ende der Erhebung werden die Stationslisten vernichtet.

- Übermittlung der Einrichtungsfragebögen und Bewohnerfragebögen an den NCP (entweder direkt durch die Einrichtung oder vorab gesammelt durch die regionalen Netzwerke).
- Der NCP gibt die Daten der Einrichtungen unter Benutzung der zugeteilten Einrichtungsnummern/Bewohner-Studiennummern in eine vom ECDC zur Verfügung gestellte Software ein und erstellt daraus eine nationale Datenbank der deutschen Einrichtungen.

DEZEMBER 2016

- Übermittlung der nationalen Datenbank (zip-File) an das ECDC

- Im Anschluss generiert das ECDC aus allen eingegangenen Daten eine europäische Datenbank, die auf Fehler und Unvollständigkeit geprüft wird. Diese wird in das European Surveillance System (TESSy) eingespeist. TESSy sammelt, speichert und analysiert anonyme Informationen über Infektionskrankheiten in Europa.

3.2. STUDIENPOPULATION

3.2.1. TEILNEHMENDE LÄNDER

Jedes EU7/EEA-Mitgliedsland ist eingeladen, sich an der Punktprävalenzerhebung zu beteiligen. Die NCPs sind für die Einladung der Einrichtungen und die Organisation der Erhebung in ihren Staaten verantwortlich.

3.2.2. EINSCHLUSSKRITERIEN FÜR LANGZEITPFLEGEEINRICHTUNGEN

Der Begriff “Langzeitpflegeeinrichtung” umschließt die Organisation und Bereitstellung einer breiten Palette von Dienstleistung und Assistenz für Menschen, die in ihren Möglichkeiten sich unabhängig dem Alltag zu stellen eingeschränkt sind (z.B. selbständig Aktivitäten des täglichen Lebens über einen längeren Zeitraum zu bewältigen). Zudem besteht meist ein Bedarf an grundlegender medizinischer Versorgung und Pflege (z.B. Wundversorgung, Schmerzbehandlung, Medikation, Gesundheitsmonitoring, Prävention, Rehabilitation, palliative Versorgung etc.).Langzeitpflege beinhaltet eine Mischung aus pflegerischen und sozialen Komponenten und betrifft daher sowohl den medizinischen als auch den sozialen Sektor.

In Langzeitpflegeeinrichtungen leben typischerweise Menschen die,

- konstante Betreuung benötigen (24 von 24 Stunden);
- qualifizierte Pflege und nicht nur Betreuung und Assistenz bei alltäglichen Erledigungen benötigen;
- medizinisch stabil sind und keine andauernde medizinische Spezialversorgung (durch Fachärzte) benötigen;
- keine invasiven medizinischen Maßnahmen (z.B. Beatmung) benötigen.

Alle Arten von Langzeitpflegeeinrichtungen können an HALT-3 teilnehmen und bekommen im Anschluss ein individuelles Feedback ihrer Ergebnisse. Um die Vergleichbarkeit der Daten auf nationaler Ebene zu erhalten werden die Einrichtungen kategorisiert nach den häufigsten Einrichtungstypen. Auch die europäische Auswertung und Publikation der Daten erfolgt anhand dieser Kategorien.
Einrichtungen, die in keine der Kategorien fallen werden in einem eigenen Kapitel zusammengefasst.

Folgende Kategorien werden in HALT-3 unterschieden:

Tabelle 2: Kategorien von teilnehmenden Einrichtungen

KATEGORIE	BESCHREIBUNG/ DEFINITION
**Allgemeines Pflegeheim**	Bewohner dieser Einrichtungen benötigen medizinische und pflegerische Versorgung und Betreuung rund um die Uhr (24 von 24h). Typischerweise werden hier (ältere) Menschen mit schwereren Erkrankungen oder Verletzungen gepflegt.
**Altenheim/Seniorenresidenz**	Bewohner dieser Einrichtungen sind nicht in der Lage selbständig und unabhängig zu leben. Sie benötigen Betreuung und Hilfe für Aktivitäten des täglichen Lebens. Typischerweise wird persönliche Pflege, Hauswirtschaft sowie die Versorgung mit Mahlzeiten angeboten.
**Spezialisierte Einrichtungen**	Diese Einrichtungen sind auf eine bestimmte Art der Pflege spezialisiert (z.B. bestimmte chronische Erkrankungen, Multiple Sklerose, Demenz, psychiatrische Erkrankungen, Palliativpflege etc.).
**Gemischte Einrichtungen**	Diese Einrichtungen bieten verschiedenen Arten der Betreuung in derselben Einrichtung an (eine Mischung aus den oben genannten Kategorien).
**Andere Einrichtungen**	Nicht klassifizierbar anhand der oben genannten Merkmale.

Anmerkung: Diese Kategorisierung impliziert nicht, dass die Charakteristika von Bewohnern innerhalb einer Kategorie strikt homogen sein müssen.

Von der Erhebung ausgeschlossen werden Langzeitpflegestationen in Krankenhäusern sowie Wohngruppen, in denen regelhaft keine Pflege angeboten wird, betreutes Wohnen, Obdachlosenunterkünfte, Tagespflegeeinrichtungen, ambulante Pflegedienste und Wohngemeinschaften.

3.2.3. EINSCHLUSSKRITERIEN FÜR BEWOHNER VON LANGZEITPFLEGEEINRICHTUNGEN

Bewohner werden in die Erhebung eingeschlossen wenn sie

- ihren Lebensmittelpunkt in der jeweiligen Einrichtung haben, d.h. sich dort Tag und Nacht aufhalten (24/24h)

UND

- am Tag der Erhebung um 8 Uhr morgens anwesend sind

UND

- während der Erhebung nicht aus der Einrichtung entlassen werden.

Achtung: *Bewohner, die diese Kriterien erfüllen sich aber kurzzeitig außerhalb der Einrichtung befinden (für diagnostische Untersuchungen, medizinische Maßnahmen oder mit Familie und Freunden) werden trotzdem in die Erhebung eingeschlossen.*

Ausgeschlossen werden Bewohner, die

- nicht ständig in der Einrichtung leben
- zwar immer in der Einrichtung leben, am Erhebungstag um 8:00 Uhr jedoch nicht anwesend sind (z.B. durch einen Krankenhausaufenthalt oder urlaubsbedingt)
- lediglich die Tagespflege beanspruchen
- am Tag der Erhebung in ein Krankenhaus aufgenommen wurden (und mindestens eine Nacht dort bleiben)
- die Teilnahme ablehnen

Anmerkung: *Bewohner, die aufgrund einer chronischen Erkrankung regelmäßig ambulante Behandlungen in einem Krankenhaus bekommen (z.B. Hämodialyse oder Chemotherapie) sollten nicht aus der Erhebung ausgeschlossen werden, sofern sie nicht am Tag der Erhebung stationär (für mind. eine Nacht) in ein Krankenhaus aufgenommen wurden.*

3.3. REKRUTIERUNG VON EINRICHTUNGEN

Die Rekrutierung von teilnehmenden Einrichtungen erfolgt unter starker Einbindung der regionalen MRE-/MRSA-Netzwerke. Diese werden durch den NCP über die Erhebung und ihre Ziele informiert und gebeten ihrerseits Einrichtungen im jeweiligen Einzugsgebiet zu informieren und zur Teilnahme zu motivieren.

3.3.1. ZAHL DER ZU REKRUTIERENDEN EINRICHTUNGEN

Das ECDC hat eine empfohlene Mindestanzahl an Teilnehmern in jedem Land berechnet (s.u.). Für Deutschland wird eine möglichst hohe Beteiligung angestrebt, um eine breite Wahrnehmung des Themas auch im Rahmen der Tätigkeit der regionalen MRE-/MRSA-Netzwerke zu erreichen.

Country	Population >65yrs[a]	N of LTCFs	N of beds	Average LTCF size (beds)	Estimated DEFF[b]	Recommended sample size (beds)	N of LTCFs to recruit
**Germany**	17 002 915	12 354	875 549	71	2.4	3 535	50

3.3.2. ERFASSENDE EINRICHTUNGEN UND PERSONEN

Abhängig von den personellen Ressourcen können die Daten von Mitarbeitern vor Ort (beauftragter Arzt, hygienebeauftragte Pflegekräfte oder Ärzte, Pflegedienstleitung etc.) oder mithilfe der Unterstützung durch hinzugezogene Experten (durch den NCP beauftragte Personen, Mitarbeiter der regionalen Netzwerke oder Gesundheitsämter, externe Fachkräfte) erhoben werden.

Sowohl lokale als auch externe (geschulte) Fachkräfte (z.B. Mitarbeiter der regionalen MRE-Netzwerke, externe Hygienebeauftragte, die mehrere Einrichtungen betreuen, Doktoranden etc.) für die Datenerhebung besuchen die Einrichtung am Tag der Erhebung und suchen gemeinsam mit den beteiligten Pflegekräften der Einrichtungen die Bewohner auf und erfassen Beschwerden und Symptome für Infektionen, überprüfen die Datenblätter und Krankenakten sowie die dokumentierten Arzneimittelverabreichungen. Bei Bewohnern mit Beschwerden und Symptomen einer Infektion oder solchen, die am Tag der Erhebung systemische Antibiotika erhalten, werden - wenn möglich gemeinsam mit den behandelnden Ärzten – die Bewohner und die entsprechenden medizinischen Datenblätter genauer untersucht.

Es wird empfohlen, dass zusätzliches Personal am Tag der Erhebung zur Verfügung gestellt wird, um die durch die Erhebung anfallende Arbeit zu bewältigen.

Schulungsmaterial zur methodischen Einführung und zum einheitlichen Erkennen und Erfassen von Infektionen wird von Seiten des HALT-3-Management Teams erarbeitet und im Vorfeld der Erhebungen werden durch den NCP deutschlandweit Fortbildungstermine für die beteiligten Einrichtungen angeboten.

4. DATENERHEBUNG

Es werden Daten mithilfe von zwei Fragebögen erhoben:

- **Ein Einrichtungsfragebogen** (Anhang 1) dient der Erfassung wichtiger struktureller und funktioneller Parameter (Trägerschaft, Präsenz von qualifiziertem Pflegepersonal, medizinische Koordination etc.), der Häufigkeit bestimmter Charakteristika der Bewohnergesamtheit (Case Mix) sowie grundsätzliche Informationen über Regelungen zum Antibiotikaeinsatz und über Ressourcen und Strategien der Infektionskontrolle in den Einrichtungen.
- **Ein Bewohnerfragebogen** (Anhang 2) für jeden Bewohner, der am Tag der Erhebung ein systemisches Antibiotikum erhält und/oder Beschwerden und Symptome einer aktiven nosokomialen Infektion zeigt.

Eine **Stationsliste** (Anhang 3; optionaler Einsatz und nur zum internen Gebrauch in den Einrichtungen) wird zur Verfügung gestellt, um das Zusammentragen der Charakteristika der Gesamtheit der Heimbewohner zu erleichtern.

Die **Definitionen für Infektionen** (Anhang 4) sollen benutzt werden, um aktive HAI bei den Bewohnern zu identifizieren.

Eine **Liste mit Abkürzungen/Codierungen für Mikroorganismen** (Anhang 5) wird zur Vervollständigung der Angaben im Bewohnerfragebogen benötigt und ebenfalls zur Verfügung gestellt.

4.1. EINRICHTUNGSFRAGEBOGEN (SIEHE ANHANG 1)

Der Einrichtungsfragebogen sammelt Daten zu jeder teilnehmenden Einrichtung und enthält Fragen, die sich in fünf Abschnitte gliedern: A- Allgemeine Informationen, B-Nennerzahlen, C-Medizinische Versorgung und Koordination, D- Infektionskontrolle, E- Antibiotikastrategien und F-Angaben zur praktischen Durchführung der Erhebung in der Einrichtung.

Die mit diesem Fragebogen erhobenen Daten dienen insbesondere dem notwendigen Abgleichen und Differenzieren im Hinblick auf die Art der Pflege und das Vorkommen von Risikofaktoren, was zum Vergleich der Infektionsraten und der Daten zum Antibiotikaeinsatz sowohl auf regionaler bzw. nationaler Ebene als auch auf europäischer Ebene unabdingbar ist.

Es wird empfohlen, dass eine in der Einrichtung angestellte Person diesen Fragebogen beantwortet. Wenn einige Fragen nicht beantwortet werden können oder zur Beantwortung notwendige Informationen nicht verfügbar sind, sollte zusätzliche Unterstützung von verantwortlichen Personen eingeholt werden, die diese Fragen beantworten können. **Dies ist insbesondere für den Abschnitt zu Antibiotikastrategien wichtig.**

4.1.1. ABSCHNITT A - ALLGEMEINE INFORMATIONEN

Tabelle 3: Begriffserklärungen Einrichtungsfragebogen –Abschnitt A

VARIABLE	BESCHREIBUNG/ DEFINITION
**Einrichtungs-Studiennummer**	Identifizierungscode für jede Einrichtung; wird vom RKI vergeben.
**Qualifizierte Pflege, die 24 von 24 Stunden in der Einrichtung verfügbar ist**	Qualifiziertes Pflegepersonal ist Tag und Nacht in der Einrichtung verfügbar (entweder physisch präsent und/oder telefonisch bzw. über Pieper erreichbar)

**Gesamtzahl der in Vollzeit angestellten Pflegekräfte (Vollzeitäquivalente)**	Gesamtzahl der Vollzeitäquivalente ausgebildeter/ examinierter Pflegekräfte/Krankenschwestern, die in der Einrichtung angestellt sind (nicht nur am Tag der Erhebung). Geben Sie die aktuelle Situation so genau wie möglich an. Gemeint sind Pflegekräfte mit einer abgeschlossenen Berufsausbildung und staatlich anerkannten Prüfung, auch solche die nicht permanent in dieser Position in der Einrichtung angestellt sind. Auch werden hier keine Unterschiede zwischen administrativen/wissenschaftlichen oder klinischen Arbeiten der Pflegekräfte gemacht. Studenten/Auszubildende werden hier **nicht** mitgezählt.
**Gesamtzahl der in Vollzeit angestellten Pflegehelfer (Vollzeitäquivalente)**	Gesamtzahl der Vollzeitäquivalente an Pflegehelfern, die in der Einrichtung arbeiten (nicht nur am Tag der Erhebung). Geben Sie die aktuelle Situation so genau wie möglich an.<br><br>Pflegehelfer arbeiten unter der Anleitung von Pflegekräften oder Ärzten um die grundlegenden pflegerischen Tätigkeiten durchzuführen. In der Regel ist dies das Anreichen von Essen bzw. Füttern, Anziehen, Waschen und Duschen der Bewohner sowie Tätigkeiten der Körperpflege, aber auch einfache medizinische Tätigkeiten wie das Messen der Körpertemperatur oder des Blutdrucks. Auch werden keine Unterschiede zwischen administrativen/ wissenschaftlichen oder klinisch/pflegerischen Tätigkeiten der Helfer und Assistenten berücksichtigt.<br><br>Andere Fachkräfte (wie z.B. Logopäden, Physiotherapeuten etc.), logistisches Personal, Studenten, Auszubildende oder Freiwillige, die die Basisversorgung der Bewohner ohne Bezahlung durchführen werden hier **nicht** mitgezählt.
**Gesamtzahl der Bewohnerzimmer**	Summe aller Bewohnerzimmer (Einbett- und Mehrbettzimmer). Gemeinschaftsräume, Hauswirtschaftsräume etc. werden nicht mitgezählt.
**Gesamtzahl der Einzelzimmer**	Gesamtzahl aller Räume in der Einrichtung, in denen nur eine Person untergebracht werden kann. Ein Zimmer mit Doppelbett oder zwei Betten, das von Ehepaaren/Partnern bewohnt wird, zählt nicht als Einzelzimmer.
**Gesamtzahl der Einzel-/Privatzimmer mit eigenem Badezimmer**	Zahl der Einzelzimmer mit eigener Toilette und Waschgelegenheit (Waschbecken und /oder Dusche). Nur eine Toilette oder ein Toilettenstuhl ist hier nicht ausreichend und wird **nicht** mitgezählt. Zimmer, die Toiletten und Waschgelegenheiten in einem Gemeinschaftsbereich haben werden ebenfalls **nicht** mitgezählt.

Abbildung 1: HALT-3 Einrichtungsfragebogen: Abschnitt A: Allgemeine Informationen

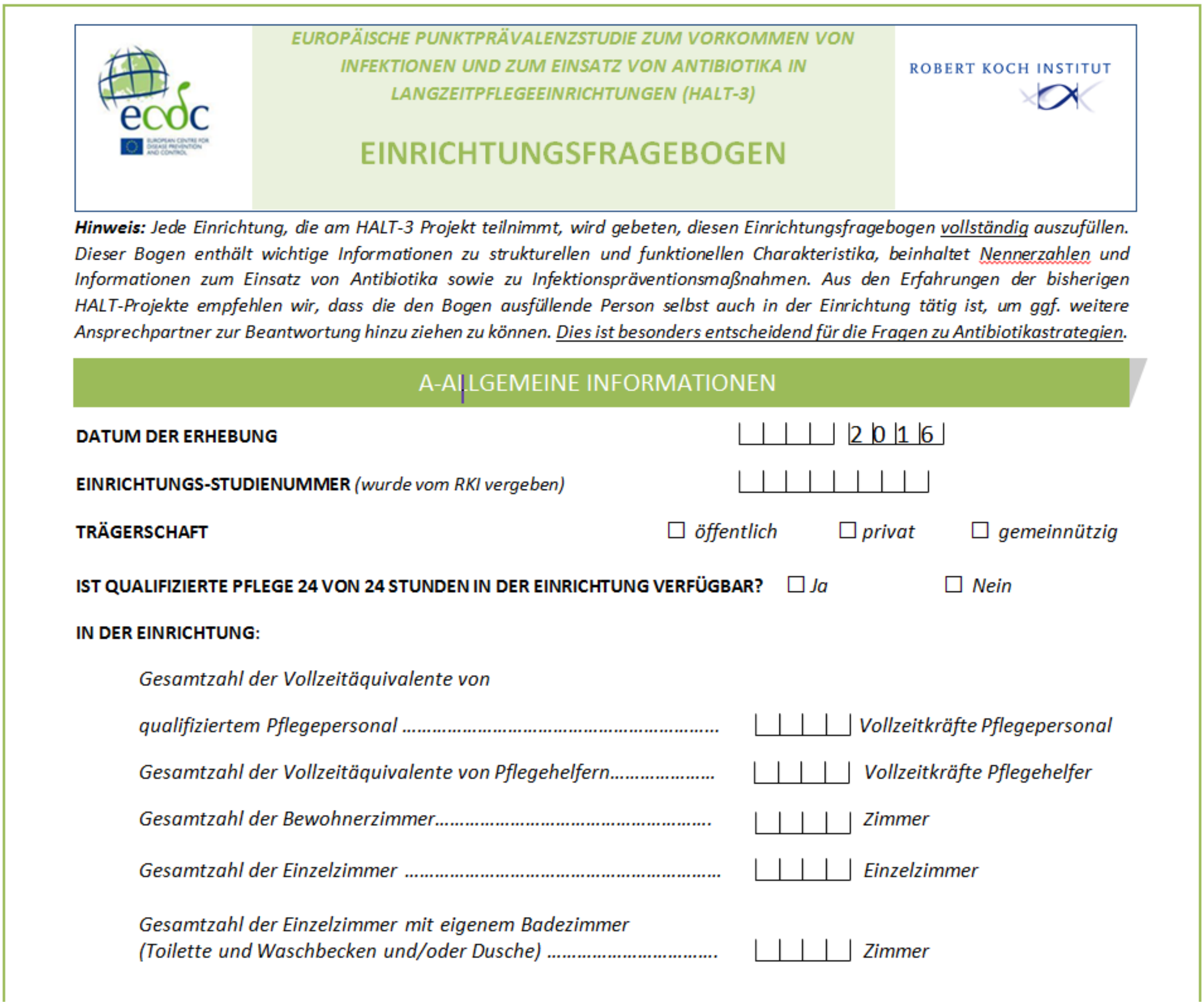

EUROPÄISCHE PUNKTPRÄVALENZSTUDIE ZUM VORKOMMEN VON INFEKTIONEN UND ZUM EINSATZ VON ANTIBIOTIKA IN LANGZEITPFLEGEEINRICHTUNGEN (HALT-3)

ROBERT KOCH INSTITUT

EINRICHTUNGSFRAGEBOGEN

Hinweis: *Jede Einrichtung, die am HALT-3 Projekt teilnimmt, wird gebeten, diesen Einrichtungsfragebogen vollständig auszufüllen. Dieser Bogen enthält wichtige Informationen zu strukturellen und funktionellen Charakteristika, beinhaltet Nennerzahlen und Informationen zum Einsatz von Antibiotika sowie zu Infektionspräventionsmaßnahmen. Aus den Erfahrungen der bisherigen HALT-Projekte empfehlen wir, dass die den Bogen ausfüllende Person selbst auch in der Einrichtung tätig ist, um ggf. weitere Ansprechpartner zur Beantwortung hinzu ziehen zu können. Dies ist besonders entscheidend für die Fragen zu Antibiotikastrategien.*

A-ALLGEMEINE INFORMATIONEN

DATUM DER ERHEBUNG |_|_|_|_| |2|0|1|6|

EINRICHTUNGS-STUDIENUMMER *(wurde vom RKI vergeben)* |_|_|_|_|_|_|_|_|

TRÄGERSCHAFT ☐ *öffentlich* ☐ *privat* ☐ *gemeinnützig*

IST QUALIFIZIERTE PFLEGE 24 VON 24 STUNDEN IN DER EINRICHTUNG VERFÜGBAR? ☐ *Ja* ☐ *Nein*

IN DER EINRICHTUNG:

Gesamtzahl der Vollzeitäquivalente von qualifiziertem Pflegepersonal .... |_|_|_|_| *Vollzeitkräfte Pflegepersonal*

Gesamtzahl der Vollzeitäquivalente von Pflegehelfern............ |_|_|_|_| *Vollzeitkräfte Pflegehelfer*

Gesamtzahl der Bewohnerzimmer............ |_|_|_|_| *Zimmer*

Gesamtzahl der Einzelzimmer .... |_|_|_|_| *Einzelzimmer*

Gesamtzahl der Einzelzimmer mit eigenem Badezimmer (Toilette und Waschbecken und/oder Dusche) .... |_|_|_|_| *Zimmer*

4.1.2. ABSCHNITT B - NENNERZAHLEN

Tabelle 4: Begriffserklärungen Einrichtungsfragebogen – Abschnitt B

VARIABLE	BESCHREIBUNG/DEFINITION
**Gesamtzahl der Betten in der Einrichtung**	Gesamtzahl der verfügbaren Betten der Einrichtung (sowohl belegt als auch frei).
**Belegte Betten**	Gesamtzahl der belegten Betten am Tag der Erhebung. Hier werden auch Betten von Bewohnern mitgezählt, die am Tag der Erhebung abwesend sind (im Krankenhaus, Arztbesuch, Urlaub, unterwegs mit Familie/Freunden etc.). Geteilte Betten von Partnern/Ehepaaren sollten als zwei Betten gezählt werden.

**In die Studie eingeschlossene Bewohner**	Gesamtzahl aller Bewohner, die am Tag der Erhebung um 8:00 Uhr anwesend sind und nicht während der Erhebung entlassen werden.
**Bewohner, die älter als 85 Jahre sind**	Gesamtzahl der Bewohner, die am Tag der Erhebung älter als 85 Jahre sind.
**Männliche Bewohner**	Gesamtzahl aller männlichen Bewohner am Tag der Erhebung.
**Bewohner mit systemischer Antibiotikatherapie**	Gesamtzahl aller Bewohner, die am Tag der Erhebung mindestens ein systemisches Antibiotikum erhalten (siehe 4.3.2.).
**Bewohner mit Beschwerden oder Symptomen einer Infektion**	Gesamtzahl aller Bewohner mit Beschwerden oder Symptomen einer Infektion (siehe 4.3.3).
**Bewohner mit Harnwegskatheter**	Gesamtzahl aller Bewohner mit Harnwegskatheter; beinhaltet jede Art harnableitender Systeme, die Urin direkt von der Blase ableiten (z.B. transurethrale Dauerkatheter, suprapubischer Blasenkatheter, Zystostomie-Katheter). Externe Katheter, die dies nicht tun (z.B. Kondom-Urinale) werden hier **nicht** mitgezählt.
**Bewohner mit Gefäßkatheter**	Gesamtzahl aller Bewohner mit Gefäßkatheter-Zugängen (venös oder arteriell) am Tag der Erhebung (periphere intravenöse Katheter, implantierte Zugänge wie Portsysteme sowie jede andere Art von intravasalen Zugängen, z.B. Dialyseshunts).
**Bewohner mit Dekubitus**	Gesamtzahl aller Bewohner, die am Tag der Erhebung einen Dekubitus aufweisen. Es sollen **alle Schweregrade** berücksichtigt werden (auch der niedrigste Grad, der nur durch nicht wegdrückbare, umschriebene Hautrötungen bei intakter Haut charakterisiert ist).
**Bewohner mit anderen Wunden**	Gesamtzahl aller Bewohner, die am Tag der Erhebung eine andere Wunde haben, einschließlich Ulcus cruris, traumatische oder chirurgische Wunden und Insertionsstellen für Gastrostomie (PEG-Sonden), Tracheostomie, Urostomie, Kolostomie oder suprapubische und peritoneale Katheter.

**Örtlich und/oder zeitlich desorientierte Bewohner**	Gesamtzahl aller Bewohner, die wiederkehrend an Verwirrtheit und Konfusion insbesondere in Bezug auf Zeit, Ort oder die Erkennung von Personen leiden (z.B. ihr Zimmer nicht finden, keine Zeitvorstellung haben, bekannte Personen nicht erkennen).
**Bewohner, die einen Rollstuhl benötigen oder bettlägerig sind**	Gesamtzahl aller Bewohner, die am Erhebungstag einen Rollstuhl zur Fortbewegung benötigen oder bettlägerig sind.
**Bewohner mit einer Operation in den zurückliegenden 30 Tagen**	Gesamtzahl aller Bewohner, die sich einer Operation innerhalb der dem Erhebungstag vorausgehenden 30 Tage unterzogen haben. Operation ist hier definiert als eine Prozedur, die eine Inzision (nicht nur Punktion mit einer Kanüle) von Haut-/Schleimhaut beinhaltet. Auch laparoskopische Eingriffe zählen dazu. Der Eingriff muss nicht zwingend in einem Operationsraum vorgenommen werden, sondern kann auch z.B. in ambulanten OP, Herzkatheterlaboren, Raum für diagnostische/therapeutische Radiologie, Endoskopie etc. stattfinden.
**Bewohner mit Inkontinenz (Harn und/oder Stuhl)**	Gesamtzahl aller Bewohner, die an Inkontinenz (Harninkontinenz und Stuhlinkontinenz) leiden und die Versorgung mit Inkontinenzmaterial während der letzten 24 Stunden vor dem Erhebungstag benötigen (am Tage und/oder während der Nacht).<br>Da dies ein Indikator für die Arbeitsbelastung des Pflegepersonals ist, sollten Bewohner mit einem harnableitenden System hier **nicht** mitgezählt werden, sondern als kontinent gezählt werden.

Abbildung 2: HALT-3 Einrichtungsfragebogen: Abschnitt B – Nennerzahlen

B-NENNERZAHLEN (CASE-MIX)

Diese Tabelle fasst, wenn sie vollständig ausgefüllt ist, die Daten aller Stationen (ggf. mithilfe der Stationslisten) für alle Bewohner der Einrichtung zusammen.

GESAMTZAHL AM TAG DER ERHEBUNG IN IHRER EINRICHTUNG:

BETTEN IN DER EINRICHTUNG *(belegte und nicht belegte Betten)*	|_|_|_|_|
BELEGTE BETTEN AM TAG DER ERHEBUNG	|_|_|_|_|
IN DIE STUDIE EINGESCHLOSSENE BEWOHNER:	
AM TAG DER ERHEBUNG (8:00 UHR) ANWESEND UND WÄHREND DER ERHEBUNG NICHT ENTLASSEN	|_|_|_|_|
BEWOHNER, DIE ÄLTER ALS 85 JAHRE SIND	|_|_|_|_|
MÄNNLICHE BEWOHNER	|_|_|_|_|
BEWOHNER MIT SYSTEMISCHER ANTIBIOTIKATHERAPIE	|_|_|_|_|
BEWOHNER MIT BESCHWERDEN ODER SYMPTOMEN EINER INFEKTION	|_|_|_|_|
BEWOHNER MIT HARNWEGSKATHETER	|_|_|_|_|
BEWOHNER MIT GEFÄßKATHETER	|_|_|_|_|
BEWOHNER MIT DEKUBITUS	|_|_|_|_|
BEWOHNER MIT ANDEREN WUNDEN	|_|_|_|_|
ÖRTLICH UND/ODER ZEITLICH DESORIENTIERTE BEWOHNER	|_|_|_|_|
BEWOHNER, DIE BETTLÄGERIG SIND ODER EINEN ROLLSTUHL BENÖTIGEN	|_|_|_|_|
BEWOHNER MIT EINER OPERATION IN DEN ZURÜCKLIEGENDEN 30 TAGEN	|_|_|_|_|
BEWOHNER MIT INKONTINENZ (HARN UND/ODER STUHL)	|_|_|_|_|

4.1.3. ABSCHNITT C - MEDIZINISCHE VERSORGUNG UND IHRE KOORDINATION

Tabelle 5: Begriffserklärungen Einrichtungsfragebogen – Abschnitt C

VARIABLE	BESCHREIBUNG/DEFINITION
**Persönlicher Hausarzt**	Vom Bewohner gewählter Hausarzt, der (auch schon vor der Aufnahme in die Einrichtung) den Bewohner seit mehreren Jahren hausärztlich betreut.
**Hausärztliche Gemeinschaftspraxis**	Verschiedene Hausärzte einer Gemeinschaftspraxis oder eines Praxis-Netzwerks, die gemeinschaftlich die medizinische Versorgung einer bestimmten Region gewährleisten.
**Medizinisches Personal, das in der Einrichtung angestellt ist**	Ärzte, die in der Einrichtung fest angestellt sind, um die medizinische Versorgung der Bewohner zu gewährleisten (Heimärzte). Dies sind nicht die persönlichen Hausärzte der Bewohner.
**Koordinierender Arzt**	Arzt, der für die Koordination medizinischer Maßnahmen und der Standardisierung von Verfahren, Maßnahmen und Strategien zuständig ist.

Abbildung 3: HALT-3 Einrichtungsfragebogen: Abschnitt C – Medizinische Versorgung und ihre Koordination

C-MEDIZINISCHE VERSORGUNG UND IHRE KOORDINATION

1. Die medizinische Versorgung, einschließlich der Verschreibung von Antibiotika, erfolgt in der Einrichtung:

☐ *Ausschließlich durch persönliche Hausärzte oder (hausärztliche) Gemeinschaftspraxen*

☐ *Ausschließlich durch in der Einrichtung angestelltes medizinisches Personal*

☐ *In Kombination aus beiden Versorgungsformen*

2. Werden die medizinischen Maßnahmen in der Einrichtung durch einen bestimmten Arzt koordiniert?

☐ *Nein, es gibt keine interne oder externe Koordination der medizinischen Maßnahmen*

☐ *Ja, ein in der Einrichtung angestellter Arzt (intern) koordiniert die medizinischen Maßnahmen*

☐ *Ja, ein nicht in der Einrichtung angestellter Arzt (extern, z.B. ein Hausarzt) koordiniert die medizinischen Maßnahmen*

☐ *Ja, sowohl ein in der Einrichtung angestellter als auch ein externer Arzt koordinieren die medizinischen Maßnahmen*

3. Können nachfolgende Personen die medizinischen Akten/ Krankenakten aller Bewohner der Einrichtung einsehen?

Der mit der medizinischen Koordination betraute Arzt der Einrichtung	☐ *Ja*	☐ *Nein*
Die Pflegekräfte	☐ *Ja*	☐ *Nein*

4.1.4. ABSCHNITT D – INFEKTIONSKONTROLLMAßNAHMEN

Tabelle 6: Begriffserklärungen Einrichtungsfragebogen – Abschnitt D

VARIABLE	BESCHREIBUNG/DEFINITION
**Infektionspräventions- und Infektionskontroll-Strategie**	Eine zusammenhängende Serie von Maßnahmen und Aktionen zur Vermeidung von Infektionen und zur Vermeidung von Erregerübertragungen in einer Population
**In Infektionsprävention und –kontrolle geschulte Personen**	Eine examinierte Pflegefachkraft oder ein Arzt, der für den Bereich der Infektionsprävention und -kontrolle zuständig ist (Erkennen und Beseitigen von Infektionsquellen, Strategien zur Vermeidung der Erregerübertragung, systematische Erfassung, Analyse und Interpretation von Infektionsdaten mit dem Ziel Präventionsmaßnahmen zu implementieren, zu evaluieren und zu verbreiten, Schulung von Pflegepersonal durch Anleitung und Informationsweitergabe zur Infektionskontrolle). Die Person kann diese Beschäftigung in Vollzeit ausüben oder in Kombination mit anderen Aufgaben wie z.B. Pflegedienstleitung oder Qualitätsbeauftragte/r.
**Hygienekommission**	Eine multidisziplinäre Kommission, der mindestens eine in Infektionsprävention und –kontrolle geschulte Person angehört (z.B. Hygienefachkraft) sowie Mitglieder der Verwaltung, der koordinierende Arzt bzw. Heimarzt (falls vorhanden), die Pflegedienstleitung. Weitere Mitglieder könnten Qualitätsbeauftragte, beratende Krankenhaushygieniker, Mikrobiologen, Chirurgen, Apotheker sowie Vertreter des gesetzlich vorgeschriebenen Heimbeirats und anderer relevanter Berufsgruppen sein.<br>Die Kommission kann in Zusammenarbeit mit Patientensicherheitsprogrammen oder Qualitätsmanagement bestehen, dennoch sollten Infektionspräventionskonzepte als klar abgrenzbares Themenfeld behandelt werden. Die Kommission soll regelmäßig tagen und Daten zur Infektionshäufigkeit/-kontrolle und entsprechende Präventionsmaßnahmen begutachten bzw. Programme, Ziele und Aktionen überwachen. Die Ergebnisse der Sitzungen sollten schriftlich festgehalten werden.
**Händedesinfektionsmittelverbrauch pro Einrichtung in Litern**	Gesamtverbrauch von alkoholischem Händedesinfektionsmittel im vergangenen Jahr (2015).
**Händehygiene-Schulung**	Schulung des Pflegepersonals und des medizinischen Personals (Altenpfleger, Pflegehelfer, Ärzte, Physiotherapeuten etc.) insbesondere neu eingestellter Mitarbeiter zur Bedeutung der Händehygiene, den Indikationen der hygienischen Händedesinfektion in Pflegeeinrichtungen und der Einreibemethode.

**Indikationen der Händedesinfektion**	Anzahl der Indikationen für eine Händedesinfektion (Momente der Händehygiene) wie sie in Kampagnen (Aktion Saubere Hände) oder Audits angegeben werden.<br>Entsprechend der WHO sollten die Indikationen der Händehygiene in Pflegeeinrichtungen mindestens die folgenden Situationen umfassen:<br>(1) VOR Bewohnerkontakt<br>(2) VOR aseptischen Tätigkeiten<br>(3) NACH Kontakt mit potentiell infektiösem Material<br>(4) NACH Bewohnerkontakt<br>(5) NACH Kontakt mit der direkten Bewohnerumgebung

Abbildung 4: HALT-3 Einrichtungsfragebogen: Abschnitt D - Infektionskontrollmaßnahmen

D-INFEKTIONSKONTROLLMMAßNAHMEN

1. Gibt es in der Einrichtung in <u>Infektionsprävention und –kontrolle geschulte</u> Personen, die dem Pflegepersonal unterstützend zur Verfügung stehen?

☐ *Ja* ☐ *Nein*

2. Wenn ja, ist/ sind diese Person/en:

☐ *Examinierte Pflegekräfte* ☐ *Ärzte* ☐ *Sowohl als auch (Arzt und Pflegekraft)*

Diese Person/en:

☐ *Arbeitet/ arbeiten in der Einrichtung (intern)*

☐ *Ist/sind nicht in der Einrichtung tätig/angestellt (extern)*

☐ *Es gibt sowohl interne als auch externe Fachkräfte*

☐

3. Sind in der Einrichtung folgende Aspekte gewährleistet?

(Bitte füllen Sie diesen Teil auch aus, wenn keine besonders geschulte Person in Ihrer Einrichtung verfügbar ist)

☐ *Schulungen und Fortbildungen zur Infektionsprävention und –kontrolle für Pflegekräfte und Pflegehelfer/innen*

☐ *Schulungen und Fortbildungen zur Infektionsprävention und –kontrolle für Haus- und medizinisches Personal*

☐ *Entwicklung von Pflegestandards*

☐ *Erfassung von Bewohnern, die mit multiresistenten Erregern kolonisiert/infiziert sind*

☐ *Benennung einer zuständigen Person für die Meldung und das Management von Ausbrüchen*

☐ *Rückmeldung von Surveillance-Ergebnissen an das pflegerische und medizinische Personal der Einrichtung*

☐ *Beaufsichtigung/ Kontrolle der Aufbereitung von Medizinprodukten und Pflegeartikeln (Desinfektion /Sterilisation)*

☐ *Entscheidung über Isolierungs- und besondere Barrieremaßnahmen für Bewohner, die mit multiresistenten Erregern besiedelt sind*

☐ *Das Angebot von jährlichen Influenzaimpfungen für alle Bewohner*

☐ *Regelmäßige Organisation, Kontrolle und Rückmeldung von Maßnahmen der Händehygiene in der Einrichtung*

☐ *Regelmäßige Organisation, Kontrolle und Rückmeldung über die Prozessüberwachung bzw. Überprüfung von Infektionspräventionsstrategien*

☐ *Keiner der oben aufgeführten Punkte*

4. Gibt es in der Einrichtung eine Hygienekommission (intern oder extern)? ☐ *Ja* ☐ *Nein*

5. Wie viele Treffen der Hygienekommission gab es im letzten Jahr?
Gesamtanzahl im letzten Jahr |_|_|_| *Treffen*

6. Kann die Einrichtung Hilfe und Expertise bei externen Hygienikern/Infektiologen formell erbitten/ einholen (z.B. Hygieniker eines lokalen Krankenhauses)?
☐ *Ja* ☐ *Nein*

7. Gibt es in der Einrichtung schriftliche Arbeitsanweisungen zu:

- dem Umgang mit MRSA und/oder anderen multiresistenten Erregern	☐ *Ja*	☐ *Nein*
- Maßnahmen der Händehygiene	☐ *Ja*	☐ *Nein*
- dem Umgang mit Harnwegskathetern	☐ *Ja*	☐ *Nein*
- dem Umgang mit Gefäßkathetern	☐ *Ja*	☐ *Nein*
- den Umgang mit Sonden/Sondenernährung	☐ *Ja*	☐ *Nein*

8. Gibt es ein Surveillancesystem zur Erfassung und Bewertung von nosokomialen Infektionen in der Einrichtung?
(z.B. jährlicher zusammenfassender Bericht zur Häufigkeit von Harnwegsinfektionen, Atemwegsinfektionen, etc…)
☐ *Ja* ☐ *Nein*

9. Welche der folgenden Produkte stehen in der Einrichtung zur Händehygiene zur Verfügung?

- Alkoholisches Händedesinfektionsmittel	☐ *Ja*	☐ *Nein*
- Alkoholhaltige Händedesinfektionstücher	☐ *Ja*	☐ *Nein*
- Flüssigseife (antiseptisch/andere)	☐ *Ja*	☐ *Nein*
- Stückseife	☐ *Ja*	☐ *Nein*

10. Welche Methode zur Händehygiene wird am häufigsten in der Einrichtung angewendet, <u>wenn die Hände nicht sichtbar verschmutzt sind</u> (nur <u>eine</u> Antwort)?
☐ *Hygienische Händedesinfektion mit alkoholhaltigem Händedesinfektionsmittel*
☐ *Händewaschen mit Wasser und Seife (nicht antiseptisch)*
☐ *Händewaschen mit Wasser und antiseptischer Seife*

11. Wie viele Liter alkoholischen Händedesinfektionsmittels wurden im letzten Jahr (2015) in der Einrichtung verbraucht?
Gesamtverbrauch in Litern |_|_|_|_| *Liter/2015*

12. Wurden im letzten Jahr Schulungen/Fortbildungen zur Händehygiene für das Personal der Einrichtung durchgeführt?
☐ *Ja* ☐ *Nein*

13. Wie viele Indikationen/Momente zur Händedesinfektion wurden im vergangenen Jahr in Ihrer Einrichtung beobachtet?
Anzahl beobachteter Indikationen |_|_|_|_| *Indikationen im letzten Jahr*

4.1.5. ABSCHNITT E – ANTIBIOTIKASTRATEGIE

Tabelle 7: Begriffserklärungen Einrichtungsfragebogen – Abschnitt E

VARIABLE	BESCHREIBUNG/ DEFINITION
**Verschreibungsliste für Antibiotika**	Eine Liste mit zugelassenen Antibiotika, die gar nicht oder nicht zur empirischen Therapie von Infektionen bei Bewohnern der Einrichtung eingesetzt werden sollen. Der Hintergrund einer solchen Liste ist es, die Wirksamkeit bestimmter Antibiotika (Reserveantibiotika) zu erhalten und diese nur bei kulturellem Nachweis und nachgewiesener Empfindlichkeit des Infektionserregers einzusetzen. In Einzelfällen kann ein schriftlich begründetes Abweichen von dieser Liste möglich sein.
**Antibiotika-Komitee**	Ein Team, das sich zumindest aus den Antibiotika-verordnenden Ärzten, einem Pharmakologen/Apotheker, sofern vorhanden dem koordinierenden Arzt/Heimarzt, einem Infektiologen und wenn möglich einem Mikrobiologen zusammensetzt. Dieses Komitee ist beauftragt lokale Richtlinien und Protokolle für den Antibiotikaeinsatz in der Einrichtung zu erarbeiten.
**Schriftliche Richtlinien für den Einsatz von Antibiotika**	Empfehlungen für die empirische und die gezielte Therapie der häufigsten Infektionen, einschließlich Dosierung, Art der Applikation und Dauer der Therapie. Meist werden Antibiotika der ersten Wahl und entsprechende Alternativen der zweiten Wahl vorgeschlagen.
**Jährlicher Verbrauch an Antibiotika**	Ein Bericht über die Menge verabreichter Antibiotika im zurückliegenden Jahr, nach Wirkstoffklassen getrennt.
**Antibiotika-Resistenz-Profile**	Nachverfolgung der Entwicklung von Antibiotikaresistenzmustern für verschiedene Mikroorganismen mit dem Ziel, die Auswahl von geeigneten Antibiotika daran zu orientieren. Diese Daten erhält man z.B. durch Surveillance der Resistenzprofile auf Befunden aus dem mikrobiologischen Labor.
**Therapierichtlinien**	Liste geeigneter Medikamente bei verschiedenen Krankheiten, als Handbuch und Hilfestellung für Ärzte bei der Verordnung von Medikamenten gedacht. Sollte zumindest ein spezifisches Kapitel zu antimikrobieller Therapie beinhalten.
**Urinteststreifen (Dipstick Test)**	Ein Urinteststreifen ist ein semiquantitativer Schnelltest zum Nachweis verschiedener Untersuchungsparameter (Protein, Blut, Leukozytenesterase, Nitrit etc.). Der imprägnierte Kunststoff- oder Papierteststreifen zeigt nach Eintauchen in die Urinprobe die Ergebnisse durch Farbumschläge an.<br>(Nicht zu verwechseln mit Dip-Slides [Eintauchprobenträger] zur diagnostischen Untersuchung von Urinproben im mikrobiologischen Labor.)

Abbildung 5: HALT-3 Einrichtungsfragebogen: Abschnitt E – Antibiotikastrategien

E-STRATEGIEN ZUM ANTIBIOTIKAEINSATZ

1. Gibt es in der Einrichtung eine Verschreibungsliste für Antibiotika, die entweder gar nicht oder nur nach Zustimmung einer verantwortlichen Person eingesetzt werden dürfen?

☐ *Ja* ☐ *Nein*

2. Wenn eine solche Liste vorhanden ist, welche Antibiotika sind nur eingeschränkt oder gar nicht zu verwenden?

☐ *Carbapeneme*

☐ *3. Generations- Cephalosporine*

☐ *Fluorochinolone*

☐ *Vancomycin*

☐ *Mupirocin*

☐ *Glycopeptide*

☐ *Breitspektrum-Antibiotika*

☐ *Intravenös verabreichte Antibiotika*

3. Welche der folgenden Strukturen gibt es in der Einrichtung?

☐ *Ein 'Antibiotika-Komitee'/eine Antibiotika-Kommission*

☐ *Regelmäßige, jährliche Fortbildungen über den angemessenen Einsatz von Antibiotika*

☐ *Schriftliche Richtlinien/Empfehlungen für den angemessenen Einsatz von Antibiotika*

☐ *Verfügbare Daten über den jährlichen Antibiotikaverbrauch, getrennt nach Wirkstoff-Gruppen*

☐ *Ein System, das medizinisches Personal an die Wichtigkeit mikrobiologischer Diagnostik zur Auswahl des geeigneten Antibiotikums hinweist bzw. erinnert*

☐ *Lokale (für Ihre Region) Resistenzprofile/Resistenzstatistiken sind der Einrichtung bzw. den verordnenden Ärzten zugänglich*

☐ *Ein System, das die Zustimmung einer verantwortlichen Person erfordert, um Reserve-Antibiotika bzw. restriktiv zu verwendende Antibiotika zu verordnen*

4. Sofern es schriftliche Therapierichtlinien in der Einrichtung gibt, befassen sie sich mit:

- *Atemwegsinfektionen?* ☐ *Ja* ☐ *Nein*
- *Harnwegsinfektionen?* ☐ *Ja* ☐ *Nein*
- *Wund- und Weichgewebeinfektionen?* ☐ *Ja* ☐ *Nein*

5. Verwenden Sie Urinteststreifen für die Erkennung von Harnwegsinfektionen?

☐ *Routinemäßig* ☐ *Manchmal* ☐ *Nie*

6. Gibt es ein System (z.B. ein Computerprogramm) zur Erfassung und Bewertung des Antibiotikaverbrauchs und zur Rückmeldung der Verbrauchsdaten in der Einrichtung ?

☐ *Ja* ☐ *Nein*

7. Gibt es ein System für die Surveillance (Erfassung und Bewertung) von multiresistenten Erregern in der Einrichtung?
(z.B. eine jährliche zusammenfassende Übersicht hinsichtlich MRSA, Clostridium difficile etc.)

☐ *Ja* ☐ *Nein*

8. Woher beziehen Sie Antibiotika in Ihrer Einrichtung? (Nur eine Antwort ist hier möglich!)

☐ *aus verschiedenen Apotheken*

☐ *aus nur einer Apotheke*

☐ *Die Einrichtung bezieht Antibiotika überhaupt nicht direkt aus Apotheken; Antibiotika werden von den Bewohnern direkt bezogen (z.B. auch durch Familien)*

10. Mit wie vielen mikrobiologischen Laboren arbeitet Ihre Einrichtung? (Nur eine Antwort ist hier möglich!)

☐ *mit mehr als einem*

☐ *mit einem einzigen*

☐ *Die Einrichtung arbeitet überhaupt nicht direkt mit mikrobiologischen Laboren zusammen; jeder Hausarzt kann mit einem Labor seiner Wahl zusammenarbeiten.*

4.1.6. ABSCHNITT F – WIE WURDE DIE ERHEBUNG DURCHGEFÜHRT?

Abbildung 6: HALT-3 Einrichtungsfragebogen: Abschnitt F – Wie wurde die Erhebung in Ihrer Einrichtung durchgeführt?

F-WIE WURDE DIE ERHEBUNG IN IHRER EINRICHTUNG DURCHGEFÜHRT?

1. Wer erfasste die HALT-2 Daten in Ihrer Einrichtung (einschl. Einrichtungs- und Bewohnerfragebogen)?

☐ *Ein Arzt*

☐ *Eine Pflegekraft*

☐ *Eine andere Person*

2. Wenn kein Arzt in die HALT-2 Datenerfassung involviert war (Einrichtungs- und Bewohnerfragebogen), wurden die Daten durch einen Arzt bestätigt?

☐ *Ja* ☐ *Nein*

4.2. STATIONSLISTE (SIEHE ANHANG 2)

Die optional zu verwendende Stationsliste kann bei der Erhebung der Nennerzahlen im Einrichtungsfragebogen hilfreich sein, da die abgefragten Kriterien dort für jede einzelne Station durch Ankreuzen einfach zusammengetragen werden können und im Anschluss nur noch addiert und in den Einrichtungsfragebogen übertragen werden müssen. Die Stationsliste ist dabei nur für den internen Gebrauch in der jeweiligen Einrichtung vorgesehen, verbleibt dort und wird am Ende der Erhebung vernichtet.

Anleitung zum Ausfüllen der Stationsliste:

- Tragen Sie alle am Tag der Erhebung anwesenden Bewohner mit der zugehörigen Zimmer/Betten-Nummer in Spalte 1 und 2 ein (dies können Sie auch bereits am Abend vor dem Erhebungstag tun)
- In der Spalte „Bewohner-Studiennummer“ können Sie die Bewohner der Station fortlaufend durchnummerieren und ein Kennkürzel für die Station davor setzen (z.B. bei der Station „Abendsonne“ ein A vor jede Ziffer: A-1, A-2 etc.)
- Kreuzen Sie in den Spalten 4 bis 15 für jeden in die Erhebung eingeschlossenen Bewohner die Faktoren an, die am Tag der Erhebung zutreffen (*in die Studie eingeschlossen: Bewohner, die in der Einrichtung leben, die am Tag der Erhebung um 8:00 Uhr anwesend sind und nicht während der Studie entlassen werden)*
- Zählen Sie am Ende der Liste für jede Spalte einzeln die Kreuze zusammen und notieren Sie die Zahl in der Übersichtstabelle am Ende der Stationsliste
- Wenn Sie für jede Station Ihrer Einrichtung eine Stationsliste ausgefüllt haben, zählen Sie die Summen der Übersichtstabellen jeder Stationsliste zusammen und tragen Sie die Ergebnisse im „Teil B- Nennerzahlen“ des Einrichtungsfragebogens ein
- Für jeden Bewohner, der am Tag der Erhebung ein Antibiotikum erhält UND/ODER Beschwerden/Symptome einer Infektion hat, muss ein Bewohnerfragebogen ausgefüllt werden (Jeder Bewohner für den in Spalte 7 und/ oder 8b ein Kreuz gemacht wurde)

Abbildung 7a: Stationsliste – Tabelle mit Risikofaktoren der Bewohner

Bitte füllen Sie diesen Teil für **alle Bewohner/innen** der Station aus				Bitte füllen Sie diesen Teil für **alle in die Studie eingeschlossenen Bewohner/innen** aus (alle, für die in Spalte 4 ein Kreuz gemacht wurde) Kreuzen Sie bitte die Felder an, in denen das Genannte für den TAG DER ERHEBUNG zutrifft												
Raum- und Bettennummer	Name des Bewohners / der Bewohnerin	Bewohner-Studiennummer	Anwesend um 08.00 Uhr und nicht entlassen während der Studie	> 85 Jahre alt	Männlich	Antibiotikatherapie	Beschwerden/ Symptome einer Infektion	Infektion erfüllt eine Definition	Harnwegskatheter	Gefäßkatheter	Dekubitus	Andere Wunden	Zeitlich und/ oder örtlich desorientiert	Bettlägerig oder es wird ein Rollstuhl benötigt	Operation in den letzten 30 Tagen	Inkontinenz (Harn und/oder Stuhl)
1	2	3	4	5	6	7	8a	8b	9	10	11a	11b	12	13	14	15

Abbildung 7b: Stationsliste – Übersichtstabelle zum Addieren aller relevanten Kriterien

NUR FÜR DEN INTERNEN GEBRAUCH! AUSGEFÜLLTE STATIONSLISTE VERBLEIBT IN DER EINRICHTUNG!

ÜBERSICHTSTABELLE: GESAMTSUMME FÜR DIESE STATION

AM TAG DER ERHEBUNG, GESAMTZAHL VON:	SPALTE	SUMME
VERFÜGBAREN BETTEN AUF DER STATION	1	
BELEGTEN BETTEN	2	
IN DIE STUDIE EINGESCHLOSSENEN BEWOHNER/INNEN	4	
Bewohner , die älter als 85 Jahre sind	5	
männliche Bewohner	6	
Bewohner unter Antibiotikatherapie	7	
Bewohner mit mind. einer Infektion	8b	
Bewohner mit Harnwegskatheter	9	
Bewohner mit Gefäßkatheter	10	
Bewohner mit Dekubitus	11a	
Bewohner mit anderen Wunden	11b	
örtlich und/ oder zeitlich desorientierte Bewohner/innen	12	
Bewohner, die bettlägerig sind oder einen Rollstuhl benötigen	13	
Bewohner, die eine Operation in den letzten 30 Tagen hatten	14	
Bewohner mit Inkontinenz (Harn und/ oder Stuhl)	15	

4.3. BEWOHNERFRAGEBOGEN (SIEHE ANHANG 3)

Ein Bewohnerfragebogen muss für jeden Bewohner ausgefüllt werden, der<br>- am Tag der Erhebung ein oder mehrere systemische Antibiotika erhält<br>**UND/ODER**<br>- Beschwerden/Symptome einer aktiven Infektion (HAI) am Tag der Erhebung zeigt

Der Bewohnerfragebogen gliedert sich in drei Teile:

- Bewohnerdaten
- Teil A: Daten zur Antibiotikatherapie
- Teil B: Daten zu Infektionen

4.3.1. BEWOHNERDATEN

Um das Risikoprofil des Bewohners zu charakterisieren müssen einige allgemeine Daten (Geburtsjahr, Alter, Aufenthaltsdauer in der Einrichtung, kürzlich zurückliegende Krankenhausaufenthalte) sowie sieben Indikatoren der Fallschwere (Case Mix Faktoren: Vorhandensein von Harnwegskathetern, Gefäßkathetern, Dekubitalulcera, Wunden, Inkontinenz, kognitiver Beeinträchtigung und eingeschränkter Mobilität) erfasst werden. Die folgende Tabelle dient als erklärendes Glossar der abgefragten Kriterien und soll das Ausfüllen des Fragebogens erleichtern.

Tabelle 8: Begriffserklärungen Bewohnerfragebogen – Bewohnerdaten

VARIABLE	BESCHREIBUNG/ DEFINITION
**Bewohnerstudiennummer**	Einmalige Codenummer, die von den Personen in der Einrichtung vergeben wird, die die Studie durchführen (siehe Stationsliste)
**Geschlecht**	Geschlecht des Bewohners: männlich oder weiblich
**Geburtsjahr**	Jahr in dem der Bewohner geboren wurde (YYYY)
**Aufenthaltsdauer in der Einrichtung**	Wie lange lebt der Bewohner bereits in dieser Einrichtung? Kürzer als ein Jahr? Oder ein Jahr und/oder länger?
**Krankenhausaufenthalt in den letzten 3 Monaten**	Wurde der Bewohner in den dem Erhebungstermin vorausgehenden 3 Monaten für mindestens eine Nacht in ein Akutkrankenhaus aufgenommen? Nur Aufenthalte in Krankenhäusern mit zumindest einer medizinischen oder chirurgischen Station sollen berücksichtigt werden.
**Operation in den letzten 30 Tagen**	Wurde der Bewohner in den letzten 30 Tagen vor der Erhebung operiert? Eine Operation ist hier definiert als eine Prozedur, die eine Inzision (nicht nur Punktion mit einer Kanüle) von Haut-/Schleimhaut beinhaltet. Auch laparoskopische Eingriffe zählen dazu. Der Eingriff muss nicht zwingend in

	einem Operationsraum vorgenommen werden, sondern kann auch z.B. im ambulanten OP, Herzkatheterlabor, Raum für diagnostische/therapeutische Radiologie, Endoskopie etc. stattfinden.
**Harnwegskatheter**	Jede Art harnableitender Systeme, die Urin direkt aus der Blase ableiten (z.B. transurethrale Dauerkatheter, suprapubische Blasenkatheter, Zystostomie-Katheter). Externe Katheter, die dies nicht tun (z.B. Kondom-Urinale) werden hier **nicht** mitgezählt.
**Gefäßkatheter**	Venöse oder arterielle Gefäßkatheter-Zugänge (periphere intravenöse Katheter, implantierte Zugänge wie Portsysteme sowie jede andere Art von intravasalen Zugängen, z.B. Dialyseshunts).
**Inkontinenz**	Verlust der Kontrolle von Blasen- und/oder Darmschließmuskel, die in einem unkontrollierten Verlust von Harn und/oder Stuhl resultiert und die Versorgung mit Inkontinenzmaterial während der letzten 24 Stunden vor dem Erhebungstag erfordert (am Tage und/oder während der Nacht). Ein Bewohner mit einem harnableitenden System sollte nicht als inkontinent, sondern als kontinent gezählt werden.
**Dekubitus**	Es sollen alle Schweregrade berücksichtigt werden, auch der niedrigste Grad, der nur durch eine umschriebene Hautrötung bei intakter Haut, die sich nicht wegdrücken lässt, charakterisiert ist.
**Andere Wunden**	Gesamtzahl aller Bewohner, die am Tag der Erhebung eine andere Wunde haben, z.B. Ulcus cruris, traumatische oder chirurgische Wunden und Insertionsstellen für Gastrostomie (PEG-Sonden), Tracheostomie, Urostomie, Kolostomie oder suprapubische und peritoneale Katheter.
**Zeitliche und örtliche Desorientiertheit**	Gesamtzahl aller Bewohner, die wiederkehrend insbesondere in Bezug auf Zeit und Ort nicht orientiert sind oder Personen nicht erkennen (z.B. ihr Zimmer nicht finden, keine Zeitvorstellung haben) oder kognitiv eingeschränkt sind.
**Mobilität**	Ist der Bewohner am Erhebungstag mobil (kann sich selbstständig mit oder ohne Unterstützung durch Gehhilfen fortbewegen), benötigt er einen Rollstuhl zur Fortbewegung oder ist er bettlägerig?

Abbildung 8: HALT-3 Bewohnerfragebogen – Bewohnerdaten

BEWOHNERDATEN		
Geschlecht	☐ Männlich	☐ Weiblich
Geburtsjahr	|_|_|_|_|	
Aufenthalt in dieser Einrichtung	☐ Kürzer als 1 Jahr	☐ 1 Jahr oder länger
Krankenhausaufenthalt in den letzten 3 Monaten	☐ Ja	☐ Nein
Operation in den letzten 30 Tagen	☐ Ja	☐ Nein
Harnwegskatheter	☐ Ja	☐ Nein
Gefäßkatheter	☐ Ja	☐ Nein
Inkontinenz (Harn und/oder Stuhl)	☐ Ja	☐ Nein
Wunden:		
○ Dekubitus	☐ Ja	☐ Nein
○ Andere Wunden	☐ Ja	☐ Nein
Zeitlich und/oder örtlich Desorientiertheit	☐ Ja	☐ Nein
Mobilität	☐Selbstständig ☐Rollstuhl ☐Bettlägerig	

4.3.2. DATEN ZUM ANTIBIOTIKAEINSATZ

Folgende Antibiotika sollen in der Erhebung im Bewohnerfragebogen berücksichtigt werden, sofern sie oral, parenteral (intravenös), intramuskulär, subkutan, inhalativ oder rektal verabreicht werden:

- Systemischen Antibiotika (ATC-Gruppe J01), systemische Antimykotika (J02) und Fungizide (D01BA)
- Intestinale Antibiotika (A07AA)
- Mittel gegen Protozoen-Erkrankungen (P01AB)
- Mittel gegen Mykobakterien (J04), sofern sie zur Behandlung von Mykobakterien (incl. Tuberkulose) oder als Reserve-Antibiotika gegen multiresistente Bakterien eingesetzt werden

Ausgeschlossen werden:

- systemische antivirale Wirkstoffe (Virostatika)
- Antibiotika zur lokalen/topischen Anwendung
- Antiseptische Behandlungen

Für jedes einzelne Antibiotikum, das der Bewohner am Tag der Erhebung erhält müssen die folgenden Informationen angegeben werden:

Tabelle 9: Begriffserklärung Bewohnerfragebogen Antibiotikaeinsatz

VARIABLE	BESCHREIBUNG/ DEFINITION
**Name des Antibiotikums**	Name des Präparats oder Generikums oder Wirkstoffname (Konvertierung zu ATC-Codes erfolgt durch NCP am RKI)
**Art der Applikation**	Verabreichungsform: oral, parenteral (intravenös (iv), intramuskulär (im), subkutan (sc) oder andere (rektal, inhalativ))
**Datum Therapieende/ Kontrolluntersuchung bekannt**	Aus der Krankenakte des Bewohners sollte klar hervorgehen bis zu welchem Zeitpunkt das Antibiotikum verabreicht werden soll (Enddatum) bzw. wann über eine Weiterführung/ein Ende der antibiotischen Behandlung vom verordnenden Arzt nochmals entschieden wird (Wiedervorstellungsdatum).
**Art der Behandlung**	
**Prophylaktische Behandlung**	Eine antibiotische Behandlung, die verordnet wurde, um das Auftreten einer Infektion zu verhindern. Der Bewohner zeigte zum Zeitpunkt, an dem die Therapie verordnet wurde, keine Beschwerden oder Symptome einer Infektion. Eine antibiotische Therapie, die verordnet wurde, um Komplikationen einer bereits bestehenden Infektion zu vermeiden, sollte nicht als prophylaktische Behandlung betrachtet werden.
**Therapeutische Behandlung**	Eine antibiotische Behandlung, die verordnet wurde, um eine bestehende Infektion zu behandeln. Zum Zeitpunkt der Verordnung zeigte der Bewohner Beschwerden oder Symptome einer Infektion. Sowohl empirische (Behandlungsbeginn bevor Ursache der Infektion z.B. Mikroorganismus bekannt ist) als auch mikrobiologisch gesicherte Behandlungen (verursachender Mikroorganismus ist bekannt) sollen berücksichtigt werden.
**Indikation**	Diagnose, die zur Verordnung des Antibiotikums veranlasst hat,

	entsprechend der Organgruppe ankreuzen oder schriftlich vermerken
**Wo wurde das Antibiotikum verordnet?**	Ort, an dem das Antibiotikum verordnet wurde: In dieser Einrichtung, im Krankenhaus oder woanders

Abbildung 9: Bewohnerfragebogen: Teil A – Daten zur antibiotischen Therapie

TEIL A: DATEN ZUR ANTIBIOTISCHEN THERAPIE

	ANTIBIOTIKUM 1	ANTIBIOTIKUM 2	ANTIBIOTIKUM 3	ANTIBIOTIKUM 4
**Name des Antibiotikums** *(Handelsname oder Wirkstoff)*	------------------	------------------	------------------	------------------
**Art der Applikation**	☐ oral<br>☐ parenteral *(i.m, i.v., s.c.)*<br>☐ andere	☐ oral<br>☐ parenteral *(i.m, i.v., s.c.)*<br>☐ andere	☐ oral<br>☐ parenteral *(i.m, i.v., s.c.)*<br>☐ andere	☐ oral<br>☐ parenteral *(i.m, i.v., s.c.)*<br>☐ andere
**Datum Therapieende/ Kontrollunter-suchung bekannt?**	☐ Nein<br>☐ Ja	☐ Nein<br>☐ Ja	☐ Nein<br>☐ Ja	☐ Nein<br>☐ Ja
**Art der Behandlung**	☐ Prophylaktisch<br>☐ Therapeutisch	☐ Prophylaktisch<br>☐ Therapeutisch	☐ Prophylaktisch<br>☐ Therapeutisch	☐ Prophylaktisch<br>☐ Therapeutisch
**Indikation der Antibiotika-therapie**	☐ *Harnwege*<br>☐ *Genitaltrakt*<br>☐ *Haut o. Wunden*<br>☐ *Atemwege*<br>☐ *Magen-/Darmtrakt*<br>☐ *Augen*<br>☐ *Ohren/Nase/Mund*<br>☐ *OP-Wunde*<br>☐ *Tuberkulose*<br>☐ *systemische Infektion*<br>☐ *Fieber unklarer Genese*<br>☐ *andere (bitte benennen)*<br>------------------	☐ *Harnwege*<br>☐ *Genitaltrakt*<br>☐ *Haut o. Wunden*<br>☐ *Atemwege*<br>☐ *Magen-/Darmtrakt*<br>☐ *Augen*<br>☐ *Ohren/Nase/Mund*<br>☐ *OP-Wunde*<br>☐ *Tuberkulose*<br>☐ *systemische Infektion*<br>☐ *Fieber unklarer Genese*<br>☐ *andere (bitte benennen)*<br>------------------	☐ *Harnwege*<br>☐ *Genitaltrakt*<br>☐ *Haut o. Wunden*<br>☐ *Atemwege*<br>☐ *Magen-/Darmtrakt*<br>☐ *Augen*<br>☐ *Ohren/Nase/Mund*<br>☐ *OP-Wunde*<br>☐ *Tuberkulose*<br>☐ *systemische Infektion*<br>☐ *Fieber unklarer Genese*<br>☐ *andere (bitte benennen)*<br>------------------	☐ *Harnwege*<br>☐ *Genitaltrakt*<br>☐ *Haut o. Wunden*<br>☐ *Atemwege*<br>☐ *Magen-/Darmtrakt*<br>☐ *Augen*<br>☐ *Ohren/Nase/Mund*<br>☐ *OP-Wunde*<br>☐ *Tuberkulose*<br>☐ *systemische Infektion*<br>☐ *Fieber unklarer Genese*<br>☐ *andere (bitte benennen)*<br>------------------
**Wo wurde das Antibiotikum verordnet?**	☐ In dieser Einrichtung<br>☐ Im Krankenhaus<br>☐ An anderer Stelle	☐ In dieser Einrichtung<br>☐ Im Krankenhaus<br>☐ An anderer Stelle	☐ In dieser Einrichtung<br>☐ Im Krankenhaus<br>☐ An anderer Stelle	☐ In dieser Einrichtung<br>☐ Im Krankenhaus<br>☐ An anderer Stelle

4.3.3. DATEN ZU INFEKTIONEN (HAI)

Die folgenden Informationen müssen für jede Infektion (HAI), die entsprechend der jeweiligen Algorithmen (siehe 4.3.3.2 und Anhang 4) identifiziert wurde, angegeben werden.

Tabelle 10: Begriffserklärungen Bewohnerfragebogen – Daten zu Infektionen (HAI)

VARIABLE	BESCHREIBUNG/ DEFINITION
**Infektionscode**	Siehe 4.3.3.1und 4.3.3.2
Wenn „andere" bitte angeben	Wenn der Infektionscode „andere" ist, geben Sie bitte hier mehr Informationen zur Art der Infektion an
**Am Tag der (Wieder-) Aufnahme in die Einrichtung bereits vorhanden gewesen**	Ja= Beschwerden/Symptome der Infektion waren bereits bei Aufnahme (oder Wiederaufnahme) in die Einrichtung vorhanden
**Datum des Beginns**	Datum des Beginns der Symptome/Beschwerden (TT/MM/JJJJ)<br>Muss nicht eingetragen werden, wenn die Symptome bereits bei (Wieder-) Aufnahme in die Einrichtung vorhanden waren. Wenn die Symptome während des Aufenthalts in der Einrichtung begonnen haben, bitte das Datum eintragen, an dem sich die ersten Symptome zeigten. Wenn dies nicht bekannt ist, tragen Sie das Datum ein, an dem eine Behandlung begonnen wurde oder die erste diagnostische Untersuchung erfolgte. Wenn keine Behandlung oder Untersuchung erfolgte, schätzen Sie wann der Beginn der Infektion in etwa war.
**Ort des Beginns**	Siehe 4.3.3.3<br>Die Infektion ist entweder assoziiert mit (1) dem Aufenthalt in dieser Einrichtung, (2) dem Aufenthalt in einer anderen Langzeitpflegeeinrichtung, (3) einem Krankenhausaufenthalt oder (4) unbekannt.

Abbildung 10: HALT-3 Bewohnerfragebogen: Teil B – Daten zu Infektionen (HAI)

		INFEKTION 1	INFEKTION 2	INFEKTION 3	INFEKTION 4
Infektionscode					
Wenn „andere" bitte angeben					
Am Tag der (Wieder-)Aufnahme in die Einrichtung bereits vorhanden gewesen		☐ Nein<br>☐ Ja	☐ Nein<br>☐ Ja	☐ Nein<br>☐ Ja	☐ Nein<br>☐ Ja
Datum des Beginns		/ /	/ /	/ /	/ /
Ort des Beginns		☐ Diese Einrichtung<br>☐ Eine andere Pflegeeinrichtung<br>☐ Krankenhaus<br>☐ unbekannt	☐ Diese Einrichtung<br>☐ Eine andere Pflegeeinrichtung<br>☐ Krankenhaus<br>☐ unbekannt	☐ Diese Einrichtung<br>☐ Eine andere Pflegeeinrichtung<br>☐ Krankenhaus<br>☐ unbekannt	☐ Diese Einrichtung<br>☐ Eine andere Pflegeeinrichtung<br>☐ Krankenhaus<br>☐ unbekannt
A: Name des Mikro-organismus<br><br>B: Getestete Antibiotika-resistenz[1]<br><br>Nur für STAAUR, ENC***, ACIBAU, PSEAER oder Enterobakterien (CIT***, ENB***, ESCCOL, KLE***, MOGSPP, PRT***, SER***)	1.A				
	B				
	2. A				
	B				
	3.A				
	B				

[1]Resistenzen, die hier berücksichtigt werden:
STAAUR: Oxacillin (OXA) oder Glycopeptide (GLY);
ENC***: nur GLY;
Enterobakterien: 3.-Generation-Cephalosporine (C3G) oder Carbapeneme (CAR);
PSEAER und ACIBAU: nur CAR.

Resistenz: S=Sensibel, I=Intermediär, R=Resistent, U=unbekannt

4.3.3.1. AKTIVE NOSOKOMIALE BZW. PFLEGE- UND THERAPIEASSOZIIERTE INFEKTIONEN (HAI)

Im Zuge der Erhebung sollen Bewohner identifiziert werden, die Beschwerden oder Symptome einer aktiven Infektion (HAI) am Tag der Erhebung zeigen. Eine aktive pflege- und therapieassoziierte Infektion (assoziiert mit dem Aufenthalt in einer medizinisch/pflegerischen Einrichtung, z.B. einer Langzeitpflegeeinrichtung oder einem Krankenhaus) ist definiert als:

A. Beschwerden und Symptome der Infektion:

- Sind am Tag der Erhebung vorhanden **UND** neu bzw. sich akut verschlechternd

  ODER
- Waren während der letzten 14 Tage vor der Erhebung vorhanden **UND** neu bzw. sich akut verschlechternd **UND** der Bewohner wird am Erhebungstag (noch) aufgrund dieser Infektion behandelt

UND

B. Der Beginn der Symptome:

- War mehr als 48 Stunden, nachdem der Bewohner in die Einrichtung aufgenommen (oder wiederaufgenommen) wurde; also an Tag 3 nach Aufnahme in die derzeitige Einrichtung

  ODER

- War vor weniger als 48 Stunden nachdem der Bewohner aus einer anderen medizinisch/pflegerischen Einrichtung (einem anderen Pflegeheim oder einem Krankenhaus) in die derzeitige Einrichtung (wieder-)aufgenommen wurde (also am Tag der Aufnahme oder an Tag 2)

  ODER

  - Tiefe postoperative Organ-/Körperhöhlen-Wundinfektion, die weniger als 90 Tage nach Implantat-Operationen auftreten

  ODER

  - Andere postoperative Wundinfektionen, die weniger als 30 Tage nach der Operation auftreten

  ODER

  - Clostridium difficile-Infektionen, die weniger als 28 Tage nach Entlassung aus einer anderen medizinisch/pflegerischen Einrichtung (einem anderen Pflegeheim oder einem Krankenhaus)

Anmerkungen:

a. Chronische Symptome wie Husten oder Harndrang, stehen häufig nicht mit einer Infektion im Zusammenhang. Nicht infektiöse Ursachen von Beschwerden und Symptomen sollten im Vorfeld der Diagnose immer abgeklärt werden. Dennoch ist die Veränderung des Gesundheitszustandes des Bewohners ein wichtiger Hinweis auf die Entwicklung einer Infektion.

b. Wenn die Symptome oder Beschwerden die Definitionen einer Infektion erfüllen, sollte diese im Bewohnerfragebogen eingetragen werden. Nach Möglichkeit sollte dann geschaut werden, ob in den vorherigen 2 Wochen auch schon Symptome in den Patientenakten vermerkt sind, oder dies sollte mit dem behandelnden Hausarzt besprochen werden.

4.3.3.2. BENUTZUNG DER DEFINITIONS-ALGORITHMEN UND IDENTIFIZIEREN DES INFEKTIONSCODES

Abbildung 11: HALT-3 Definitionen für Infektionen (Seite 1 von 6)

ecdc | EUROPÄISCHE PUNKTPRÄVALENZSTUDIE ZUM VORKOMMEN VON NOSOKOMIALEN INFEKTIONEN UND ZUM EINSATZ VON ANTIBIOTIKA IN LANGZEITPFLEGEEINRICHTUNGEN (HALT-3) | ROBERT KOCH INSTITUT

DEFINITIONEN UND ALGORITHMEN FÜR INFEKTIONEN

WICHTIGER HINWEIS: Alle am Tag der Erhebung aktiven Infektionen sollten erfasst werden. Eine Infektion ist aktiv, wenn am Tag der Erhebung Beschwerden/Symptome vorliegen ODER Beschwerden/ Symptome in der Vergangenheit vorlagen und der Bewohner am Tag der Erhebung (noch) aufgrund dieser Infektion behandelt wird. In diesem Fall sollte das Vorhandensein von Beschwerden/ Symptomen in den zwei Wochen vor der Erhebung überprüft werden (z.B. in den Bewohnerakten oder durch den Hausarzt), werden um sicher zu sein, dass die Symptome den Definitionen für die jeweilige Infektion entspricht.

* **Fieber**: 1) einmalig > 37.8°C oral/Trommelfell oder 2) wiederholt > 37.2°C oral oder > 37.5°C rektal oder 3) > 1.1°C über Ausgangstemperatur bei jeder Messmethode (oral, Trommelfell, axillär)
** **Leukozytose:** 1) Neutrophilie > 14.000 Leukozyten /mm³ oder 2) Linksverschiebung (>6% Stabkernige oder > 1500 Stabkernige/mm³)
§ **Akute Veränderung der Bewusstseinslage**: Akuter Beginn + fluktuierender Verlauf + Aufmerksamkeitsstörung UND entweder Konfusion/Verwirrtheit oder veränderte Bewusstseinslage
§§ **Akute funktionelle Verschlechterung:** Neu aufgetretene Verschlechterung der Alltagskompetenz z.B. in folgenden Bereichen: Bett-Rollstuhltransfer, Bewegung, Ankleiden, Toilettenbenutzung, persönliche Hygiene, essen (z.B. neuer Anstieg um 3 Punkte im ADL-Score bei einer Skala von 0-28) ODER erhöhte Abhängigkeit gemessen mit einem anderen Index (z.B. Barthel-Index)

HARNWEGSINFEKTIONEN

☐ BEWOHNER OHNE HARNWEGSKATHETER

BESCHWERDEN UND SYMPTOME

MINDESTENS EINES DER FOLGENDEN (①; ② ODER ③ ODER) KRITERIEN MUSS ZUTREFFEN:

- ☐ ① Akute Dysurie ODER akute Schmerzen/ Schwellung oder Druckschmerz der Hoden, Nebenhoden oder Prostata
- ☐ ② Fieber* ODER Leukozytose**
  UND
  Eins oder mehrere der folgenden Symptome:
  - ☐ Akute Schmerzen im kostovertebralen Winkel (Nierenlager)
  - ☐ Suprapubischer Schmerz/Druckschmerz
  - ☐ Makrohämaturie
  - ☐ Neu aufgetretene oder erhöhte Frequenz beim Wasserlassen
  - ☐ Neu aufgetretener oder verstärkter Harndrang
  - ☐ Neu aufgetretene oder zunehmende Inkontinenz
- ☐ ③ Zwei oder mehr Symptome (bei Fehlen von Fieber oder Leukozytose):
  - ☐ Frequenz Wasserlassen (neu oder erhöht)
  - ☐ Suprapubischer Schmerz
  - ☐ Makrohämaturie
  - ☐ Harndrang (neu oder verstärkt)
  - ☐ Inkontinenz (neu oder zunehmend)

↓

URINKULTUR

- ☐ Nicht durchgeführt, negativ oder Testergebnisse unbekannt
- ☐ Urinkultur angelegt UND:
  - ☐ mind. 10^5 KbE/ml von nicht mehr als 2 Bakterienspezies im Spontanurin
  ODER
  - ☐ mind. 10^2 KbE/ml jede Art und Zahl von Mikroorganismen im Einmalkatheterurin

☐ BEWOHNER MIT HARNWEGSKATHETER

BESCHWERDEN UND SYMPTOME

MINDESTENS EINES DER FOLGENDEN (①;②, ③ ODER ④) KRITERIEN MUSS ZUTREFFEN:

- ☐ ① Fieber, Schüttelfrost ODER neu aufgetretene Hypotonie OHNE dass eine andere Infektion vorliegt
- ☐ ② Akute Veränderung der Bewusstseinslage § ODER akute funktionelle Verschlechterung §§ OHNE dass eine andere Diagnose vorliegt UND Leukozytose**
- ☐ ③ Neu aufgetretener suprapubischer Schmerz oder Schmerzen im kostovertebralen Winkel (Nierenlager) oder Druckschmerz
- ☐ ④ Eitriger Ausfluss im Bereich des Katheters oder akuter Schmerz, Schwellung oder Druckschmerz der Hoden, Nebenhoden oder Prostata

↓

URINKULTUR

- ☐ Nicht durchgeführt, negativ oder Testergebnisse unbekannt
- ☐ Urinkultur angelegt UND:
  - ☐ mind. 10^5 KbE/ml jede Art und Zahl von Mikroorganismen aus einer Katheterprobe

↓

- ☐ Symptome UND Urinkultur positiv: ***Infektion Bestätigt (CODE: UTI-C)***
- ☐ Symptome UND Urinkultur nicht angelegt, negativ oder Ergebnis unbekannt: ***Infektion wahrscheinlich (CODE: UTI-P)***
- ☐ Infektion wird am Erhebungstag behandelt, aber es gibt keine Dokumentation über Beschwerden/Symptome *(nur bei Infektionen aus Krankenhäusern/ anderen Pflegeeinrichtungen)*: ***Infektion importiert (CODE: UTI-I)***

Durch Abgleich der Symptome und Beschwerden der Bewohner mit den in den Definitionen aufgelisteten kann erkannt werden ob genügend Symptome vorhanden sind, um eine Infektion zu bestätigen und den jeweiligen Code in Teil B des Bewohnerfragebogens einzutragen. Das gründliche und sorgfältige Suchen nach Beschwerden/Symptomen einer Infektion ist entscheidend, um Infektionen auch als solche bestätigen zu können.

Die Algorithmen, die den Definitionen der Infektionen zu Grunde liegen basieren auf klinischen Kriterien (den CDC/SHEA Falldefinitionen[1], die wiederum auf den McGeer-Kriterien für die Surveillance von Infektionen in Pflegeheimen[2] basieren).

Nur Ergebnisse von Tests /Untersuchungen, die am Erhebungstag vorliegen sollen beim Ausfüllen der Algorithmen berücksichtigt werden. Ergebnisse, die am Folgetag vorliegen werden nicht berücksichtigt. Obgleich dies evtl. zu einer leichten Unterschätzung der Zahl der Infektionen führt, wird so dennoch die Vergleichbarkeit zwischen teilnehmenden Einrichtungen und Ländern sichergestellt.

Für Bewohner, die erst kürzlich aus einer anderen Einrichtung (anderes Pflegeheim, Krankenhaus etc.) aufgenommen wurden und noch immer aufgrund einer Infektion behandelt werden (inkl. aber *nicht nur* antibiotische Behandlungen) kann es unter Umständen schwierig sein, eine vollständige Dokumentation über zurückliegende Beschwerden /Symptome aufzufinden. In solchen Fällen können ggf. die Mitarbeiter der anderen Einrichtung auf Basis der Symptome, an die sie sich erinnern können behilflich sein, die Algorithmen auszufüllen. Wenn niemand sich an die Symptome/Beschwerden (insbesondere den Zustand des Bewohners vor der Aufnahme/ Wiederaufnahme in die Einrichtung) erinnert, kann die Infektion als „importiert“ angegeben werden (Infektions-Code + „I“), ohne dass die Definitionen erfüllt sein müssen.

Europäische Langzeitpflegeeinrichtungen haben im Vergleich zu amerikanischen oder kanadischen Einrichtungen mitunter seltener Zugang zu mikrobiologischer und klinischer Labordiagnostik. Die Definitionen beruhen jedoch auf klinischen Kriterien, die in amerikanischen Einrichtungen entwickelt wurden. Im Falle von Harnwegsinfektionen hat dieser Umstand die größte klinische Bedeutung, so dass für Harnwegsinfektionen zwei Ebenen in den Algorithmus integriert wurden: eine Ebene für "wahrscheinliche" Infektion sowie eine Ebene für "bestätigte" Infektion, die die mikrobiologischen Bestätigung erfordert.

Tabelle 11: Kodierung der Infektionen zum Eintragen im Bewohnerfragebogen

Infektion	Ebene	Infections code
**Harnwegsinfektionen**	Bestätigt/ wahrscheinlich/ importiert	UTI-C / UTI-P / UTI-I
**Atemwegsinfektionen**		

[1] *Stone, N.D., et al., Surveillance definitions of infections in long-term care facilities: revisiting the McGeer criteria. Infect Control Hosp Epidemiol, 2012. 33(10): p. 965-77.*
[2] *McGeer, A., et al., Definitions of infection for surveillance in long-term care facilities. Am J Infect Control, 1991. 19(1): p. 1-7.*

• **ERkältung/Pharyngitis**	Bestätigt / Importiert	COLD-C / COLD-I
• **Influenza-like illness ('Grippe')**	Bestätigt / Importiert	FLU-C / FLU-I
• **Pneumonie**	Bestätigt / Importiert	PNEU-C / PNEU-I
• **Andere untere Atemwegsinfektion**	Bestätigt / Importiert	LRTI-C / LRTI-I
**Postoperative Wundinfektionen**		
• **oberflächliche**	Bestätigt / Importiert	SSSI-C / SSSI-I
• **tiefe**	Bestätigt / Importiert	DSSI-C / DSSI-I
• **Organ/Körperhöhle**	Bestätigt / Importiert	OSSI-C / OSSI-I
**Hautinfektionen**		
• **Haut-/Bindegewebe-/Weichgewebe-/Wundinfektion**	Bestätigt / Importiert	SKIN-C / SKIN-I
• **Scabies**	Bestätigt / Importiert	SCAB-C / SCAB-I
• **Herpes simplex oder Herpes zoster Infektion**	Bestätigt / Importiert	HERP-C / HERP-I
• **Pilzinfektion**	Bestätigt / Importiert	FUNG-C / FUNG-I
**Gastrointestinale Infektionen**		
• **Gastroenteritis**	Bestätigt / Importiert	GE-C / GE-I
• ***Clostridium difficile* Infektion**	Bestätigt / Importiert	CDI-C / CDI-I
**Infektionen von Auge, Nase, Ohr und Mund**		
• **Konjunktivitis**	Bestätigt / Importiert	CONJ-C / CONJ-I
• **Otitiden**	Bestätigt / Importiert	EAR-C / EAR-I
• **Sinusitis**	Bestätigt / Importiert	SINU-C / SINU-I
• **Orale Candidiose**	Bestätigt / Importiert	ORAL-C/ ORAL-I
**Blutstrominfektionen**	Bestätigt / Importiert	BSI-C / BSI-I
**Fieber unklarer Genese**	Bestätigt / Importiert	FUO-C / FUO-I
**Andere Infektion(en)**		OTHER

Tabelle 12: Definitionen von Grundbegriffen, die in den Definitionsalgorithmen verwendet werden

ALLGEMEINSYMPTOME	
**Fieber**	1) einmalig > 37.8°C oral/Trommelfell oder 2) wiederholt > 37.2°C oral oder > 37.5°C rektal oder 3) > 1.1°C über Ausgangstemperatur bei jeder Messmethode (oral, Trommelfell, axillär)
**Leukozytose**	1) Neutrophilie > 14.000 Leukozyten /mm^3 oder 2) Linksverschiebung (>6% Stabkernige oder > 1500 Stabkernige/mm^3)
**Akute Veränderung der Bewusstseinslage**	Akuter Beginn + fluktuierender Verlauf + Aufmerksamkeitsstörung UND entweder Konfusion/Verwirrtheit oder veränderte Bewusstseinslage
**Akute funktionelle Verschlechterung**	Neu aufgetretene Verschlechterung der Alltagskompetenz z.B. in folgenden Bereichen: Bett-Rollstuhltransfer, Bewegung, Ankleiden,

	Toilettenbenutzung, persönliche Hygiene, essen (z.B. neuer Anstieg um 3 Punkte im ADL-Score bei einer Skala von 0-28) ODER erhöhte Abhängigkeit gemessen mit einem anderen Index (z.B. Barthel-Index)
**HARNWEGSINFEKTIONEN**	
**Schmerzen im kostovertebralen Winkel (Nierenlager)**	Schmerzen in der Rückenregion, die über den Nieren liegt (zwischen 12.Rippe und Wirbelsäule)
**Suprapubischer Schmerz/ Druckschmerz**	Schmerz oder Druckempfindlichkeit oberhalb des Schambein(bogen)s
**ATEMWEGSINFEKTIONEN**	
**Obere Atemwegsinfektion**	Infektionen des Nasenrachenraums oder der Tonsillen
**Untere Atemwegsinfektion**	Infektionen der Trachea, Bronchien, Bronchiolen oder der Lunge und Alveolen (Pneumonie)
**Lymphadenopathie**	Erkrankung der Lymphknoten (Schwellung oder Vergrößerung)
**Infiltrat**	Ablagerung von Flüssigkeit (Blut, Eiter) in Geweben oder Zellen
**Sputum**	Auswurf bzw. Expektorat der Atemwegsschleimhaut der unteren Atemwege (nicht zu verwechseln mit Speichel)
**Pleuritische Brustschmerzen**	Brustschmerz während der Einatmung, der zu schneller und flacher Atmung führen kann
**POSTOPERATIVE (PO) WUNDINFEKTIONEN**	
**Oberflächliche PO Wundinfektion**	Infektion betrifft nur die Haut und das subkutane Gewebe der Inzision
**Tiefe PO Wundinfektion**	Infektion steht im Bezug zur Operation und betrifft tieferes Gewebe (Bindegewebe, Muskeln, Faszien) der Inzision
**Körperhöhlen/Organ PO Infektion**	Infektion steht im Bezug zur Operation und betrifft Körperhöhlen oder Organe, die während der Operation geöffnet oder an denen manipuliert wurde
**HAUT-/WEICHGEWEBEINFEKTIONEN**	
**Weichgewebe**	Gewebe, das andere Strukturen oder Organe umgibt und stützt (Muskeln, Sehnen, Bänder, Nerven, Blutgefäße, Fett, Bindegewebe, Faszien und Membranen)
**Makulopapulöser Ausschlag**	Exanthem (Ausschlag) gekennzeichnet durch Rötung und Knötchen über Hautniveau
**Herpes simplex**	Virusinfektion, die zu einem Exanthem (meist im Mund-Nasenbereich) mit Gruppen von flüssigkeitsgefüllten Bläschen führt, die in der Regel schnell austrocknen
**Herpes zoster**	Virusinfektion; meist schmerzhaftes bläschenförmiges Exanthem in Regionen, in denen viele sensorische Nerven vorhanden sind (Gesicht, Brust, Schultern und Hüfte); Gürtelrose
**Scabies**	Kontagiöse Hauterkrankung die mit starkem Juckreiz einhergeht, verursacht durch

	Krätzmilben
**GASTROINTESTINALE INFEKTIONEN**	
**Clostridium difficile (CD)**	*C. difficile* (grampositives, sporenbildendes Bakterium) kann eine persistierende Diarrhoe und ulzero-hämorrhagische Kolitis verursachen
**Toxisches Megacolon**	Lebensbedrohliche Komplikation, die zu einer akuten Erweiterung (Dilatation) des Dickdarms führt und Schmerzen im Abdomen, Distensionen (Gasansammlungen), Druckschmerzen, Fieber, Tachykardie sowie Schock verursachen kann
**Pseudomembranöse Colitis**	Eine antibiotika-assoziierte Diarrhoe (oft verursacht durch *C. difficile*) gekennzeichnet durch abdominale Krämpfe, blutigen Stuhl, Fieber und Diarrhoe
**INFEKTIONEN VON AUGE/NASE/MUND/OHR**	
**Konjunktivales Erythem**	Rötung der Bindehäute

4.3.3.3. DEN URSPRUNG EINER INFEKTION IDENTIFIZIEREN

Die folgenden Abbildungen sollen dabei helfen, Bewohner mit einer aktiven Infektion (HAI) zu einer der folgenden Kategorien des „Ursprungs der Infektion“ zuzuordnen.

- Eine Infektion (HAI) ist assoziiert mit der derzeitigen Einrichtung, wenn die Infektion frühestens an Tag 3 nach (Wieder-)Aufnahme in die derzeitige Einrichtung begonnen hat (der Tag der Aufnahme ist Tag 1).

- Entsprechend der Definitionen einer aktiven Infektion (HAI), sollten Infektionen, die an Tag 1 oder 2 (2 Kalendertage) begonnen haben, ausgeschlossen werden, wenn der Bewohner nach einem privaten Aufenthalt (wieder-)aufgenommen wird.

- Eine postoperative Wundinfektion ist mit einem Krankenhausaufenthalt assoziiert, wenn die Infektion innerhalb von 30 Tagen nach der OP auftritt (wenn kein Implantat eingesetzt wurde) oder innerhalb von 90 Tagen für tiefe und Organ/Körperhöhlen-Infektionen, sowie für Operationen nach denen ein Implantat im Körper verbleibt.

- Clostridium difficile Infektionen (CDI) sind mit einem Krankenhausaufenthalt oder dem Aufenthalt in einer anderen Einrichtung assoziiert, wenn der Beginn der Symptome innerhalb von 28 Tagen nach (Wieder-)Aufnahme in die derzeitige Einrichtung liegt.

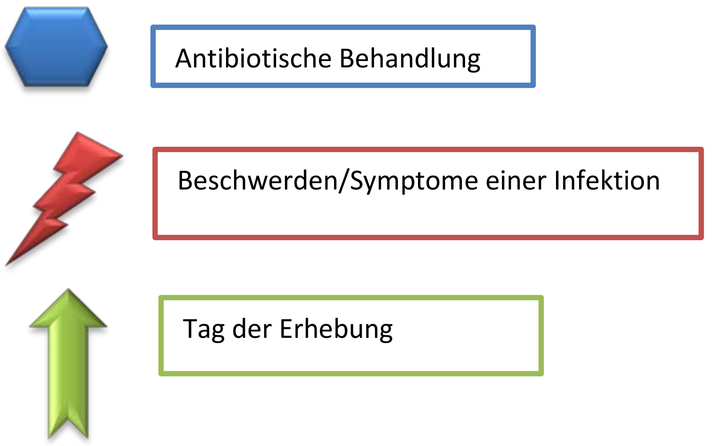

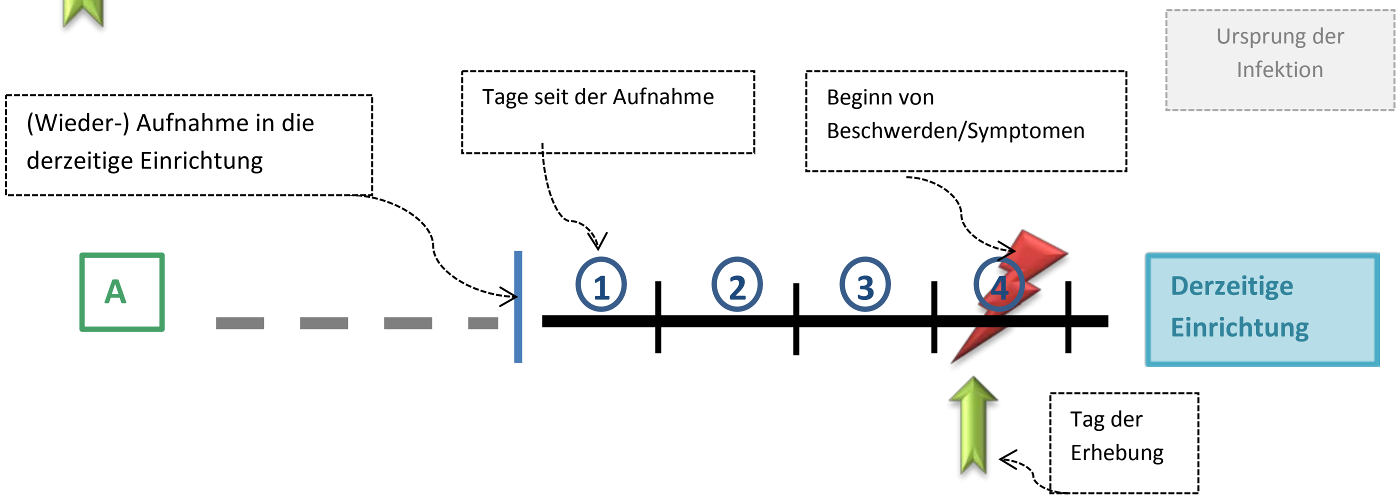

Beginn der Beschwerden/Symptome am Tag der Erhebung

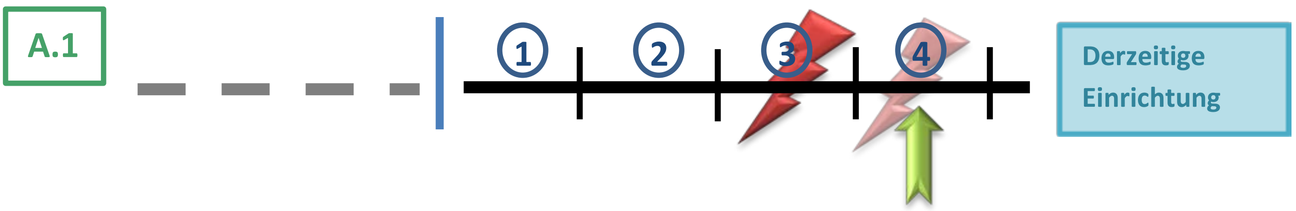

Beschwerden/Symptome am Tag der Erhebung vorhanden

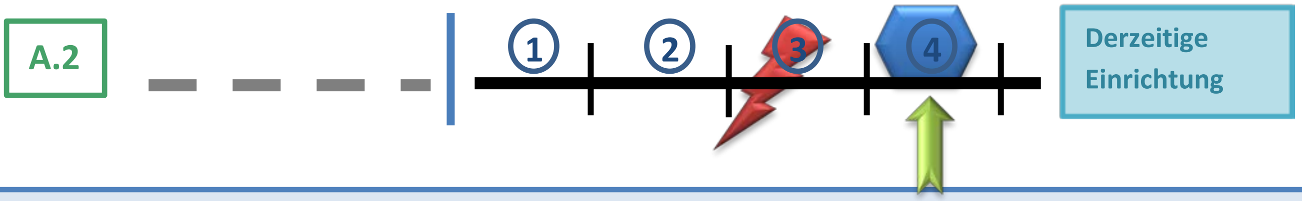

Beschwerden/Symptome am Tag der Erhebung nicht mehr vorhanden, aber der Bewohner erhält aufgrund dieser Infektion noch eine antibiotische Behandlung

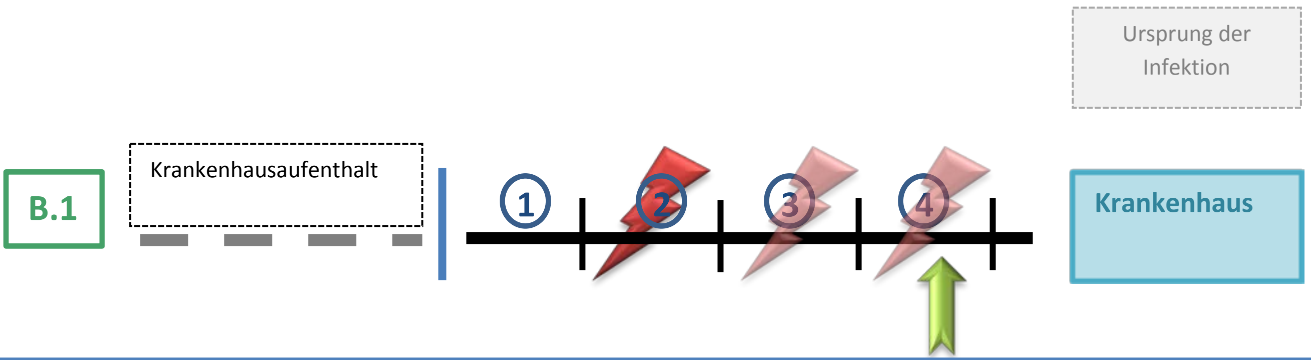

Beginn der Beschwerden/Symptome am trat innerhalb von zwei Kalendertagen nach (Wieder-)Aufnahme aus einem Krankenhaus in die Einrichtung auf

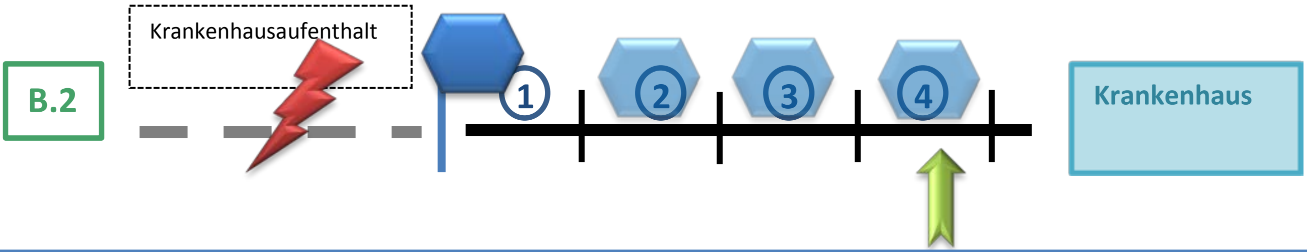

Beginn der Beschwerden/Symptome trat schon vor Entlassung aus dem Krankenhaus auf aber der Bewohner ist immer noch unter antibiotischer Behandlung bei (Wieder-)Aufnahme in die Einrichtung

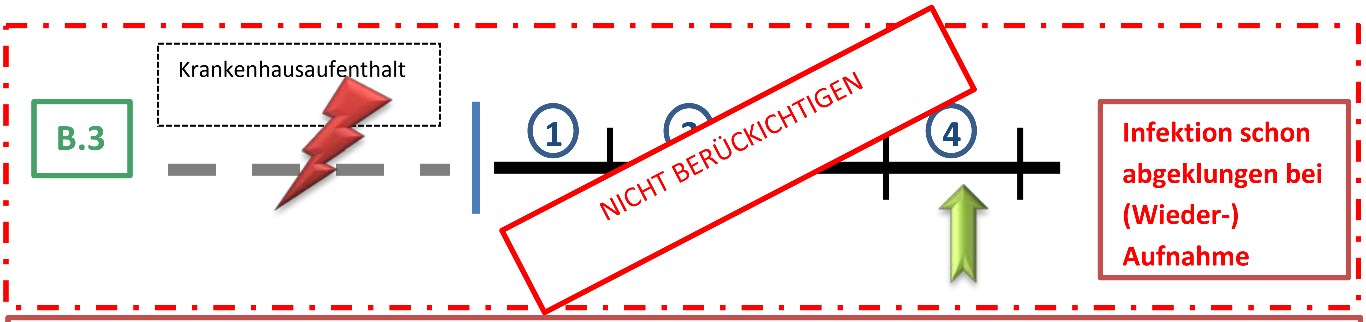

Beginn der Beschwerden/Symptome war im Krankenhaus vor Entlassung, aber die Infektion ist bei (Wieder-)Aufnahme in die Einrichtung schon abgeklungen und es besteht keine antibiotische Behandlung

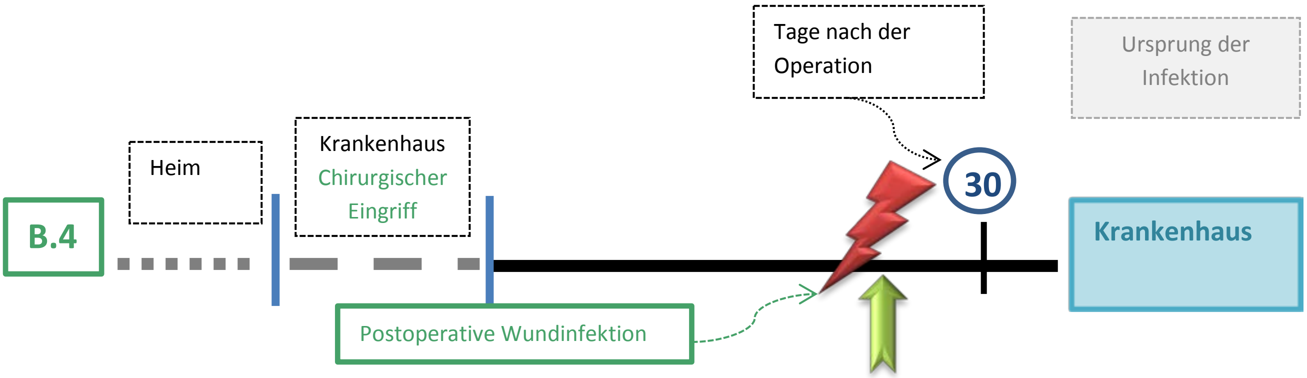

Beginn der Beschwerden/Symptome einer postoperativen Wundinfektion traten innerhalb von 30 Tagen nach einer Operation auf bei der kein Implantat im Körper verblieb

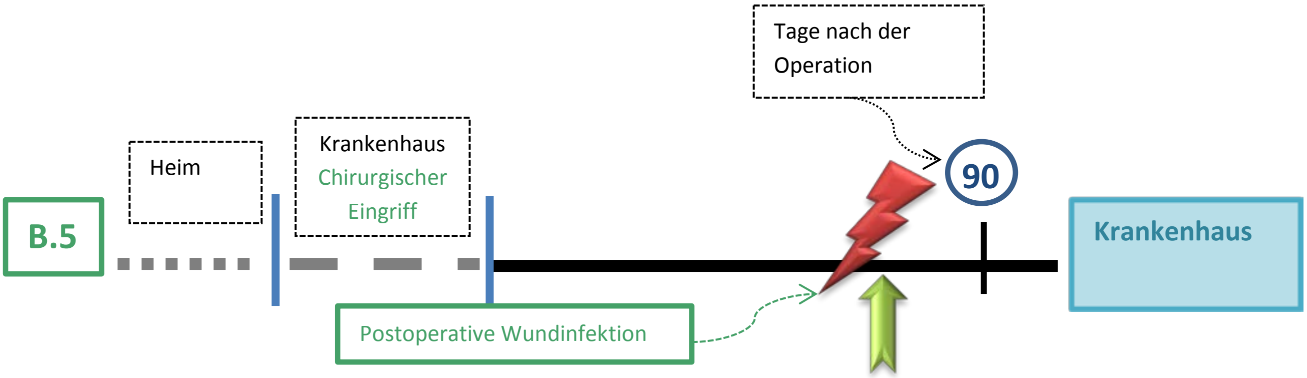

Beginn der Beschwerden/Symptome einer postoperativen Wundinfektion traten innerhalb von 90 Tagen nach einer Operation auf bei der ein Implantat im Körper verblieb

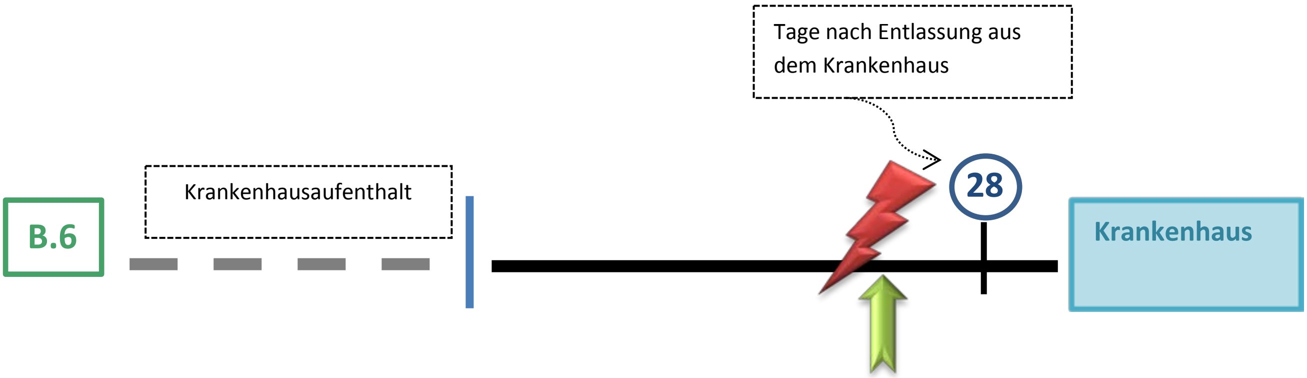

Wenn der Beginn von Beschwerden/Symptomen einer *C. difficile* Infektion innerhalb von 28 Tagen nach (Wieder-)Aufnahme aus einem Krankenhaus auftritt, sollte die Infektion als Krankenhaus-assoziiert gewertet werden.

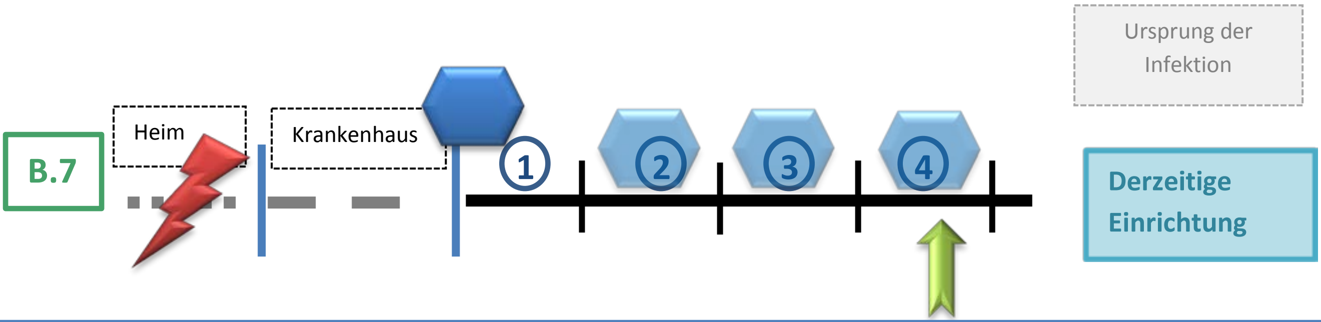

Beginn der Beschwerden/Symptome trat in der derzeitigen Einrichtung vor einem Krankenhausaufenthalt auf. Der Bewohner ist wieder in der Einrichtung aufgenommen, erhält aber noch eine antibiotische Behandlung am Tag der Erhebung.

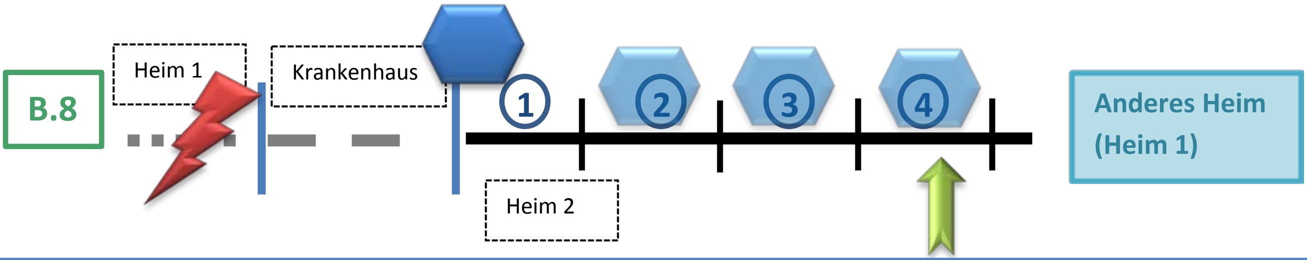

Beginn der Beschwerden/Symptome trat in einer anderen Einrichtung vor einem Krankenhausaufenthalt auf. Der Bewohner ist nach der Entlassung aus dem Krankenhaus in der derzeitigen Einrichtung aufgenommen, erhält aber noch eine antibiotische Behandlung am Tag der Erhebung.

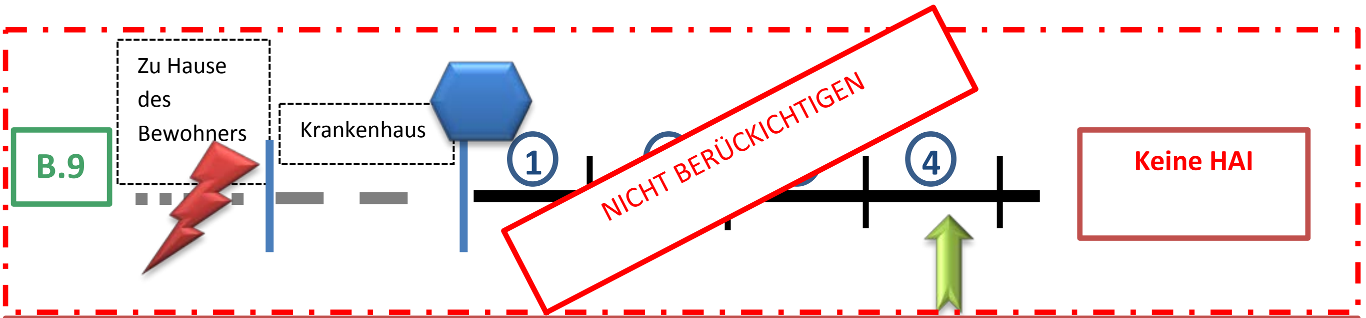

Beginn der Beschwerden/Symptome trat nicht in einer Einrichtung des Gesundheitswesens auf (sondern im häuslichen Umfeld des Bewohners)

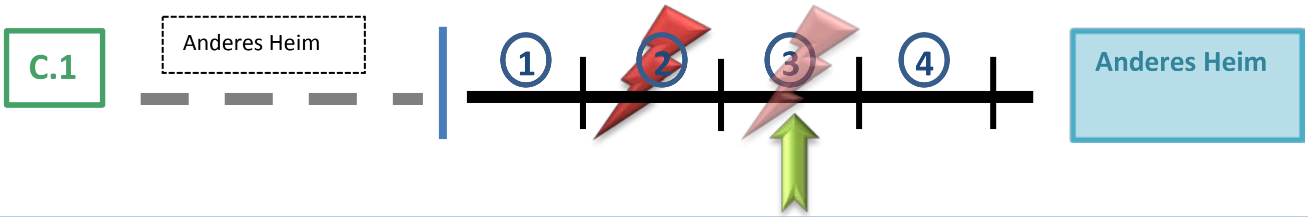

Beginn der Beschwerden/Symptom trat innerhalb von 48 nach Aufnahme aus einer anderen Pflegeeinrichtung in die derzeitige Einrichtung auf

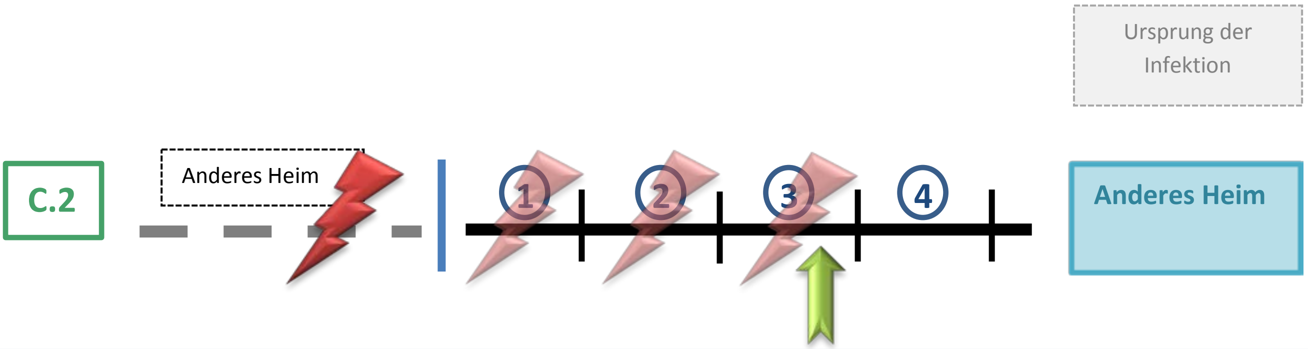

Beginn der Beschwerden/Symptome trat in einer anderen Pflegeeinrichtung auf und die Infektion ist bei Aufnahme in die derzeitige Einrichtung (und am Erhebungstag) noch aktiv.

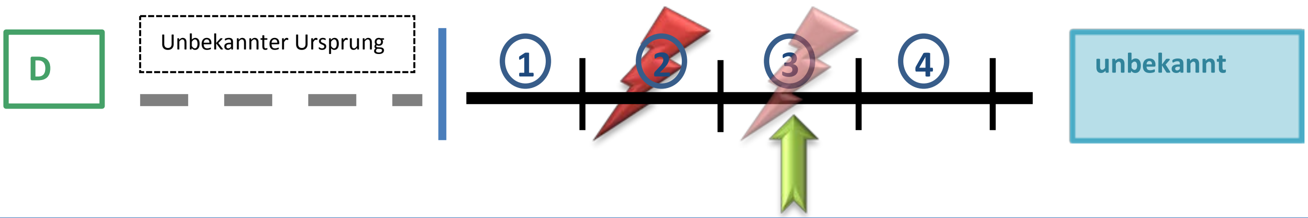

Beginn der Beschwerden/Symptome trat innerhalb von 48 Stunden nach Aufnahme in die derzeitige Einrichtung auf. Es ist sichergestellt, dass die Infektion einen Zusammenhang zum Gesundheitswesen hat (nicht in der Häuslichkeit erworben wurde).

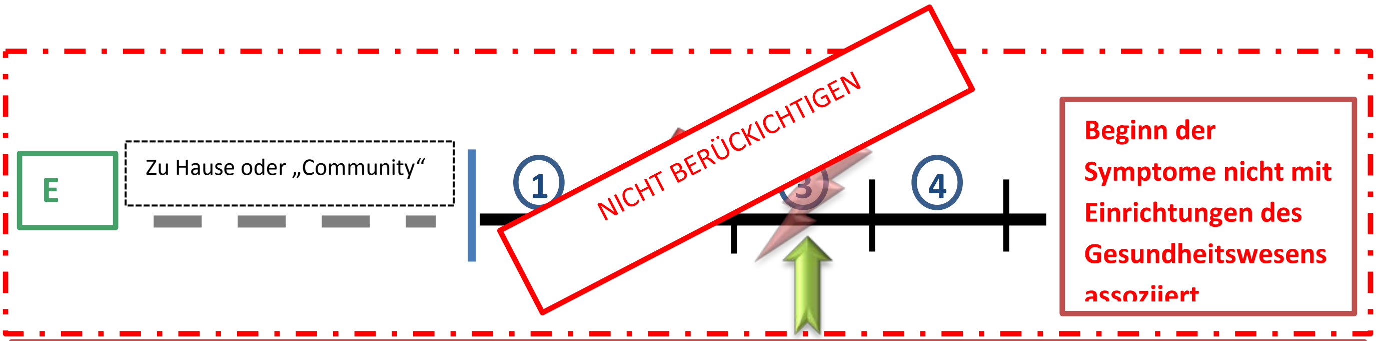

Beginn der Beschwerden/Symptome trat innerhalb von 48 nach Aufnahme aus der Häuslichkeit o.ä. in die derzeitige Einrichtung auf

4.3.4. ISOLIERTE MIKROORGANISMEN UND ANTIBIOTIKARESISTENZ (SIEHE ANHANG 5)

Daten zu isolierten Mikroorganismen und antimikrobieller Resistenz werden in Teil B- Daten zu Infektionen (HAI) des Bewohnerfragebogens erfasst. Dabei ist bekannt, dass die Häufigkeit mikrobiologischer Untersuchungen von klinischem Material in europäischen Heimen eher gering ist und auch Unterschiede in der Antibiotikaresistenztestung zwischen verschiedenen Laboren bestehen können.

Erfassen Sie nur mikrobiologische Daten, die am Erhebungstag verfügbar sind (warten Sie nicht auf Ergebnisse, die evtl. erst am Folgetag bereitstehen).

Sofern eine mikrobiologische Untersuchung durchgeführt wurde, sollten die drei wichtigsten isolierten Mikroorganismen mithilfe der Codes aus der Codierungsliste (Anhang 5) genannt werden; bzw. eine der folgenden Optionen sollte angegeben werden:

Tabelle 12: Kodierung zur Angabe von mikrobiologischen Befunden

➢ **_NOEXA**	(EXAMINATION NOT DONE)	Keine diagnostische Probe entnommen, keine mikrobiologische Untersuchung durchgeführt
➢ **_NA**	(RESULTS NOT AVAILABLE)	Ergebnisse (noch) nicht vorhanden oder nicht zugänglich; können nicht gefunden werden
➢ **_NONID**	(MICROORGANISM NOT IDENTIFIED)	Es wurde eine mikrobiologische Untersuchung durchgeführt, aber der Mikroorganismus konnte nicht korrekt identifiziert werden
➢ **_STERI**	(STERILE EXAMINATION)	Es wurde eine Untersuchung durchgeführt, aber das Ergebnis war negativ (kein Erregernachweis)

Für fünf Gruppen ausgewählter Bakterien (in der Codierungsliste rot hervorgehoben) sollten auch die Ergebnisse einer Resistenztestung wie folgt angegeben werden:

Tabelle 12: Antimikrobielle Resistenzmarker und Abkürzungen/Codes

MIKROORGANISMUS	GETESTETES ANTIBIOTIKUM	ANTIMIKROBIELLE RESISTENZ			
Staphylococcus aureus* *(STAUUR)	Oxacillin (OXA)	Sensibel (S)	/	Resistent (R)	Unbekannt (U)
	Glykopeptide (GLY)	Sensibel (S)	Intermediär (I)	Resistent (R)	Unbekannt (U)
Enterococcus spp.* *(ENC)***	Glykopeptide (GLY)	Sensibel (S)	Intermediär (I)	Resistent (R)	Unbekannt (U)
**Enterobacteriaceae[1], incl.** *Escherichia coli (ESCCOL)* *Klebsiella species (KLE***)* *Enterobacter species (ENB***)* *Proteus species (PRT***)* *Citrobacter species (CIT***)* *Serratia species (SER***)* *Morganella species (MOGSPP)*	3. Generations-Cephalosporine (C3G)	Sensibel (S)	Intermediär (I)	Resistent (R)	Unbekannt (U)
	Carbapeneme (CAR)	Sensibel (S)	Intermediär (I)	Resistent (R)	Unbekannt (U)
Pseudomonas aeruginosa *(PSEAER)*	Carbapeneme (CAR)	Sensibel (S)	Intermediär (I)	Resistent (R)	Unbekannt (U)
Acinetobacter baumannii *(ACIBAU)*	Carbapeneme (CAR)	Sensibel (S)	Intermediär (I)	Resistent (R)	Unbekannt (U)

[1] Für andere Enterobacteriaceae (*Hafnia spp., Salmonella spp., Shigella spp., Yersinia spp.*) werden keine Resistenzdaten erfasst

OXA: Empfindlichkeit gegenüber Oxacillin, oder anderen Resistenzmarkern für Methicillin-resistente *S. aureus* (MRSA) wie Cefoxitin, Cloxacillin, Dicloxacillin, Flucloxacillin, Methicillin

GLY: Empfindlichkeit gegenüber Glykopeptiden (Vancomycin, Teicoplanin)

C3G: Empfindlichkeit gegenüber 3.Generation-Cephalosporinen: Cefotaxim, Ceftriaxon,Ceftazidim

CAR: Empfindlichkeit gegenüber Carbapenemen: Imipenem, Meropenem, Doripenem

Abbildung 12: HALT-3-Bewohnerfragebogen: Teil B-Daten zu Infektionen (HAI) – Daten zu isolierten Mikroorganismen und antimikrobieller Resistenz

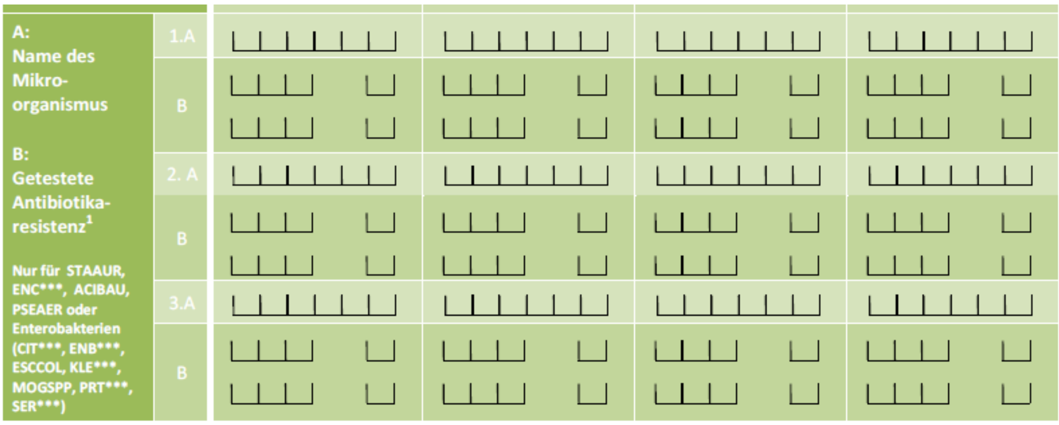

[1]Resistenzen, die hier berücksichtigt werden:
STAAUR: Oxacillin (OXA) oder Glycopeptide (GLY);
ENC***: nur GLY;
Enterobakterien: 3.-Generation-Cephalosporine (C3G) oder Carbapeneme (CAR);
PSEAER und ACIBAU: nur CAR.

Resistenz: S=Sensibel, I=Intermediär, R=Resistent, U=unbekannt

5. DATENÜBERMITTLUNG

5.1. SOFTWARE

Zur Eingabe der Daten wird eine Stand-alone Software entwickelt. Diese Software kann entweder vor Ort in den Einrichtungen (LTCF-Applikation) oder national auf Ebene des NCP (NCP-Applikation) genutzt werden.

Der NCP jedes Landes kann frei darüber entscheiden, ob er/sie den Einrichtungen die LTCF-Applikation zur Verfügung stellen möchte.

Die Einrichtungen könnten ihre Daten dann ggf. selbst über die LTCF-Applikation in die Software eingeben. Der Verantwortliche für die Datenerhebung erhält ein Benutzerhandbuch, das die Installation der Software auf einem lokalen Computer und die Technik der Dateneingabe erläutert. Nachdem alle Daten vollständig eingegeben sind, wird automatisch ein Bericht über die vorläufigen Ergebnisse für die Einrichtungen generiert. Im Anschluss müssen die Daten exportiert werden, was durch Auswählen dieser Funktion im Menü geschieht. Dadurch wird automatisch eine zip-Datei erstellt, die dann von der Einrichtung an den NCP geschickt wird.

Der NCP kann entweder alle von den Einrichtungen zugeschickten zip-Dateien in die Software importieren oder er/sie gibt die Daten gesammelt selbst mithilfe der NCP-Applikation ein. Der NCP kann seine/ihre Applikation außerdem nutzen um alle nationalen Daten zu kontrollieren und auf Fehler und Vollständigkeit zu überprüfen. Notwendige Veränderungen können mithilfe der Software vorgenommen und die ATC-Codes für Antibiotika ergänzt werden. Auch die NCP-Applikation erlaubt das Generieren von Berichten über die Ergebnisse einzelner Einrichtungen.

Sobald alle Daten eingegeben und kontrolliert sind, wird mithilfe der Export-Funktion eine nationale Datenbank erstellt. Die generierte zip-Datei wird an das ECDC geschickt und dort in die Europäische Datenbank eingespeist.

5.2. DATENANALYSE UND FEEDBACK

Das ECDC wird alle nationalen Datenbanken in einer Europäischen Datenbank zusammenführen, die wiederum auf Korrektheit und Vollständigkeit überprüft wird. Ein Ergebnis-Bericht für jede teilnehmende Einrichtung wird generiert und dem NCP zur weiteren Verbreitung zur Verfügung gestellt.

Ein Europäischer Studienbericht mit aggregierten Ergebnissen für ausgewählte Arten von Einrichtungen wird vom ECDC erstellt und eine elektronische Version wird zudem auch auf der ECDC Website verfügbar sein.

5.3. EIGENTUM DER DATEN

Sofern Daten auf nationaler Ebene präsentiert werden sollen, muss dies nach Rücksprache mit den jeweiligen NCPs erfolgen.

Die NCPs sind aufgefordert, ihre Daten zu publizieren bzw. auf internationalen Konferenzen zu präsentieren. Der HALT-3-Projektgruppe und dem ECDC sollte in allen wissenschaftlichen Veröffentlichungen (einschließlich Posterbeiträgen und Vorträgen) gedankt werden.

Alle Analysen und Schlussfolgerungen, die weitere Daten als die des eigenen Landes enthalten, sollten in Absprache und im Einverständnis mit dem HALT-3 Management Team bzw. dem ECDC angefertigt werden. Alle wissenschaftlichen Schlussfolgerungen sollten dem HALT-3 Management Team mitgeteilt werden.

6. SCHULUNG

Einheitliches Schulungsmaterial für die lokale oder externe erhebende Person wird vom ECDC zur Verfügung gestellt und vom NCP am RKI übersetzt und angepasst. Es wird empfohlen, dass die NCPs bzw. nationalen/regionalen Koordinatoren der Erhebung mindestens einen Schulungstermin für die an der Erhebung teilnehmenden Langzeitpflegeeinrichtungen organisieren. DAS RKI wird in Abhängigkeit von der Zahl und Verteilung der teilnehmenden Einrichtungen in Deutschland an mind. fünf Standorten Schulungen für die Teilnehmer anbieten.

7. VALIDIERUNG UND ON-SITE-ASSESSMENT

In Kooperation mit dem wissenschaftlichen Beirat hat das Projekt-Team des ECDC eine Methode entwickelt um:

- zusätzliche Daten zur Infektionsprävention und -kontrolle, zu Erfordernissen und Herausforderungen in Langzeitpflegeeinrichtungen zu erfassen, um eine bessere Interpretation der strukturierten Daten auf Einrichtungsebene, die während der Erhebung gesammelt werden, zu ermöglichen.
- den Effekt von wiederholten Punktprävalenzstudien zum Vorkommen HAI und zum Einsatz von Antibiotika in Langzeitpflegeeinrichtungen auf lokaler aber auch auf nationaler Ebene zu interpretieren.
- Eine qualitative Bewertung der Validität der Daten, die in jedem Land gewonnen wurden, speziell im Hinblick auf die Anwendung der Definitionen, diagnostische Kapazitäten in Langzeitpflegeeinrichtungen, Zugang zu verfügbaren Datenquellen während der Erhebung (inkl. mikrobiologische Ergebnisse und Befunde), Implementierung von Definitionen von HAI in den Einrichtungen und Validität der angegebenen Struktur- und Prozessindikatoren zur Infektionsprävention und zum Antibiotic Stewardship.

EU/EEA Mitgliedsländer, die in 2016/ 2017 an HALT-3 teilnehmen werden gebeten an einem on-site assessment (ein zweitägiger Besuch) teilzunehmen.

Zusammen mit einem Experten, aus der Projektgruppe des ECDC wird der NCP mit Vertretern aus mind. einer an HALT-3 teilnehmenden Einrichtung zusammentreffen und ein strukturiertes Assessment der Methodik durchführen und die Validierungsmethodik implementieren.

Ein kurzer Bericht über jeden Validierungs-Besuch eines Landes wird vom ECDC Projekt-Team erstellt und dem jeweiligen Land zur Verfügung gestellt. Die Informationen und Daten, die während der on-site assessments gesammelt werden, werden in Datenbanken eingegeben und analysiert. Weitere Informationen enthält das „on-site-assessment-Protokoll und das Validierungs-Protokoll (HAI-Net@ecdc.europa.eu.)

8. ROLLE DES NATIONAL CONTACT POINT (NCP)

Die Rolle des NCP ist entscheidend für den Erfolg des Projekts.

Vor und während der Datenerhebung sind seine/ihre Aufgaben:

- Die Übersetzung der Studienunterlagen in die jeweilige Landessprache
- Auswählen und Einladen von Langzeitpflegeeinrichtungen, die auf freiwilliger Basis an der Erhebung teilnehmen, einschließlich einer Einrichtung, die an der Validierung teilnimmt
- Eine Liste aller teilnehmenden Einrichtungen erstellen und diese entsprechend ihrer Eigenschaften sortieren (pseudonymisiert)
- Teilnahme am zweitägigen Trainingskurs beim ECDC in Stockholm
- Organisation und Durchführung von Schulungsveranstaltungen für die teilnehmenden Langzeitpflegeeinrichtungen
- Verteilen der Studienunterlagen
- Unterstützung der Langzeitpflegeinrichtungen während der Datenerhebung (Helpdesk)

Nach der Datenerhebung sind folgende Aufgaben zu bearbeiten:

- Sammeln der Daten der teilnehmenden Langzeitpflegeeinrichtungen bzw. Dateneingabe in die nationale Datenbank und Prüfen der Daten auf Vollständigkeit und Korrektheit
- Export der nationalen Datenbank an das ECDC
- Verteilen der Feedback-Berichte an die teilnehmenden Einrichtungen
- Mitteilen der nationalen Ergebnisse z.B. auf (inter)nationalen Tagungen

1. ETHISCHE ASPEKTE

Die Ethikkommission der Ärztekammer hat über die Erhebungen im Rahmen von HALT bereits beraten und keine berufsethischen oder berufsrechtlichen Einwände geäußert.

Im Rahmen des Projekts wird der Datenschutz angemessen berücksichtigt. Der Datenschutzbeauftragte der Bundesregierung hat über die Erhebung im Rahmen von HALT beraten und keine Bedenken geäußert.

- Jeder teilnehmenden Einrichtung wird durch den NCP eine Studiennummer zugeteilt.

Die Einrichtungen sind dann nicht mehr durch Dritte identifizierbar, da Berichte, Veröffentlichungen und Präsentationen nur die Studiennummern enthalten, nicht aber die Namen der Einrichtungen.

- Jedem Bewohner, für den ein Bewohnerfragebogen ausgefüllt wird, wird eine Bewohnerstudiennummer zugeteilt.
- Es werden somit auf Einrichtungsebene vollkommen anonymisierte Daten erfasst und auf Bewohnerebene pseudonymisierte Daten
- Im Vorfeld der Erhebung sollen alle Bewohner Ihre schriftliche Einwilligung zur Erhebung geben. Dazu werden von Seiten des NCP ein Bewohnerinformationsblatt und eine Einwilligungserklärung zur Verfügung gestellt.

Die optional zu verwendenden Stationslisten, die ein Feld für die Namen der Bewohner enthalten, müssen auf sichere und vertrauliche Art in den Einrichtungen verwahrt werden. Diese Listen werden am Ende des Projekts vernichtet.

Alle Daten, die im Rahmen dieses Projekts erhoben werden, dürfen nicht für andere als in dem Protokoll angegebene Zwecke verwendet werden.

Das Robert Koch-Institut hat sich in seiner Datenschutzleitlinie den folgenden Zielen verpflichtet, die auch im Rahmen dieser Erhebung berücksichtigt werden:

- Schutz des informationellen Selbstbestimmungsrechts
- Gewährleistung der gesetzlichen Anforderungen
- Verlässlichkeit des Handelns
- Gewährleistung des guten Rufs in der Öffentlichkeit
- Korrelation mit fachspezifischen Zielen des RKI

Die Gewährleistung der gesetzlichen Anforderungen wird durch den Datenschutzbeauftragten am RKI sichergestellt. Der Datenschutzbeauftragte hat die Aufgabe, auf die Einhaltung des Bundesdatenschutzgesetz (BDSG) sowie anderer Vorschriften des Datenschutzes am RKI hinzuwirken. Er soll insbesondere auf die Wahrung der Rechte der Betroffenen bei der Verarbeitung ihrer personenbezogenen Daten achten. Hierzu kann er durch Verfahren und Richtlinien die notwendigen Maßnahmen zur nachhaltigen Umsetzung des Datenschutzes am RKI konkretisieren und deren Einhaltung kontrollieren.

(Neue Verfahren, mit denen personenbezogene Daten automatisiert verarbeitet werden sollen und die besondere Risiken für die Rechte und Freiheiten der Betroffenen aufweisen, sind nach § 4d Abs. 5 BDSG vor Beginn der Verarbeitung zu prüfen (Vorabkontrolle). Zuständig hierfür ist nach § 4d Abs. 6 Satz 1 BDSG der Datenschutzbeauftragte. Der Beauftragte hat die Vorabkontrolle nach Empfang der Übersicht nach § 4g Abs. 2 Satz 2 BDSG durchzuführen (§ 4d Abs. 6 Satz 1 und 2 BDSG).)

9. FINANZIELLE ASPEKTE

Es wird seitens des HALT-3 Management Teams oder des ECDC keine finanzielle Unterstützung zur Teilnahme an dem Projekt zur Verfügung gestellt. Reisekosten zur Teilnahme an der Schulung werden ggf. anteilig übernommen.

KONTAKTINFORMATIONEN

HALT-3 MANAGEMENT TEAM DES ECDC:

Pete Kinross (HALT-3 Project Manager), Tommi Kärki, Carl Suetens (HAI-Net Coordinator)

Email: pete.kinross@ecdc.europa.eu / HAI-Net@ecdc.europa.eu; Tel: +46 8 5860 1478; Fax: +46 8 5860 1001

Surveillance and Response Support Unit

European Centre for Disease Prevention and Control (ECDC)

Tomtebodavägen 11A, 171 83 Stockholm, Sweden

Katrien Latour

Scientific Institute of Public Health

Operational Directorate Public Health & Surveillance

Juliette Wytsmanstreet, 14

1050 – Brussels, Belgium

Tel: +32 2 642.57.62 Fax: +32 2 642.54.10

Email: katrien.latour@wiv-isp.be

NATIONAL CONTACT POINT (NCP) FÜR DEUTSCHLAND:

Dr. Claudia Ruscher
Robert Koch-Institut
Fachgebiet 14
(Angewandte Infektions-und Krankenhaushygiene)
Nordufer 20
13353 Berlin
Tel. 030/ 18754-2142
Fax 030/ 18754-3419

Dr. Nicole Schmidt
Robert Koch-Institut
Fachgebiet 37
(FG 37 Nosokomiale Infektionen, Surveillance von Antibiotikaresistenz und -verbrauch)
Seestr. 1
13353 Berlin
Tel. 030/ 18754-3207
Fax 030/ 18754-3419

Anhang 1. Einrichtungsfragebogen

Annex 2. Stationsliste *(nur für den internen Gebrauch)*

Anhang 3. Bewohnerfragebogen

Anhang 4. Definitionen für Infektionen

Anhang 5. Codeliste für Mikroorganismen
