# Supplementary material for: Healthcare-associated infections and antimicrobial use in long-term care facilities. German results of the third European point prevalence survey HALT-3
Source: Bundesgesundheitsblatt Gesundheitsforschung Gesundheitsschutz. 2022 Aug 11;65(9):863–71. [Article in German] doi: 10.1007/s00103-022-03566-3 (PMC9366828; doi:10.1007/s00103-022-03566-3)
Supplement: Supplementary file 2 [file 103_2022_3566_MOESM2_ESM.pdf]

EUROPÄISCHE PUNKTPRÄVALENZSTUDIE ZUM VORKOMMEN VON NOSOKOMIALEN INFEKTIONEN UND ZUM EINSATZ VON ANTIBIOTIKA IN LANGZEITPFLEGEEINRICHTUNGEN (HALT-3)

DEFINITIONEN UND ALGORITHMEN FÜR INFEKTIONEN

WICHTIGER HINWEIS: *Alle am Tag der Erhebung aktiven Infektionen sollten erfasst werden. Eine Infektion ist aktiv, wenn am Tag der Erhebung Beschwerden/Symptome vorliegen ODER Beschwerden/ Symptome in der Vergangenheit vorlagen und der Bewohner am Tag der Erhebung (noch) aufgrund dieser Infektion behandelt wird. In diesem Fall sollte das Vorhandensein von Beschwerden/ Symptomen in den zwei Wochen vor der Erhebung überprüft werden (z.B. in den Bewohnerakten oder durch den Hausarzt), um sicher zu sein, dass die Symptome den Definitionen für die jeweilige Infektion entspricht.*

* **Fieber**: 1) einmalig > 37.8°C oral/Trommelfell **oder** 2) wiederholt > 37.2°C oral oder > 37.5°C rektal **oder** 3) > 1.1°C über Ausgangstemperatur bei jeder Messmethode (oral, Trommelfell, axillär)
** **Leukozytose:** 1) Neutrophilie > 14.000 Leukozyten /mm^3 oder 2) Linksverschiebung (>6% Stabkernige oder > 1500 Stabkernige/mm^3)
§ **Akute Veränderung der Bewusstseinslage**: Akuter Beginn + fluktuierender Verlauf + Aufmerksamkeitsstörung UND entweder Konfusion/Verwirrtheit oder veränderte Bewusstseinslage
§§ **Akute funktionelle Verschlechterung:** Neu aufgetretene Verschlechterung der Alltagskompetenz z.B. in folgenden Bereichen: Bett-Rollstuhltransfer, Bewegung, Ankleiden, Toilettenbenutzung, persönliche Hygiene, essen (z.B. neuer Anstieg um 3 Punkte im ADL-Score bei einer Skala von 0-28) ODER erhöhte Abhängigkeit gemessen mit einem anderen Index (z.B. Barthel-Index)

HARNWEGSINFEKTIONEN

☐ BEWOHNER OHNE HARNWEGSKATHETER

BESCHWERDEN UND SYMPTOME

*MINDESTENS **EINES** DER FOLGENDEN (①; ② ODER ③ ODER) KRITERIEN MUSS ZUTREFFEN:*

☐ ① Akute Dysurie ODER akute Schmerzen/ Schwellung oder Druckschmerz der Hoden, Nebenhoden oder Prostata

☐ ② Fieber* ODER Leukozytose**

UND

Eins oder mehrere der folgenden Symptome:
- ☐ Akute Schmerzen im kostovertebralen Winkel (Nierenlager)
- ☐ Suprapubischer Schmerz/Druckschmerz
- ☐ Makrohämaturie
- ☐ Neu aufgetretene oder erhöhte Frequenz beim Wasserlassen
- ☐ Neu aufgetretener oder verstärkter Harndrang
- ☐ Neu aufgetretene oder zunehmende Inkontinenz

☐ ③ Zwei oder mehr Symptome (bei Fehlen von Fieber oder Leukozytose):
- ☐ Frequenz Wasserlassen (neu oder erhöht)
- ☐ Suprapubischer Schmerz
- ☐ Makrohämaturie
- ☐ Harndrang (neu oder verstärkt)
- ☐ Inkontinenz (neu oder zunehmend)

URINKULTUR

☐ Nicht durchgeführt, negativ oder Testergebnisse unbekannt

☐ Urinkultur angelegt UND:

☐ mind. 10^5 KbE/ml von nicht mehr als 2 Bakterienspezies im Spontanurin

ODER

☐ mind. 10^2 KbE/ml jede Art und Zahl von Mikroorganismen im Einmalkatheterurin

☐ BEWOHNER MIT HARNWEGSKATHETER

BESCHWERDEN UND SYMPTOME

*MINDESTENS **EINES** DER FOLGENDEN (①;②, ③ ODER ④) KRITERIEN MUSS ZUTREFFEN:*

☐ ① Fieber, Schüttelfrost ODER neu aufgetretene Hypotonie OHNE dass eine andere Infektion vorliegt

☐ ② Akute Veränderung der Bewusstseinslage **§** ODER akute funktionelle Verschlechterung **§§** OHNE dass eine andere Diagnose vorliegt UND Leukozytose**

☐ ③ Neu aufgetretener suprapubischer Schmerz oder Schmerzen im kostovertebralen Winkel (Nierenlager) oder Druckschmerz

☐ ④ Eitriger Ausfluss im Bereich des Katheters oder akuter Schmerz, Schwellung oder Druckschmerz der Hoden, Nebenhoden oder Prostata

URINKULTUR

☐ Nicht durchgeführt, negativ oder Testergebnisse unbekannt

☐ Urinkultur angelegt UND:

☐ mind. 10^5 KbE/ml jede Art und Zahl von Mikroorganismen aus einer Katheterprobe

---

☐ Symptome UND Urinkultur positiv: ***Infektion Bestätigt (CODE: UTI-C)***

☐ Symptome UND Urinkultur nicht angelegt, negativ oder Ergebnis unbekannt: ***Infektion wahrscheinlich (CODE: UTI-P)***

☐ Infektion wird am Erhebungstag behandelt, aber es gibt keine Dokumentation über Beschwerden/Symptome *(nur bei Infektionen aus Krankenhäusern/ anderen Pflegeeinrichtungen)*: ***Infektion importiert (CODE: UTI-I)***

ATEMWEGSINFEKTIONEN

ERKÄLTUNG ODER PHARYNGITIS

*MINDESTENS **ZWEI** DER FOLGENDEN KRITERIEN MÜSSEN ZUTREFFEN:*

- ☐ Laufende Nase oder Niesen
- ☐ Verstopfte Nase
- ☐ Halsschmerzen oder Heiserkeit oder Schluckbeschwerden
- ☐ Trockener Husten
- ☐ Geschwollene oder schmerzhafte Lymphknoten im Hals-Nackenbereich (zervikale Lymphadenopathie)

- ☐ Erforderliche Kriterien erfüllt: ***Infektion bestätigt (CODE: COLD-C)***
- ☐ Infektion wird am Erhebungstag behandelt, aber es gibt keine Dokumentation über Beschwerden/Symptome *(nur bei Infektionen aus Krankenhäusern/ anderen Pflegeeinrichtungen)*: ***Infektion importiert (CODE: COLD-I)***

INFLUENZA

Die Diagnose kann auch außerhalb der Influenzasaison gestellt werden

__BEIDE__ DER FOLGENDEN KRITERIEN MÜSSEN ZUTREFFEN:

- ☐ Fieber *(siehe Definition Seite 1)* **UND**
- ☐ Mindestens drei der folgenden Kriterien:
  - ☐ Schüttelfrost
  - ☐ Neu aufgetretene Kopf- oder Augenschmerzen
  - ☐ Myalgien oder Gliederschmerzen
  - ☐ Unwohlsein oder Appetitlosigkeit
  - ☐ Halsschmerzen
  - ☐ Neu aufgetretener oder zunehmend trockener Husten

- ☐ Erforderliche Kriterien erfüllt: ***Infektion bestätigt (CODE: FLU-C)***
- ☐ Infektion wird am Erhebungstag behandelt, aber es gibt keine Dokumentation über Beschwerden/Symptome *(nur bei Infektionen aus Krankenhäusern/ anderen Pflegeeinrichtungen)*: ***Infektion importiert (CODE: FLU-I)***

INFEKTION DER UNTEREN ATEMWEGE

- ☐ Bewohner **mit** dem Befund einer Pneumonie oder eines neuen Infiltrats in der Röntgenthoraxaufnahme

BESCHWERDEN UND SYMPTOME

__BEIDE__ DER FOLGENDEN KRITERIEN MÜSSEN ZUTREFFEN:

- ☐ Mindestens **eines** der respiratorischen Beschwerden/ Symptome:
  - ☐ Neu aufgetretener oder vermehrter Husten
  - ☐ Neu aufgetretene oder vermehrte Sputumproduktion
  - ☐ Sauerstoffsättigung < 94% oder >3% niedriger als der Ausgangswert
  - ☐ Auffälliger Untersuchungsbefund der Lunge (neu aufgetreten oder verändert)
  - ☐ Pleuritische Thoraxschmerzen
  - ☐ Atemfrequenz > 25 Atemzüge/Min

UND

- ☐ Ein oder mehrere Allgemeinsymptome *(Fieber, Leukozytose, Verwirrtheit, akute funktionelle Verschlechterung; siehe Definition Seite 1)*

- ☐ Bewohner **ohne** den Befund einer Pneumonie oder eines neuen Infiltrats in der Röntgenthoraxaufnahme **ODER** es wurde keine Röntgenthoraxaufnahme durchgeführt

BESCHWERDEN UND SYMPTOME

__BEIDE__ DER FOLGENDEN KRITERIEN MÜSSEN ZUTREFFEN:

- ☐ Mindestens **zwei** der respiratorischen Beschwerden/ Symptome:
  - ☐ Neu aufgetretener oder vermehrter Husten
  - ☐ Neu aufgetretene oder vermehrte Sputumproduktion
  - ☐ Sauerstoffsättigung < 94% oder >3% niedriger als der Ausgangswert
  - ☐ Auffälliger Untersuchungsbefund der Lunge (neu aufgetreten oder verändert)
  - ☐ Pleuritische Brustschmerzen
  - ☐ Atemfrequenz > 25 Atemzüge/Min

UND

- ☐ Ein oder mehrere Allgemeinsymptome *(Fieber, Leukozytose, Verwirrtheit, akute funktionelle Verschlechterung; siehe Definition Seite 1)*

Fehlen anderer Erkrankungen, wie z. B. chronische Herzinsuffizienz, die die Symptome begründen könnten.

- ☐ Kriterien für Beschwerden/Symptome erfüllt UND Röntgenthoraxaufnahme positiv: ***PNEUMONIE BESTÄTIGT (CODE: PNEU-C)***
- ☐ Infektion wird am Erhebungstag behandelt, aber es gibt keine Dokumentation über Beschwerden/Symptome *(nur bei Infektionen aus Krankenhäusern/ anderen Pflegeeinrichtungen)*: ***PNEUMONIE IMPORTIERT (CODE: PNEU-I)***

- ☐ Erforderliche Kriterien erfüllt: ***INFEKTION BESTÄTIGT (CODE: LRTI-C)***
- ☐ Infektion wird am Erhebungstag behandelt, aber es gibt keine Dokumentation über Beschwerden/Symptome *(nur bei Infektionen aus Krankenhäusern/ anderen Pflegeeinrichtungen)*: ***INFEKTION IMPORTIERT (CODE: LRTI-I)***

HAUTINFEKTIONEN

HAUT-/BINDEGEWEBE-/WEICHGEWEBE-/WUND-INFEKTIONEN

*MINDESTENS **EINES** DER FOLGENDEN (① **ODER** ②) KRITERIEN MUSS ZUTREFFEN:*

- ☐ ① Eitrige Wunde, Haut oder Weichgewebe
- ☐ ② Vier oder mehr neu aufgetretene oder zunehmende Beschwerden/Symptome an der betroffenen Stelle:
  - ☐ Überwärmung
  - ☐ Druckschmerz oder Schmerzen
  - ☐ Rötung
  - ☐ Seröse Sekretion
  - ☐ Schwellung
  - ☐ Ein oder mehrere Allgemeinsymptome

(Fieber, Leukozytose, Verwirrtheit, akute funktionelle Verschlechterung; siehe Definition Seite 1)

☐ Erforderliche Kriterien erfüllt:

INFEKTION BESTÄTIGT (CODE: SKIN-C)

☐ Infektion wird am Erhebungstag behandelt, aber es gibt keine Dokumentation über Beschwerden/Symptome *(nur bei Infektionen aus Krankenhäusern/ anderen Pflegeeinrichtungen):*

INFEKTION IMPORTIERT (CODE: SKIN-I)

Bitte beachten: Wenn die Infektion eine der Definitionen aus dem Abschnitt „postoperative Wundinfektionen" erfüllt, geben Sie bitte diesen Definitionen Vorrang und tragen die Infektion nur dort ein. Verwenden Sie nicht zwei verschiedenen Definitionen für dieselbe Infektion

SKABIES (Krätzmilbenbefall)

***BEIDE** DER FOLGENDEN KRITERIEN (① **UND** ②)MÜSSEN ZUTREFFEN:*

☐ ① Makulopapulöser und/oder juckender Ausschlag

UND

☐ ② Mindestens eines der folgenden Kriterien:
- ☐ Ärztliche Diagnose
- ☐ Befund durch Labor bestätigt (Hautgeschabsel oder Biopsie positiv)
- ☐ Epidemiologische Verknüpfung zu Skabies-Fall mit Laborbestätigung

☐ Erforderliche Kriterien erfüllt:

INFEKTION BESTÄTIGT (CODE: SCAB-C)

☐ Infektion wird am Erhebungstag behandelt, aber es gibt keine Dokumentation über Beschwerden/Symptome *(nur bei Infektionen aus Krankenhäusern/ anderen Pflegeeinrichtungen):*

INFEKTION IMPORTIERT (CODE: SCAB-I)

HERPES SIMPLEX ODER HERPES ZOSTER INFEKTION

***BEIDE** DER FOLGENDEN KRITERIEN MÜSSEN ZUTREFFEN:*

☐ Bläschenförmiger Ausschlag

UND

☐ Ärztliche Diagnose oder Befund durch Labor bestätigt

☐ Erforderliche Kriterien erfüllt: ***INFEKTION BESTÄTIGT (CODE: HERP-C)***

☐ Infektion wird am Erhebungstag behandelt, aber es gibt keine Dokumentation über Beschwerden/Symptome *(nur bei Infektionen aus Krankenhäusern/ anderen Pflegeeinrichtungen):*

INFEKTION IMPORTIERT (CODE: HERP-I)

PILZINFEKTION

***BEIDE** DER FOLGENDEN KRITERIEN MÜSSEN ZUTREFFEN:*

☐ Charakteristischer Ausschlag oder Hautläsionen

UND

☐ Ärztliche Diagnose oder Pilznachweis im Hautgeschabsel oder in der Biopsie im Laborbefund bestätigt

☐ Erforderliche Kriterien erfüllt: ***INFEKTION BESTÄTIGT (CODE: FUNG-C)***

☐ Infektion wird am Erhebungstag behandelt, aber es gibt keine Dokumentation über Beschwerden/Symptome *(nur bei Infektionen aus Krankenhäusern/ anderen Pflegeeinrichtungen):*

INFEKTION IMPORTIERT (CODE: FUNG-I)

POSTOPERATIVE WUNDINFEKTIONEN

Infektion tritt innerhalb von 30 Tagen nach der Operation auf, wenn es keine Implantatoperation war.
Bei Implantatoperationen werden alle Infektionen mitgezählt, die innerhalb von 3 Monaten nach der Operation auftreten

OBERFLÄCHLICHE WUNDINFEKTION

__BEIDE__ DER FOLGENDEN KRITERIEN (① UND ②) MÜSSEN ZUTREFFEN:

☐ ① Infektion betrifft nur Haut oder subkutanes Gewebe

UND

☐ ② Mindestens eins der drei folgenden:

- ☐ Eitrige Sekretion aus der oberflächlichen Inzision *(mit oder ohne Laborbestätigung)*
- ☐ Kultureller Nachweis von Erregern aus einem aseptisch entnommenen Wundsekret oder Gewebe von der oberflächlichen Inzision
- ☐ Eines der folgenden Anzeichen:
  - ☐ Schmerz oder Berührungs-empfindlichkeit,
  - ☐ lokalisierte Schwellung,
  - ☐ Rötung,
  - ☐ Überwärmung,

  UND

  Chirurg oder Hausarzt öffnet die oberflächliche Inzision bewusst

☐ Erforderliche Kriterien erfüllt: ***INFEKTION BESTÄTIGT (CODE: SSSI-C)***

☐ Infektion wird am Erhebungstag behandelt, aber es gibt keine Dokumentation über Beschwerden/ Symptome *(nur bei Infektionen aus Krankenhäusern/ anderen Pflegeeinrichtungen)*: ***INFEKTION IMPORTIERT (CODE: SSSI-I)***

TIEFE WUNDINFEKTION

__BEIDE__ DER FOLGENDEN KRITERIEN (① UND ②) MÜSSEN ZUTREFFEN:

☐ ① Infektion scheint mit der Operation in Verbindung zu stehen und betrifft tiefes Gewebe (Faszien, Muskeln) der Inzision

UND

☐ ② Mindestens eins der vier folgenden:

- ☐ Eitrige Sekretion aus der tiefen Inzision aber nicht aus dem operierten Organ bzw. der Körperhöhle
- ☐ Spontan oder vom Chirurgen bewusst geöffnet, wenn der Patient mindestens eines der nachfolgenden Symptome hat: Fieber (> 38 °C), lokalisierter Schmerz oder Berührungs-empfindlichkeit
  (außer es liegt eine negative mikrobiologische Kultur aus der Tiefe der Inzision vor)
- ☐ Ein Abszess oder sonstige Zeichen einer Infektion der tieferen Schichten, sind bei der klinischen Untersuchung, während einer erneuten Operation, bei histo-pathologischen oder radiologischen Untersuchungen ersichtlich.
- ☐ Diagnose einer tiefen Wundinfektion durch den Chirurgen oder behandelnden Arzt

☐ Erforderliche Kriterien erfüllt: ***INFEKTION BESTÄTIGT (CODE: DSSI-C)***

☐ Infektion wird am Erhebungstag behandelt, aber es gibt keine Dokumentation über Beschwerden/Symptome *(nur bei Infektionen aus Krankenhäusern/ anderen Pflegeeinrichtungen)*: ***INFEKTION IMPORTIERT (CODE: DSSI-I)***

ORGANE UND KÖRPERHÖHLEN

__BEIDE__ DER FOLGENDEN KRITERIEN (① UND ②) MÜSSEN ZUTREFFEN:

☐ ① Infektion scheint mit der Operation in Verbindung zu stehen und erfasst Organe oder Körperhöhlen, die während der Operation geöffnet wurden oder an denen manipuliert wurden

UND

☐ ② Mindestens eins der vier folgenden:

- ☐ Eitrige Sekretion aus einer Drainage, die Zugang zu dem Organ bzw. der Körperhöhle im Operationsgebiet hat
- ☐ Kultureller Nachweis von Erregern aus einem aseptisch entnommenen Wundsekret oder Gewebe aus einem Organ bzw. der Körperhöhle im Operationsgebiet
- ☐ Ein Abszess oder sonstige Zeichen einer Infektion des Organs bzw. der Körperhöhle im Operationsgebiet bei klinischer Untersuchung, während der erneuten Operation, bei der histo-pathologischen oder radiologischen Untersuchung
- ☐ Diagnose des behandelnden Arztes

☐ Erforderliche Kriterien erfüllt: ***INFEKTION BESTÄTIGT (CODE: OSSI-C)***

☐ Infektion wird am Erhebungstag behandelt, aber es gibt keine Dokumentation über Beschwerden/Symptome *(nur bei Infektionen aus Krankenhäusern/ anderen Pflegeeinrichtungen)*: ***INFEKTION IMPORTIERT (CODE: OSSI-I)***

Bitte beachten: Wenn die Infektion eine der Definitionen aus dem Abschnitt „postoperative Wundinfektionen“ erfüllt, geben Sie bitte diesen Definitionen Vorrang und tragen die Infektion nur dort ein. Verwenden Sie nicht zwei verschiedenen Definitionen für dieselbe Infektion

GATROINTESTINALE INFEKTIONEN

GASTROENTERITIS

*MINDESTENS **EINES** DER FOLGENDEN (①, ② ODER ③) KRITERIEN MUSS ZUTREFFEN:*

☐ ① Durchfall: Drei oder mehr flüssige oder wässrige Stühle innerhalb von 24 Stunden über der normalen Basisfrequenz des Bewohners

☐ ② Erbrechen: Zwei oder mehrmalig innerhalb von 24 Stunden

☐ ③ Beide der folgenden Kriterien:

☐Positive Stuhlprobe für bakterielle oder virale Pathogene

UND

☐Mindestens eine der folgenden Kriterien: Übelkeit, Erbrechen, Bauchschmerzen oder Druckschmerz, Durchfall

☐ Erforderliche Kriterien erfüllt: ***INFEKTION BESTÄTIGT (CODE: GE-C)***

☐ Infektion wird am Erhebungstag behandelt, aber es gibt keine Dokumentation über Beschwerden/ Symptome *(nur bei Infektionen aus Krankenhäusern/ anderen Pflegeeinrichtungen):*
INFEKTION IMPORTIERT (CODE: GE-I)

CLOSTRIDIUM DIFFICILE INFEKTION

***BEIDE** DER FOLGENDEN KRITERIEN MÜSSEN ZUTREFFEN:*

☐ Mindestens eines der folgenden Kriterien:
Durchfall (drei oder mehr flüssige oder wässrige Stühle innerhalb von 24 Stunden) über der normalen Basisfrequenz des Bewohners ODER röntgenologischer Befund eines toxischen Megakolons

UND

☐ Mindestens eines der folgenden Kriterien:

☐ Positive Stuhlprobe für Toxin A oder B, oder positive CD Kultur oder positive PCR

☐ Befund einer pseudomembranösen Kolitis in der Endoskopie, OP oder Biopsie

☐ Erforderliche Kriterien erfüllt: ***INFEKTION BESTÄTIGT (CODE: CDI-C)***

☐ Infektion wird am Erhebungstag behandelt, aber es gibt keine Dokumentation über Beschwerden/ Symptome *(nur bei Infektionen aus Krankenhäusern/ anderen Pflegeeinrichtungen):*
INFEKTION IMPORTIERT (CODE: CDI-I)

BLUTSTROMINFEKTIONEN

*MINDESTENS **EINES** DER FOLGENDEN (① ODER ②)* KRITERIEN MUSS ZUTREFFEN*:*

☐ ① Zwei oder mehr positive Blutkulturen mit dem Nachweis des gleichen Mikroorganismus

☐ ② Nachweis eines Erregers in einer einzelnen Blutkultur, der nicht als Kontamination gilt

UND

Mindestens **eines** der folgenden Kriterien:

☐ Fieber *(siehe Definition Seite 1)*

☐ Neu aufgetretene Hypothermie (<34.5° C, oder konnte nicht mit dem verwendeten Thermometer gemessen werden)

☐ Abfall des systolischen Blutdrucks um >30 mmHg vom Ausgangswert

☐ Verschlechterung der Bewusstseinslage oder des funktionellen Zustandes

☐ Erforderliche Kriterien erfüllt: ***INFEKTION BESTÄTIGT (CODE: BSI-C)***

☐ Infektion wird am Erhebungstag behandelt, aber es gibt keine Dokumentation über Beschwerden/ Symptome *(nur bei Infektionen aus Krankenhäusern/ anderen Pflegeeinrichtungen):*
INFEKTION IMPORTIERT (CODE: BSI-I)

FIEBER UNKLARER GENESE

☐ In der Krankenakte dokumentierter zwei- oder mehrfacher Nachweis von Fieber (*Definition siehe S. 1*) mit einem Mindestabstand von 12 Stunden innerhalb eines Zeitraums von 3 Tagen mit keiner bekannten infektiösen oder nicht-infektiösen Ursache

☐ Erforderliche Kriterien erfüllt: ***INFEKTION BESTÄTIGT (CODE: FUO-C)***

☐ Infektion wird am Erhebungstag behandelt, aber es gibt keine Dokumentation über Beschwerden/ Symptome *(nur bei Infektionen aus Krankenhäusern/ anderen Pflegeeinrichtungen):*
INFEKTION IMPORTIERT (CODE: FUO-I)

ANDERE INFEKTIONEN

Bitte benennen: (***CODE: OTHER)***

INFEKTIONEN VON MUND, NASE, AUGE UND OHR

KONJUNKTIVITIS

*MINDESTENS **EINES** DER FOLGENDEN (①, ② ODER ③)*
KRITERIEN MUSS ZUTREFFEN*:*

- ☐ ① Eitrige Sekretion eines oder beider Augen seit mindestens 24 Stunden
- ☐ ② Neu aufgetretene oder zunehmende Rötung der Bindehaut, mit oder ohne Juckreiz
- ☐ ③ Neu aufgetretener oder zunehmender konjunktivaler Schmerz seit mindestens 24 Stunden

Die Symptome dürfen nicht durch Allergien oder Verletzungen bedingt sein.

- ☐ Erforderliche Kriterien erfüllt: ***INFEKTION BESTÄTIGT (CODE: CONJ-C)***
- ☐ Infektion wird am Erhebungstag behandelt, aber es gibt keine Dokumentation über Beschwerden/ Symptome *(nur bei Infektionen aus Krankenhäusern/ anderen Pflegeeinrichtungen):* ***INFEKTION IMPORTIERT (CODE: CONJ-I)***

INFEKTIONEN VON GEHÖRGANG, MITTEL- UND INNENOHR (OTITIDEN)

*MINDESTENS **EINES** DER FOLGENDEN (① ODER ②)*
KRITERIEN MUSS ZUTREFFEN:

- ☐ ① Ärztliche Diagnose einer Ohrinfektion
- ☐ ② Neu aufgetretene Sekretion aus einem oder beiden Ohren (nicht eitrige Sekretion muss mit zusätzlichen Symptomen wie z. B. Ohrenschmerzen oder Rötung einhergehen)

- ☐ Erforderliche Kriterien erfüllt: ***INFEKTION BESTÄTIGT (CODE: EAR-C)***
- ☐ Infektion wird am Erhebungstag behandelt, aber es gibt keine Dokumentation über Beschwerden/ Symptome *(nur bei Infektionen aus Krankenhäusern/ anderen Pflegeeinrichtungen):* ***INFEKTION IMPORTIERT (CODE: EAR-I)***

SINUSITIS

- ☐ Ärztliche Diagnose

- ☐ Erforderliche Kriterien erfüllt: ***INFEKTION BESTÄTIGT (CODE: SINU-C)***
- ☐ Infektion wird am Erhebungstag behandelt, aber es gibt keine Dokumentation über Beschwerden/ Symptome *(nur bei Infektionen aus Krankenhäusern/ anderen Pflegeeinrichtungen):* ***INFEKTION IMPORTIERT (CODE: SINU-I)***

ORALE CANDIDIOSE

__BEIDE__ DER FOLGENDEN KRITERIEN MÜSSEN ZUTREFFEN:

- ☐ Vorhandensein von erhabenen weißlichen Belägen auf entzündeter Schleimhaut ODER Beläge auf Mundschleimhaut

UND

- ☐ Diagnostiziert durch einen Arzt oder Zahnarzt

- ☐ Erforderliche Kriterien erfüllt: ***INFEKTION BESTÄTIGT (CODE: ORAL-C)***
- ☐ Infektion wird am Erhebungstag behandelt, aber es gibt keine Dokumentation über Beschwerden/ Symptome *(nur bei Infektionen aus Krankenhäusern/ anderen Pflegeeinrichtungen):* ***INFEKTION IMPORTIERT (CODE: ORAL-I)***
