# Supplementary material for: Healthcare-associated infections and antimicrobial use in long-term care facilities. German results of the third European point prevalence survey HALT-3
Source: Bundesgesundheitsblatt Gesundheitsforschung Gesundheitsschutz. 2022 Aug 11;65(9):863–71. [Article in German] doi: 10.1007/s00103-022-03566-3 (PMC9366828; doi:10.1007/s00103-022-03566-3)
Supplement: Supplementary file 3 [file 103_2022_3566_MOESM3_ESM.pdf]

EUROPÄISCHE PUNKTPRÄVALENZSTUDIE ZUM VORKOMMEN VON INFEKTIONEN UND ZUM EINSATZ VON ANTIBIOTIKA IN LANGZEITPFLEGEEINRICHTUNGEN (HALT-3)

EINRICHTUNGSFRAGEBOGEN

ROBERT KOCH INSTITUT

Hinweis: *Jede Einrichtung, die am HALT-3 Projekt teilnimmt, wird gebeten, diesen Einrichtungsfragebogen vollständig auszufüllen. Dieser Bogen enthält wichtige Informationen zu strukturellen und funktionellen Charakteristika, beinhaltet Nennerzahlen und Informationen zum Einsatz von Antibiotika sowie zu Infektionspräventionsmaßnahmen. Aus den Erfahrungen der bisherigen HALT-Projekte empfehlen wir, dass die den Bogen ausfüllende Person selbst auch in der Einrichtung tätig ist, um ggf. weitere Ansprechpartner zur Beantwortung hinzu ziehen zu können. Dies ist besonders entscheidend für die Fragen zu Antibiotikastrategien.*

A-ALLGEMEINE INFORMATIONEN

DATUM DER ERHEBUNG |_|_|_|_| |2|0|1|6|

EINRICHTUNGS-STUDIENUMMER *(wurde vom RKI vergeben)* |_|_|_|_|_|_|_|_|

TRÄGERSCHAFT ☐ *öffentlich* ☐ *privat* ☐ *gemeinnützig*

IST QUALIFIZIERTE PFLEGE 24 VON 24 STUNDEN IN DER EINRICHTUNG VERFÜGBAR? ☐ *Ja* ☐ *Nein*

IN DER EINRICHTUNG:

Gesamtzahl der Vollzeitäquivalente von qualifiziertem Pflegepersonal .... |_|_|_|_| *Vollzeitkräfte Pflegepersonal*

Gesamtzahl der Vollzeitäquivalente von Pflegehelfern............ |_|_|_|_| *Vollzeitkräfte Pflegehelfer*

Gesamtzahl der Bewohnerzimmer............ |_|_|_|_| *Zimmer*

Gesamtzahl der Einzelzimmer .... |_|_|_|_| *Einzelzimmer*

Gesamtzahl der Einzelzimmer mit eigenem Badezimmer (Toilette und Waschbecken und/oder Dusche) .... |_|_|_|_| *Zimmer*

B-NENNERZAHLEN (CASE-MIX)

Diese Tabelle fasst, wenn sie vollständig ausgefüllt ist, die Daten aller Stationen (ggf. mithilfe der Stationslisten) für alle Bewohner der Einrichtung zusammen.

GESAMTZAHL AM TAG DER ERHEBUNG IN IHRER EINRICHTUNG:

BETTEN IN DER EINRICHTUNG *(belegte und nicht belegte Betten)*	|_|_|_|_|
BELEGTE BETTEN AM TAG DER ERHEBUNG	|_|_|_|_|
IN DIE STUDIE EINGESCHLOSSENE BEWOHNER:	
AM TAG DER ERHEBUNG (8:00 UHR) ANWESEND UND WÄHREND DER ERHEBUNG NICHT ENTLASSEN	|_|_|_|_|
BEWOHNER, DIE ÄLTER ALS 85 JAHRE SIND	|_|_|_|_|
MÄNNLICHE BEWOHNER	|_|_|_|_|
BEWOHNER MIT SYSTEMISCHER ANTIBIOTIKATHERAPIE	|_|_|_|_|
BEWOHNER MIT BESCHWERDEN ODER SYMPTOMEN EINER INFEKTION	|_|_|_|_|
BEWOHNER MIT HARNWEGSKATHETER	|_|_|_|_|
BEWOHNER MIT GEFÄßKATHETER	|_|_|_|_|
BEWOHNER MIT DEKUBITUS	|_|_|_|_|
BEWOHNER MIT ANDEREN WUNDEN	|_|_|_|_|
ÖRTLICH UND/ODER ZEITLICH DESORIENTIERTE BEWOHNER	|_|_|_|_|

BEWOHNER, DIE BETTLÄGERIG SIND ODER EINEN ROLLSTUHL BENÖTIGEN	|__|__|__|__|
BEWOHNER MIT EINER OPERATION IN DEN ZURÜCKLIEGENDEN 30 TAGEN	|__|__|__|__|
BEWOHNER MIT INKONTINENZ (HARN UND/ODER STUHL)	|__|__|__|__|

C-MEDIZINISCHE VERSORGUNG UND IHRE KOORDINATION

1. Die medizinische Versorgung, einschließlich der Verschreibung von Antibiotika, erfolgt in der Einrichtung:

☐ *Ausschließlich durch persönliche Hausärzte oder (hausärztliche) Gemeinschaftspraxen*

☐ *Ausschließlich durch in der Einrichtung angestelltes medizinisches Personal*

☐ *In Kombination aus beiden Versorgungsformen*

2. Werden die medizinischen Maßnahmen in der Einrichtung durch einen bestimmten Arzt koordiniert?

☐ *Nein, es gibt keine interne oder externe Koordination der medizinischen Maßnahmen*

☐ *Ja, ein in der Einrichtung angestellter Arzt (intern) koordiniert die medizinischen Maßnahmen*

☐ *Ja, ein nicht in der Einrichtung angestellter Arzt (extern, z.B. ein Hausarzt) koordiniert die medizinischen Maßnahmen*

☐ *Ja, sowohl ein in der Einrichtung angestellter als auch ein externer Arzt koordinieren die medizinischen Maßnahmen*

3. Können nachfolgende Personen die medizinischen Akten/ Krankenakten aller Bewohner der Einrichtung einsehen?

Der mit der medizinischen Koordination betraute Arzt der Einrichtung	☐ *Ja*	☐ *Nein*
Die Pflegekräfte	☐ *Ja*	☐ *Nein*

D-INFEKTIONSKONTROLLMMAßNAHMEN

1. Gibt es in der Einrichtung in <u>Infektionsprävention und –kontrolle geschulte</u> Personen, die dem Pflegepersonal unterstützend zur Verfügung stehen?

☐ *Ja* ☐ *Nein*

2. Wenn ja, ist/ sind diese Person/en:

☐ *Examinierte Pflegekräfte* ☐ *Ärzte* ☐ *Sowohl als auch (Arzt und Pflegekraft)*

Diese Person/en:

☐ *Arbeitet/ arbeiten in der Einrichtung (intern)*

☐ *Ist/sind nicht in der Einrichtung tätig/angestellt (extern)*

☐ *Es gibt sowohl interne als auch externe Fachkräfte*

3. Sind in der Einrichtung folgende Aspekte gewährleistet?

(Bitte füllen Sie diesen Teil auch aus, wenn keine besonders geschulte Person in Ihrer Einrichtung verfügbar ist)

☐ *Schulungen und Fortbildungen zur Infektionsprävention und –kontrolle für Pflegekräfte und Pflegehelfer/innen*

☐ *Schulungen und Fortbildungen zur Infektionsprävention und –kontrolle für Haus- und medizinisches Personal*

☐ *Entwicklung von Pflegestandards*

☐ *Erfassung von Bewohnern, die mit multiresistenten Erregern kolonisiert/infiziert sind*

☐ *Benennung einer zuständigen Person für die Meldung und das Management von Ausbrüchen*

☐ *Rückmeldung von Surveillance-Ergebnissen an das pflegerische und medizinische Personal der Einrichtung*

☐ *Beaufsichtigung/ Kontrolle der Aufbereitung von Medizinprodukten und Pflegeartikeln (Desinfektion /Sterilisation)*

☐ *Entscheidung über Isolierungs- und besondere Barrieremaßnahmen für Bewohner, die mit multiresistenten Erregern besiedelt sind*

☐ *Das Angebot von jährlichen Influenzaimpfungen für alle Bewohner*

☐ *Regelmäßige Organisation, Kontrolle und Rückmeldung von Maßnahmen der Händehygiene in der Einrichtung*

☐ *Regelmäßige Organisation, Kontrolle und Rückmeldung über die Überprüfung oder Audits von etablierten Infektionspräventionsstrategien*

☐ *Keiner der oben aufgeführten Punkte*

4. Gibt es in der Einrichtung eine Hygienekommission (intern oder extern)? ☐ *Ja* ☐ *Nein*

5. Wie viele Treffen der Hygienekommission gab es im letzten Jahr?

Gesamtanzahl im letzten Jahr |_|_|_| *Treffen*

6. Kann die Einrichtung Hilfe und Expertise bei externen Hygienikern/Infektiologen formell erbitten/ einholen (z.B. Hygieniker eines lokalen Krankenhauses)?

☐ *Ja* ☐ *Nein*

7. Gibt es in der Einrichtung schriftliche Arbeitsanweisungen zu:

- dem Umgang mit MRSA und/oder anderen multiresistenten Erregern	☐ *Ja*	☐ *Nein*
- Maßnahmen der Händehygiene	☐ *Ja*	☐ *Nein*
- dem Umgang mit Harnwegskathetern	☐ *Ja*	☐ *Nein*
- dem Umgang mit Gefäßkathetern	☐ *Ja*	☐ *Nein*
- den Umgang mit Sonden/Sondenernährung	☐ *Ja*	☐ *Nein*

8. Gibt es ein Surveillancesystem zur Erfassung und Bewertung von nosokomialen Infektionen in der Einrichtung?
(z.B. jährlicher zusammenfassender Bericht zur Häufigkeit von Harnwegsinfektionen, Atemwegsinfektionen, etc…)

☐ *Ja* ☐ *Nein*

9. Welche der folgenden Produkte stehen in der Einrichtung zur Händehygiene zur Verfügung?

- Alkoholisches Händedesinfektionsmittel	☐ *Ja*	☐ *Nein*
- Alkoholhaltige Händedesinfektionstücher	☐ *Ja*	☐ *Nein*
- Flüssigseife (antiseptisch/andere)	☐ *Ja*	☐ *Nein*
- Stückseife	☐ *Ja*	☐ *Nein*

10. Welche Methode zur Händehygiene wird am häufigsten in der Einrichtung angewendet, <u>wenn die Hände nicht</u>

sichtbar verschmutzt sind (nur eine Antwort)?

☐ *Hygienische Händedesinfektion mit alkoholhaltigem Händedesinfektionsmittel*

☐ *Händewaschen mit Wasser und Seife (nicht antiseptisch)*

☐ *Händewaschen mit Wasser und antiseptischer Seife*

11. Wie viele Liter alkoholischen Händedesinfektionsmittels wurden im letzten Jahr (2015) in der Einrichtung verbraucht?

Gesamtverbrauch in Litern |_|_|_|_| *Liter/2015*

12. Wurden im letzten Jahr Schulungen/Fortbildungen zur Händehygiene für das Personal der Einrichtung durchgeführt?

☐ *Ja* ☐ *Nein*

13. Falls Sie im Rahmen einer Händehygiene-Kampagne, die Händehygiene-Compliance in Ihrer Einrichtung durch Beobachtungsstudien gemessen haben, wie viele Indikationen/Momente zur Händedesinfektion wurden im vergangenen Jahr in Ihrer Einrichtung beobachtet? (wenn Sie keine Beobachtung durchgeführt haben tragen Sie bitte „0“ ein)

Anzahl beobachteter Indikationen |_|_|_|_| *Indikationen im letzten Jahr*

E-STRATEGIEN ZUM ANTIBIOTIKAEINSATZ

1. Gibt es in der Einrichtung eine Verschreibungsliste für Antibiotika, die entweder gar nicht oder nur nach Zustimmung einer verantwortlichen Person eingesetzt werden dürfen?

☐ *Ja* ☐ *Nein*

2. Wenn eine solche Liste vorhanden ist, welche Antibiotika sind nur eingeschränkt oder gar nicht zu verwenden?

☐ *Carbapeneme*

☐ *3. Generations- Cephalosporine*

☐ *Fluorochinolone*

☐ *Vancomycin*

☐ *Mupirocin*

☐ *Glycopeptide*

☐ *Breitspektrum-Antibiotika*

☐ *Intravenös verabreichte Antibiotika*

3. Welche der folgenden Strukturen gibt es in der Einrichtung?

☐ *Ein ‘Antibiotika-Komitee’/eine Antibiotika-Kommission*

☐ *Regelmäßige, jährliche Fortbildungen über den angemessenen Einsatz von Antibiotika*

☐ *Schriftliche Richtlinien/Empfehlungen für den angemessenen Einsatz von Antibiotika*

☐ *Verfügbare Daten über den jährlichen Antibiotikaverbrauch, getrennt nach Wirkstoff-Gruppen*

☐ *Ein System, das medizinisches Personal an die Wichtigkeit mikrobiologischer Diagnostik zur Auswahl des geeigneten Antibiotikums hinweist bzw. erinnert*

☐ *Lokale (für Ihre Region) Resistenzprofile/Resistenzstatistiken sind der Einrichtung bzw. den verordnenden Ärzten zugänglich*

☐ *Ein System, das die Zustimmung einer verantwortlichen Person erfordert, um Reserve-Antibiotika bzw. restriktiv zu verwendende Antibiotika zu verordnen*

☐ *Pharmakologische Beratung bei der Auswahl bzw. Verordnung von restriktiv zu verwendenden Antibiotika*

☐ *Therapierichtlinien, die auch eine Antibiotika-Liste beinhalten*

☐ *Rückmeldung der Antibiotikaverbrauchsdaten der Einrichtung an die Hausärzte/verordnenden Ärzte*

☐ *Keine der oben genannten Strukturen*

4. Sofern es schriftliche Therapierichtlinien in der Einrichtung gibt, befassen sie sich mit:

- *Atemwegsinfektionen?*	☐ *Ja*	☐*Nein*
- *Harnwegsinfektionen?*	☐ *Ja*	☐ *Nein*
- *Wund- und Weichgewebeinfektionen?*	☐ *Ja*	☐ *Nein*

5. Verwenden Sie Urinteststreifen für die Erkennung von Harnwegsinfektionen?

☐ *Routinemäßig* ☐ *Manchmal* ☐ *Nie*

6. Gibt es ein System (z.B. ein Computerprogramm) zur Erfassung und Bewertung des Antibiotikaverbrauchs und zur Rückmeldung der Verbrauchsdaten in der Einrichtung ?

☐*Ja* ☐ *Nein*

7. Gibt es ein System für die Surveillance (Erfassung und Bewertung) von multiresistenten Erregern in der Einrichtung?
(z.B. eine jährliche zusammenfassende Übersicht hinsichtlich MRSA, Clostridium difficile etc.)

☐ *Ja* ☐ *Nein*

8. Woher beziehen Sie Antibiotika in Ihrer Einrichtung? (Nur eine Antwort ist hier möglich!)

☐ *aus verschiedenen Apotheken*

☐ *aus nur einer Apotheke*

☐ *Die Einrichtung bezieht Antibiotika überhaupt nicht direkt aus Apotheken; Antibiotika werden von den Bewohnern direkt bezogen (z.B. auch durch Familien)*

10.Mit wie vielen mikrobiologischen Laboren arbeitet Ihre Einrichtung? (Nur eine Antwort ist hier möglich!)

☐ *mit mehr als einem*

☐ *mit einem einzigen*

☐ *Die Einrichtung arbeitet überhaupt nicht direkt mit mikrobiologischen Laboren zusammen; jeder Hausarzt kann mit einem Labor seiner Wahl zusammenarbeiten.*

F-WIE WURDE DIE ERHEBUNG IN IHRER EINRICHTUNG DURCHGEFÜHRT?

1. Wer erfasste die HALT-3 Daten in Ihrer Einrichtung (einschl. Einrichtungs- und Bewohnerfragebogen)?

☐ *Ein Arzt*

☐ *Eine Pflegekraft*

☐ *Eine andere Person*

2. Wenn kein Arzt in die HALT-3 Datenerfassung involviert war (Einrichtungs- und Bewohnerfragebogen), wurden die Daten durch einen Arzt bestätigt?

☐ *Ja* ☐ *Nein*

IM NAMEN DES HALT-3-TEAMS DANKEN WIR IHNEN FÜR IHRE TEILNAHME!
