# Supplementary material for: Healthcare-associated infections and antimicrobial use in long-term care facilities. German results of the third European point prevalence survey HALT-3
Source: Bundesgesundheitsblatt Gesundheitsforschung Gesundheitsschutz. 2022 Aug 11;65(9):863–71. [Article in German] doi: 10.1007/s00103-022-03566-3 (PMC9366828; doi:10.1007/s00103-022-03566-3)
Supplement: Supplementary file 4 [file 103_2022_3566_MOESM4_ESM.pdf]

BEWOHNER-STUDIENNUMMER |_|_|_|_|_|_|

EUROPÄISCHE PUNKTPRÄVALENZSTUDIE ZUM VORKOMMEN VON NOSOKOMIALEN INFEKTIONEN UND ZUM EINSATZ VON ANTIBIOTIKA IN LANGZEITPFLEGEEINRICHTUNGEN (HALT-3)

BEWOHNERFRAGEBOGEN

BEWOHNERDATEN

Geschlecht	☐ Männlich	☐ Weiblich
Geburtsjahr	|_|_|_|_|	
Aufenthalt in dieser Einrichtung	☐ Kürzer als 1 Jahr	☐ 1 Jahr oder länger
Krankenhausaufenthalt in den letzten 3 Monaten	☐ Ja	☐ Nein
Operation in den letzten 30 Tagen	☐ Ja	☐ Nein
Harnwegskatheter	☐ Ja	☐ Nein
Gefäßkatheter	☐ Ja	☐ Nein
Inkontinenz (Harn und/oder Stuhl)	☐ Ja	☐ Nein
Wunden:		
o Dekubitus	☐ Ja	☐ Nein
o Andere Wunden	☐ Ja	☐ Nein
Zeitlich und/oder örtlich Desorientiertheit	☐ Ja	☐ Nein
Mobilität	☐Selbstständig ☐Rollstuhl	☐Bettlägerig

AM TAG DER ERHEBUNG:

☐ ***ERHÄLT DER BEWOHNER EINE SYSTEMISCHE ANTIBIOTIKATHERAPIE***

>BITTE TEIL A DES FRAGEBOGENS AUSFÜLLEN

einschließlich: (i) Bewohner mit prophylaktischer Antibiotikabehandlung

ODER *(ii) Bewohner mit therapeutischer Antibiotikabehandlung (bei Behandlungsbeginn vor Aufnahme sollten keine Beschwerden/Symptome aufgezeichnet werden)*

☐ ***ZEIGT DER BEWOHNER BESCHWERDEN/SYMPTOME EINER INFEKTION***

->BITTE TEIL B DES FRAGEBOGENS AUSFÜLLEN

*Nur Beschwerden/Symptome, die bei Aufnahme des Bewohners in die Einrichtung nicht schon vorhanden oder in Inkubation waren **UND** (noch) keine Antibiotikatherapie*

☐ ***BEIDES: ANTIBIOTIKATHERAPIE UND BESCHWERDEN/SYMPTOME EINER INFEKTION***

->BITTE TEIL A UND TEIL B DES FRAGEBOGENS AUSFÜLLEN *einschließlich: (i) Bewohner mit Beschwerden/Symptomen und mit Antibiotikatherapie am heutigen Tag (unabhängig davon ob die antibiotische Therapie im Zusammenhang mit dieser Infektion steht)* ***ODER***

(ii) Bewohner, die keine Beschwerden/Symptome mehr aufweisen, aber noch antibiotisch aufgrund dieser Infektion behandelt werden

BEWOHNER-STUDIENNUMMER | | | | | | |

TEIL A: DATEN ZUR ANTIBIOTISCHEN THERAPIE

	**ANTIBIOTIKUM 1**	**ANTIBIOTIKUM 2**	**ANTIBIOTIKUM 3**	**ANTIBIOTIKUM 4**
Name des Antibiotikums ***(Handelsname oder Wirkstoff)***	------------------	------------------	------------------	------------------
Art der Applikation	☐ oral<br>☐ parenteral *(i.m, i.v., s.c.)*<br>☐ andere	☐ oral<br>☐ parenteral *(i.m, i.v., s.c.)*<br>☐ andere	☐ oral<br>☐ parenteral *(i.m, i.v., s.c.)*<br>☐ andere	☐ oral<br>☐ parenteral *(i.m, i.v., s.c.)*<br>☐ andere
Datum Therapieende/ Kontrollunter-suchung bekannt?	☐ Nein<br>☐ Ja	☐ Nein<br>☐ Ja	☐ Nein<br>☐ Ja	☐ Nein<br>☐ Ja
Art der Behandlung	☐ Prophylaktisch<br>☐ Therapeutisch	☐ Prophylaktisch<br>☐ Therapeutisch	☐ Prophylaktisch<br>☐ Therapeutisch	☐ Prophylaktisch<br>☐ Therapeutisch
Indikation der Antibiotika-therapie	☐ *Harnwege*<br>☐ *Genitaltrakt*<br>☐ *Haut o. Wunden*<br>☐ *Atemwege*<br>☐ *Magen-/Darmtrakt*<br>☐ *Augen*<br>☐ *Ohren/Nase/Mund*<br>☐ *OP-Wunde*<br>☐ *Tuberkulose*<br>☐ *systemische Infektion*<br>☐ *Fieber unklarer Genese*<br>☐ *andere (bitte benennen)*<br>------------------	☐ *Harnwege*<br>☐ *Genitaltrakt*<br>☐ *Haut o. Wunden*<br>☐ *Atemwege*<br>☐ *Magen-/Darmtrakt*<br>☐ *Augen*<br>☐ *Ohren/Nase/Mund*<br>☐ *OP-Wunde*<br>☐ *Tuberkulose*<br>☐ *systemische Infek2tion*<br>☐ *Fieber unklarer Genese*<br>☐ *andere (bitte benennen)*<br>------------------	☐ *Harnwege*<br>☐ *Genitaltrakt*<br>☐ *Haut o. Wunden*<br>☐ *Atemwege*<br>☐ *Magen-/Darmtrakt*<br>☐ *Augen*<br>☐ *Ohren/Nase/Mund*<br>☐ *OP-Wunde*<br>☐ *Tuberkulose*<br>☐ *systemische Infektion*<br>☐ *Fieber unklarer Genese*<br>☐ *andere (bitte benennen)*<br>------------------	☐ *Harnwege*<br>☐ *Genitaltrakt*<br>☐ *Haut o. Wunden*<br>☐ *Atemwege*<br>☐ *Magen-/Darmtrakt*<br>☐ *Augen*<br>☐ *Ohren/Nase/Mund*<br>☐ *OP-Wunde*<br>☐ *Tuberkulose*<br>☐ *systemische Infektion*<br>☐ *Fieber unklarer Genese*<br>☐ *andere (bitte benennen)*<br>------------------
Wo wurde das Antibiotikum verordnet?	☐ In dieser Einrichtung<br>☐ Im Krankenhaus<br>☐ An anderer Stelle	☐ In dieser Einrichtung<br>☐ Im Krankenhaus<br>☐ An anderer Stelle	☐ In dieser Einrichtung<br>☐ Im Krankenhaus<br>☐ An anderer Stelle	☐ In dieser Einrichtung<br>☐ Im Krankenhaus<br>☐ An anderer Stelle

BEWOHNER-STUDIENNUMMER |_|_|_|_|_|_|

TEIL B: DATEN ZU INFEKTIONEN

		INFEKTION 1	INFEKTION 2	INFEKTION 3	INFEKTION 4
Infektionscode					
Wenn „andere" bitte angeben					
Am Tag der (Wieder-)Aufnahme in die Einrichtung bereits vorhanden gewesen		☐ Nein<br>☐ Ja	☐ Nein<br>☐ Ja	☐ Nein<br>☐ Ja	☐ Nein<br>☐ Ja
Datum des Beginns		__/__/__	__/__/__	__/__/__	__/__/__
Ort des Beginns		☐ Diese Einrichtung<br>☐ Eine andere Pflegeeinrichtung<br>☐ Krankenhaus<br>☐ unbekannt	☐ Diese Einrichtung<br>☐ Eine andere Pflegeeinrichtung<br>☐ Krankenhaus<br>☐ unbekannt	☐ Diese Einrichtung<br>☐ Eine andere Pflegeeinrichtung<br>☐ Krankenhaus<br>☐ unbekannt	☐ Diese Einrichtung<br>☐ Eine andere Pflegeeinrichtung<br>☐ Krankenhaus<br>☐ unbekannt
**A:** Name des Mikro-organismus<br><br>**B:** Getestete Antibiotika-resistenz<br><br>Nur für STAAUR, ENC***, ACIBAU, PSEAER oder Enterobakterien (CIT***, ENB***, ESCCOL, KLE***, MOGSPP, PRT***, SER***)	1.A				
	B				
	2. A				
	B				
	3.A				
	B				
