# Supplementary material for: Healthcare-associated infections and antimicrobial use in long-term care facilities. German results of the third European point prevalence survey HALT-3
Source: Bundesgesundheitsblatt Gesundheitsforschung Gesundheitsschutz. 2022 Aug 11;65(9):863–71. [Article in German] doi: 10.1007/s00103-022-03566-3 (PMC9366828; doi:10.1007/s00103-022-03566-3)

Onlinematerial 5. Teilnehmende Pflegeeinrichtungen nach Bundesländern.

Bundesland	Pflegeeinrichtungen (Anzahl)
Baden-Württemberg	16
Bayern	6
Berlin	1
Brandenburg	0
Bremen	0
Hamburg	2
Hessen	13
Mecklenburg-	5
Niedersachsen	2
Nordrhein-Westfalen	36
Rheinland-Pfalz	12
Saarland	20
Sachsen	0
Sachsen-Anhalt	8
Schleswig-Holstein	3
Thüringen	6

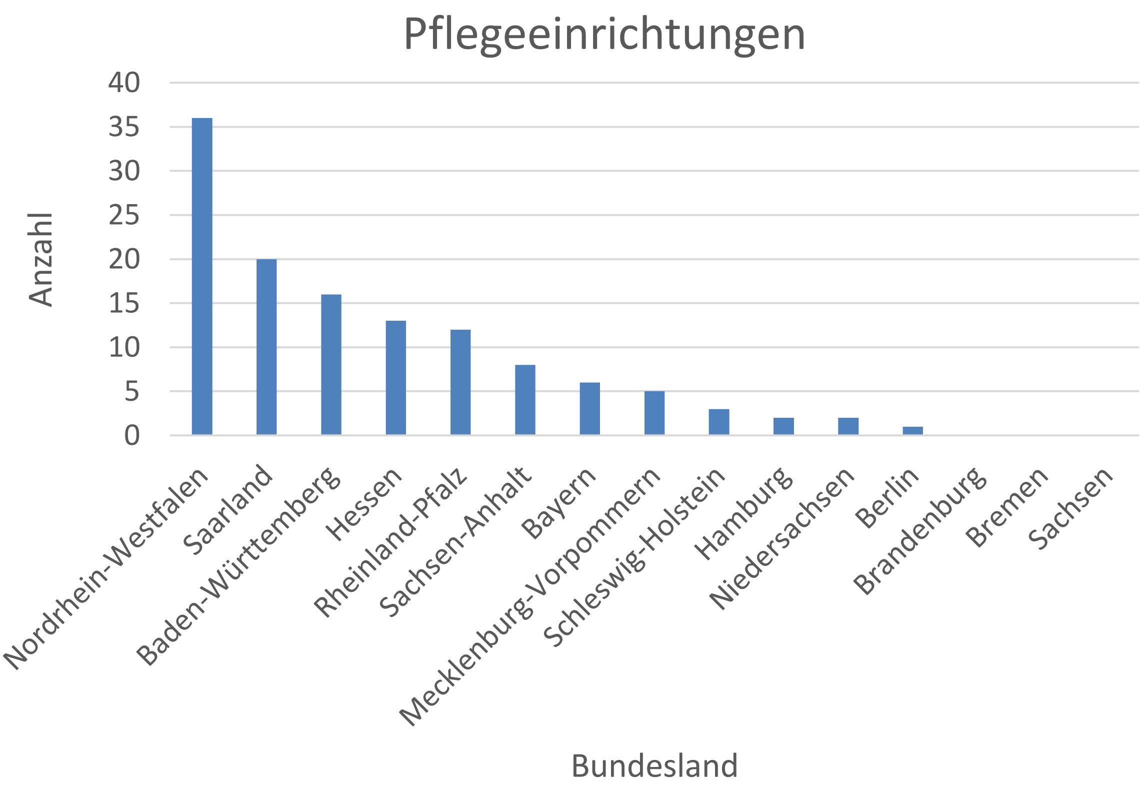

Supplement: Supplementary file 5 [file 103_2022_3566_MOESM5_ESM.pdf]
