# Supplementary material for: Healthcare-associated infections and antimicrobial use in long-term care facilities. German results of the third European point prevalence survey HALT-3
Source: Bundesgesundheitsblatt Gesundheitsforschung Gesundheitsschutz. 2022 Aug 11;65(9):863–71. [Article in German] doi: 10.1007/s00103-022-03566-3 (PMC9366828; doi:10.1007/s00103-022-03566-3)
Supplement: Supplementary file 6 [file 103_2022_3566_MOESM6_ESM.pdf]

Onlinematerial 6. Größe und Anzahl der LTCF mit Prävalenzen für NI und ABA nach Größe der LTCF.

**Größe der LTCF (Anzahl von Betten)**	**0-50**	**51-100**	**101-150**	**151-200**	**>250**
**Anzahl der LTCF**	20	70	32	7	2
**Prävalenz NI (%) (95%-KI)**	1,4 (0,6-2,8)	1,8 (1,4-2,2)	1,7 (1,3-2,1)	1,7 (1,0-2,6)	1,1 (0,4-2,4)
**Prävalenz NI in der eigenen Einrichtung erworben (%) (95%-KI)**	1,4 (0,6-2,8)	1,4 (1,1-1,7)	1,2 (0,9-1,7)	1,3 (0,7-2,1)	1,1 (0,4-2,4)
- Minimum	0,00	0,00	0,00	0,00	0,4
- Maximum	16,7	12,8	6,2	4,0	1,9
- Mittelwert	1,5	1,8	1,6	1,6	1,1
**Prävalenz ABA (%) (95%-KI)**	2,3 (1,2-3,9)	1,4 (1,1-1,7)	1,3 (1,0-1,8)	1,1 (0,6-1,9)	0,9 (0,3-2,1)
- Minimum	0,00	0,00	0,00	0,00	0,4
- Maximum	11,1	8,5	4,9	2,8	1,5
- Mittelwert	2,5	1,4	1,3	1,0	0,9

Legende: ABA - Antibiotikaanwendung; KI – Konfidenzintervall; LTCF – Langzeitpflegeeinrichtung; NI - Nosokomiale Infektionen.
